# Asymmetric Dearomative (3+2)-Cycloaddition Involving Nitro-Substituted Benzoheteroarenes under H-Bonding Catalysis †

**DOI:** 10.3390/molecules26164992

**Published:** 2021-08-18

**Authors:** Maciej Saktura, Anna Skrzyńska, Sebastian Frankowski, Sylwia Wódka, Łukasz Albrecht

**Affiliations:** Institute of Organic Chemistry, Faculty of Chemistry, Lodz University of Technology, Żeromskiego 116, 90-924 Łódź, Poland; maciej.saktura@edu.p.lodz.pl (M.S.); sebastian.frankowski@p.lodz.pl (S.F.); 218820@edu.p.lodz.pl (S.W.)

**Keywords:** organocatalysis, CADA reactions, dearomative (3+2)-cycloaddition, 2-nitrobenzofurans, azomethine ylides

## Abstract

In our studies, the organocatalytic 1,3-dipolar cycloaddition between 2-nitrobenzofurans or 2-nitrobenzothiophene and *N*-2,2,2-trifluoroethyl-substituted isatin imines has been developed. The reaction has been realized by employing bifunctional organocatalysis, with the use of squaramide derivative being crucial for the stereochemical efficiency of the process. The usefulness of the cycloadducts obtained has been confirmed in selected transformations, including aromative and non-aromative removal of the nitro group.

## 1. Introduction

Catalytic asymmetric dearomatization (CADA) reactions constitute popular strategies for the synthesis of complex natural products, biologically active compounds, and pharmaceuticals from readily available aromatic molecules [1,2,3,4,5,6,7,8,9,10]. Despite their high synthetic potential, challenges related to the control of the regio- and stereo-selectivity of these processes, while overcoming the loss of aromaticity, still constitute an important issue. 

CADA reactions are primarily focused on transformations involving structurally simple electron-rich arenes and heteroarenes, such as naphthols [11,12,13,14,15,16,17,18], phenols [19,20], indoles [21,22,23,24], and pyrroles [25,26,27,28]. The application of (hetero)aromatic reactants as electrophilic counterparts in such strategies is much less common. Recently, a novel approach based on the application of (hetero)aromatic derivatives bearing a suitable electron-withdrawing substituent in their structure emerged as a useful strategy to reverse reactivity of these systems, thus expanding the synthetic potential of CADA transformations. 

**Scheme 1:**
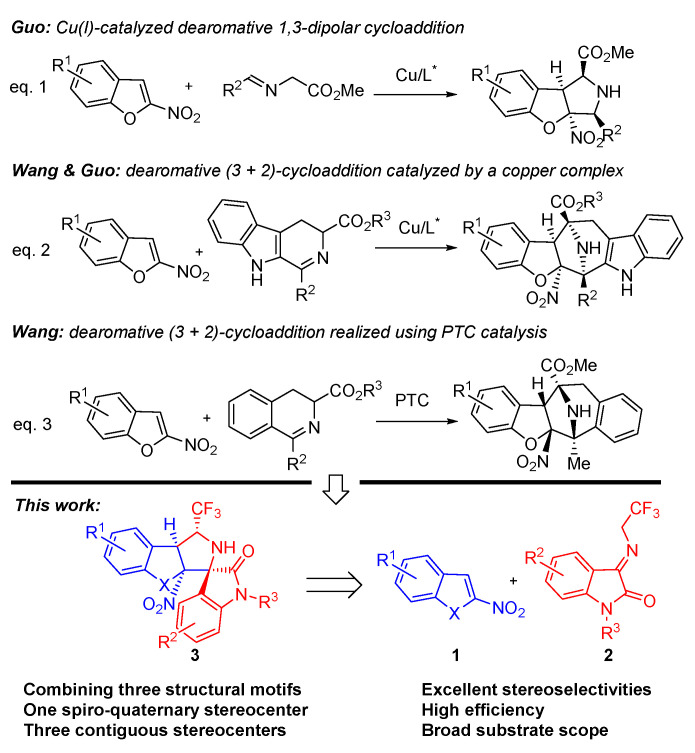
Asymmetric dearomative (3+2)-cycloaddition between nitro-substituted benzoheteroarenes **1** and *N*-2,2,2-trifluoroethyl-substituted isatin imines **2**.

CADA reactions involving electron-deficient nitro(hetero)arenes, such as 2- or 3-nitroindoles [29,30,31,32,33,34,35,36,37,38], 2-nitrobenzofurans [37,39,40,41,42,43,44], and 2- or 3-nitrobenzothiophenes [39,40,43,45,46], have recently provided a direct route to highly substituted polycyclic compounds with multiple stereogenic centers. Due to the immense biological importance of benzo-fused units, the development of new approaches for the synthesis of these compounds is highly desirable. In this context, enantioselective dearomative annulations involving nitro(hetero)arenes leading to nitrogen-containing heterocyclic compounds are in great demand. However, to the best of our knowledge, few literature reports describing the asymmetric dearomative (3+2)-cycloaddition of 2-nitrobenzofurans with azomethine ylides are available (Scheme 1). In 2019, Wang, Guo, and co-workers discovered the Cu(I)-catalyzed dearomative 1,3-dipolar cycloaddition for the construction of chiral tricyclic hydrobenzofurans for the first time (Scheme 1, eq. 1) [47]. Later, the same group developed a method for the preparation of chiral tropane derivatives via copper-catalyzed dearomative (3+2)-cycloaddition of 2-nitrobenzofurans and cyclic azomethine ylides (Scheme 1, eq. 2) [48]. Very recently, Wang et al. reported the dearomative cycloaddition for the stereoselective preparation of polycyclic benzofused tropane derivatives by employing bifunctional phosphonium salts as phase-transfer catalysts (Scheme 1, eq. 3) [49]. 

In continuation of our efforts on the development of asymmetric dearomative transformations [50,51,52,53,54,55], we became interested in CADA reactions involving electron-deficient heteroaromatic systems. Herein, we report the organocatalytic dearomative 1,3-dipolar cycloaddition between nitro-substituted benzoheteroarenes with azomethine ylides, as realized under bifunctional catalysis, yielding optically active pyrrolidine-fused spirocyclic dihydrobenzofuran and dihydrobenzothiophene derivatives bearing four contiguous stereocenters. In the context of our studies, it should be noted that a complementary approach involving bifunctional phase-transfer catalysis (PTC) was recently developed by Ren and Wang et al. [56]. 

## 2. Results and Discussion

### 2.1. Optimization Studies

Optimization studies were performed using 2-nitrobenzofuran **1a** and *N*-2,2,2-trifluoroethyl-substituted isatin imine **2a** as model reactants. Initially, quinine **4a** was employed as a catalyst and the reaction was run in CDCl_3_ for the ease of data processing (Table 1, entry 1). Pleasingly, the formation of desired 1,3-dipolar cycloadduct **3a** was observed; however, low yield and diastereoselectivity were observed. Furthermore, chiral UPC^2^ analysis of isolated product **3a** showed that the reaction proceeded without induction of asymmetry. Therefore, various Brønsted base-type catalysts were evaluated to improve effectiveness and stereoselectivity of the process. Interestingly, the use of commercially available dimeric catalysts **4b** or **4c** provided a significant increase in conversion and diastereoselectivity (Table 1, entries 2–3). Moreover, when catalyst **4c** was used, **3a** was obtained with satisfactory enantioselectivity (Table 1, entry 3). Subsequently, the influence of bifunctional catalysts **4d**–**h** on the studied transformation was examined (Table 1, entries 4–8). Performed experiments revealed that squaramide-based catalysts **4e**–**h** derived from quinine were appropriate for the developed cycloaddition (Table 1, entries 5–8). Finally, catalyst **4h** was selected for further studies, as its utilization ensured the formation of **3a** with good yield and excellent diastereo- and enantioselectivity (Table 1, entry 8). Subsequently, screening of solvents was initiated (Table 1, entries 9–15). Unfortunately, the decrease in reactivity caused by poor substrate solubility or diminished imine **2a** stability was observed. Decrease in concentration of the reaction mixture did not bring improvement in terms of yield or stereoselectivity of the transformation, but a longer reaction time was required (Table 1, entry 16). However, imine **2a** degradation was observed as the consequence of prolonged reaction time. This problem was eventually solved by the use of 1.5-fold excess of **2a**. This change resulted in almost full conversion of 2-nitrobenzofurane **1a**, and **3a** was obtained with excellent yield and stereoselectivity (Table 1, entry 17). In the hope of improving enantioselectivity, the reaction was performed at lower temperature but, disappointingly, without any enhancement of cycloadduct **3a** enantiomeric ratio (Table 1, entry 18). Moreover, 20 mol% loading of the catalyst turned out to be crucial for completion of the reaction, as its lowering led to an inhibition of the process (Table 1, entry 19). Finally, it was found that the reaction proceeded with comparable results in freshly distilled chloroform (Table 1, entry 20). It is worth noting that the presented reaction was readily scalable to one-mmol scale, affording product **3a** with a good outcome (Table 1, entry 21).

### 2.2. Scope Studies

**Scheme 2:**
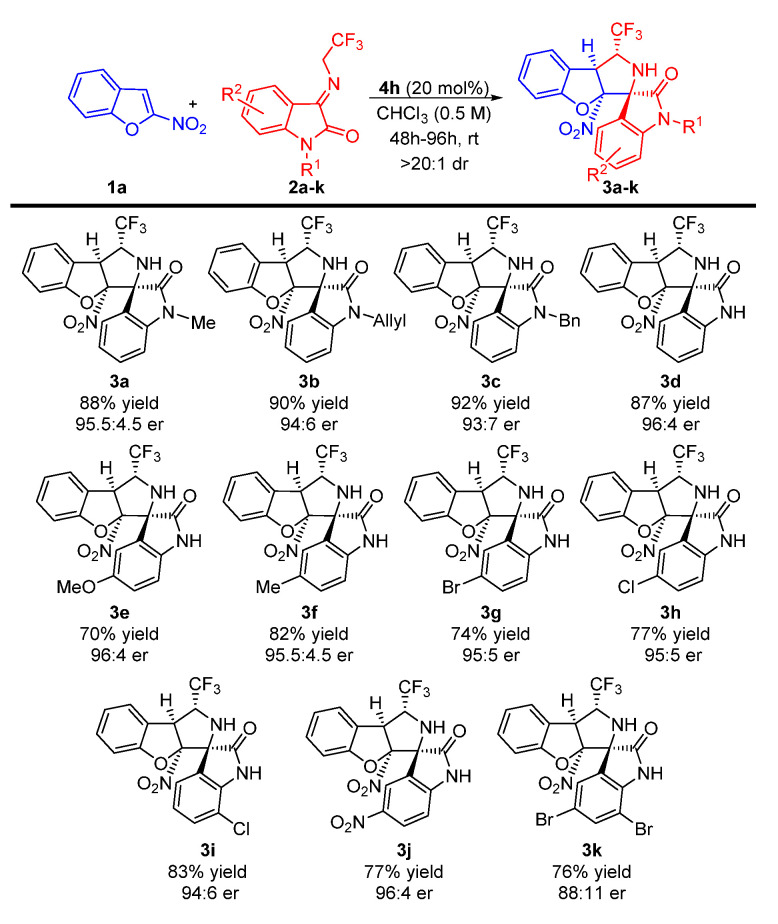
Asymmetric dearomative (3+2)-cycloaddition between nitro-substituted benzoheteroarenes **1** and *N*-2,2,2-trifluoroethyl-substituted isatin imines **2**–scope studies.

With the optimized reaction conditions in hand, the scope of the reaction was evaluated. In the first step, structurally diversified dipole precursors **2a**–**k** were tested in an organocatalytic process (Scheme 2). Imines **2a**–**c** with different protecting groups at the nitrogen atom were well tolerated in the developed (3+2)-cycloaddition, providing products **3a**–**c** in good yields and with excellent stereocontrol. Moreover, non-protected isatin-derived imine **2d** worked well, giving access to **3d** with similar results. It is worth noting that the developed reaction was unbiased towards the electronic properties of substituents in dipole precursors **2**, as products with both electron-donating (**2e**,**f**) and electron-withdrawing groups (**2g**–**j**) were efficiently obtained in a highly stereoselective manner. Moreover, imine **2k** with a double substitution pattern gave access to product **3k** as a single diastereoisomer, in high yield but with diminished enantiocontrol.

**Scheme 3:**
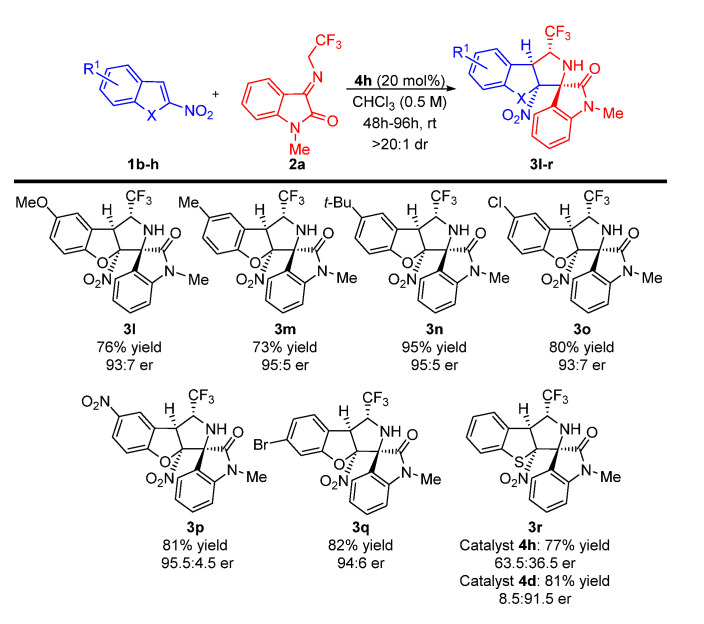
Asymmetric dearomative (3+2)-cycloaddition between nitro-substituted benzoheteroarenes **1** and *N*-2,2,2-trifluoroethyl-substituted isatin imines **2**–scope studies.

In the next step, the scope of dipolarophiles **1** was examined (Scheme 3). 2-Nitrobenzofuranes **1b**–**f** substituted in the 5-position with groups of different electron properties reacted smoothly in the developed cycloaddition, providing products **3l**–**p** in high yields and with excellent stereoselection. Notably, the bulky *tert*-butyl group in **1d** and the strongly electron-withdrawing nitro substituent in **1f** were well tolerated, as demonstrated in the synthesis of **3n** and **3p**, where the desired reaction proceeded without any loss in enantioselectivity. Notably, cycloadduct **3q** with the benzofuran ring functionalized in the 6-position was efficiently obtained following the developed method. Moreover, the substrate scope was further expanded by the use of 2-nitrobenzothiophene **1h**. Surprisingly, the developed cycloaddition provided **3r** in high yield but with poor enantiocontrol under standard conditions. Thankfully, short re-optimization studies revealed that thiourea catalyst **4d** significantly enhanced the stereocontrol affording **3r** with good enantioselectivity.

### 2.3. Synthetic Utility of Products ***3***

**Scheme 4:**
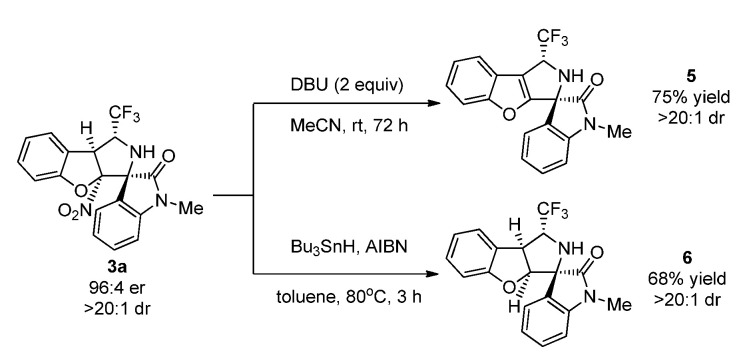
Asymmetric dearomative (3+2)-cycloaddition between nitro-substituted benzoheteroarenes **1** and *N*-2,2,2-trifluoroethyl-substituted isatin imines **2**–transformations.

With the scope studies accomplished, the usefulness of obtained products **3** was demonstrated in selected transformations (Scheme 4). Base-promoted nitro group removal gave access to spirocyclic compound **5** with aromatic benzofuran moiety. Furthermore, the reductive denitration of the starting material **3a** was easily performed by utilization of tributyltin hydride and AIBN, providing dihydrobenzofuran **6** in high yield. Notably, the stereochemical composition of the starting material **3a** was fully preserved in both cases, as products **5** and **6** were obtained as single diastereoisomers.

### 2.4. Absolute Configuration Assignment and Mechanistic Considerations

**Scheme 5:**
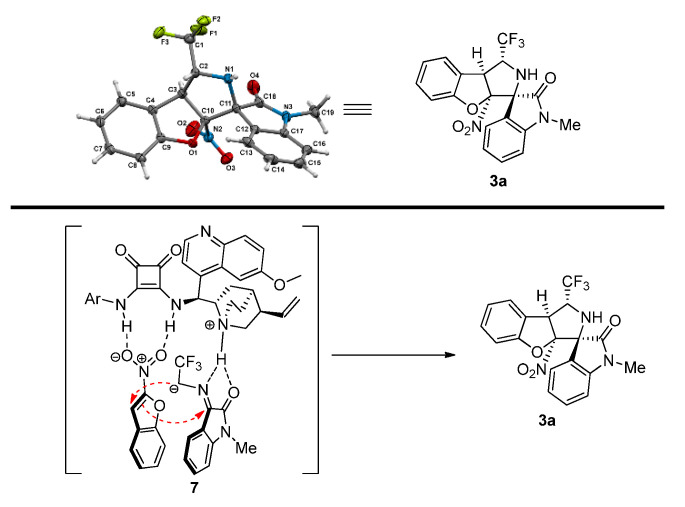
Asymmetric dearomative (3+2)-cycloaddition between nitro-substituted benzoheteroarenes **1** and *N*-2,2,2-trifluoroethyl-substituted isatin imines **2**–mechanistic considerations.

The absolute configuration of product **3a** was assigned by X-ray analysis (Scheme 5, top) [57]. The stereochemistry of products **3b**–**r** was determined by analogy. The absolute stereochemistry allowed us to propose a plausible stereochemical model of the cycloaddition (Scheme 5, bottom). The reaction is initiated by two independent processes. The substrate **1** is activated and oriented by the hydrogen bonds of squaramide moiety of the catalyst **4h**. Simultaneously, the quinuclidine moiety of the alkaloid catalyst deprotonates the *N*-2,2,2-trifluoroethyl-substituted isatin imine **2**, leading to the formation of azomethine ylide **7**. According to the proposed dual-activation model (3+2)-cycloaddition between **1** and **7** takes place providing **3** in a stereoselective manner.

## 3. Materials and Methods

### 3.1. General

NMR spectra were acquired on a Bruker Ultra Shield 700 instrument, running at 700 MHz for ^1^H and 176 MHz for ^13^C, respectively. Chemical shifts (δ) are reported in ppm relative to residual solvent signals (CDCl_3_: 7.26 ppm for ^1^H NMR, 77.16 ppm for ^13^C NMR). Mass spectra were recorded on a Bruker Maxis Impact spectrometer using electrospray (ES+) ionization referenced to the mass of the charged species. Optical rotations were measured on a PerkinElmer 241 polarimeter and [α]_D_ values are given in deg·cm·g^−1^·dm^−1^; concentration *c* is listed in g·(100 mL)^−1^. Analytical thin layer chromatography (TLC) was performed using pre-coated aluminum-backed plates (Merck Kieselgel 60 F254) and visualized by ultraviolet irradiation or Hanessian’s stain. The enantiomeric ratio (er) of the products was determined by chiral stationary phase UPC^2^ (Daicel Chiralpak IA column). Unless otherwise noted, analytical grade solvents and commercially available reagents were used without further purification. For flash chromatography (FC), silica gel (60, 35–70 μm, Merck KGaA, Darmstadt, Germany), 2-Nitrobenzofurans **1**, 2-nitro-benzo[*b*]thiophene **1r**, and imines **2** were obtained using literature procedures [58,59,60]. 

### 3.2. General Procedure for the Enantioselective Synthesis of ***3***

In an ordinary 4 mL glass vial equipped with a Teflon-coated magnetic stirring bar and screw cap, nitro-substituted benzoheteroarene **1** (1.0 equiv., 0.05 mmol), catalyst **4h** (0.2 equiv., 0.01 mmol, 6.3 mg), and the corresponding imine **2** (1.5 equiv., 0.075 mmol) were dissolved in freshly distilled CHCl_3_ (0.1 mL). The reaction mixture was stirred for the indicated time at ambient temperature. After full conversion of the starting material **1** (as confirmed by ^1^H NMR of a crude reaction mixture), the reaction mixture was directly subjected to flash chromatography on silica gel to obtain pure products **3**. The standard samples of products **3** for chiral UPC^2^ separation studies were prepared using equimolar mixture of quinine and quinidine as catalyst (See Supplementary Materials).

### 3.3. Procedure for the Enantioselective Synthesis of ***3a*** on a 1 mmol Scale

In an ordinary 12 mL glass vial equipped with a Teflon-coated magnetic stirring bar and screw cap, 2-nitrobenzofuran **1a** (1.0 equiv., 1.0 mmol, 163 mg), catalyst **4h** (0.2 equiv., 0.2 mmol, 126 mg), and corresponding imine **2a** (1.5 equiv., 0.15 mmol, 363 mg) were dissolved in freshly distilled CHCl_3_ (2 mL). The reaction mixture was stirred for 48 h at ambient temperature and was directly subjected to flash chromatography on silica gel (eluent: from hexanes/dichloromethane 1:1 to 100% dichloromethane) to obtain product **3a** as a single diastereoisomer (>20:1, 95:5 er) in 77% yield (312.1 mg).

### 3.4. Procudure for the Diastereoselective Synthesis of ***5***

To a stirred solution of **3a** (1.0 equiv., 0,128 mmol, 51 mg) in MeCN (1.5 mL), 1,8-diazabicyclo[5.4.0]undec-7-ene (DBU) (2.0 equiv., 0,256 mmol, 39 mg) was added. The reaction mixture was stirred for 72 h at room temperature and subsequently purified by flash chromatography on silica gel (eluent hexanes/ethyl acetate 4:1) to obtain product **5** as single diastereoisomer (>20:1) in 75% yield (34.4 mg).

### 3.5. Procudure for the Diastereoselective Synthesis of ***6***

To a stirred solution of **3a** (1.0 equiv., 0.05 mmol, 20.3 mg) in dry toluene (0.5 mL), tributyltin hydride (4.0 equiv., 0.2 mmol, 58 mg) and AIBN (2.0 equiv., 0.1 mmol, 16.4 mg) were added at room temperature. The reaction mixture was stirred for 3 h at 80 °C, cooled to room temperature, and CCl_4_ (0.15 mL) was added dropwise. After stirring for 5 min, saturated KF aq. solution (10 mL) was added and resulting mixture was extracted with AcOEt (3 × 10 mL). Combined organic layers were dried over Na_2_SO_4_, filtered, and concentrated under reduced pressure to obtain crude product, which was purified by flash chromatography on silica gel (eluent: hexanes/dichlorometane 1:1 to dichloromethane 100%) to obtain product **6** as single diastereoisomer (>20:1) in 68% yield (12.3 mg).

## 4. Conclusions

In conclusion, asymmetric dearomative (3+2)-cycloaddition between nitro-substituted benzoheteroarenes **1** and *N*-2,2,2-trifluoroethyl-substituted isatin imines **2** was developed. A squaramide-based cinchona alkaloid derivative efficiently promoted the reaction, ensuring high stereoselectivity of the process. Substrate specificity of the catalysts with regard to heteroaromatic framework was observed. Enantiomerically enriched products underwent chemoselective transformations that involved removal of the nitro group proceeding either with the concomitant aromatization of the heteroarene framework or in a non-aromative manner.

## Figures and Tables

**Table 1:**
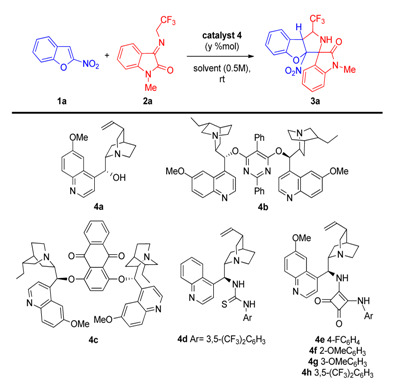
Asymmetric dearomative (3+2)-cycloaddition between nitro-substituted benzoheteroarenes **1** and *N*-2,2,2-trifluoroethyl-substituted isatin imines **2**–optimalization studies [a].

	Cat.	Solvent	Conv. (Yield) [%] [b,c]	dr [b]	er [d]
1	**4a**	CDCl_3_	41 (39)	4:1	50:50
2	**4b**	CDCl_3_	92 (88)	>20:1	43:57
3	**4c**	CDCl_3_	>95 (90)	>20:1	10:90
4	**4d**	CDCl_3_	>95 (88)	>20:1	52:48
5	**4e**	CDCl_3_	65 (58)	>20:1	95:5
6	**4f**	CDCl_3_	75 (69)	>20:1	95:5
7	**4g**	CDCl_3_	72 (68)	>20:1	90:10
8	**4h**	CDCl_3_	85 (78)	>20:1	96:4
9	**4h**	CH_2_Cl_2_	76 (73)	>20:1	93:7
10	**4h**	DCE	52	>20:1	n.d.
11	**4h**	AcOEt	51	>20:1	n.d.
12	**4h**	CH_3_CN	25	5:1	n.d.
13	**4h**	Et_2_O	49	>20:1	n.d.
14	**4h**	THF	34	>20:1	n.d.
15	**4h**	PhCH_3_	70	>20:1	n.d.
16 [e]	**4h**	CDCl_3_	73 (68)	>20:1	93:7
17 [f]	**4h**	CDCl_3_	>95 (89)	>20:1	96:4
18 [f,g]	**4h**	CDCl_3_	85 (79)	>20:1	95:5
19 [f,h]	**4h**	CDCl_3_	<10	n.d.	n.d.
20 [f,i]	**4h**	CHCl_3_	>95 (88)	>20:1	95.5:4.5
21 [f,j]	**4h**	CHCl_3_	>95 (77)	>20:1	95:5

[a] Reactions performed on a 0.05 mmol scale using **1a** (1.0 equiv.), **2a** (1.2 equiv.), and the catalyst **4** (20 mol%) in the corresponding solvent (0.1 mL) for 48 h at rt. [b] Determined by ^1^H NMR of a crude reaction mixture. [c] In parenthesis, isolated yields are given. [d] Determined by chiral UPC^2^ analysis. [e] Reaction performed in 0.2 mL of the solvent for 96 h. [f] Imine **2a** (1.5 equiv.) was used. [g] Reaction performed at 5 °C for 96 h. [h] Catalyst (5 mol%) was used. [i] Freshly distilled over P_2_O_5_ chloroform was used as a solvent. [j] Reaction performed on 1 mmol scale. DCE—1,2-dichloroethane.

## Data Availability

All the required data are reported in the manuscript and Supplementary Materials.

## Supplementary Materials

The following are available online. Characterization data for obtained products, X-ray data for product **3a**, copies of ^1^H and ^13^C NMR spectra, UPC^2^ plots for the cycloaddition products **3**.

## Sample Availability

Sample of the compound **3a** is available from the authors.
