# Supplementary material for: Asymmetric Dearomative (3+2)-Cycloaddition Involving Nitro-Substituted Benzoheteroarenes under H-Bonding Catalysis
Source: Molecules. 2021 Aug 18;26(16):4992. doi: 10.3390/molecules26164992 (PMC8401887; doi:10.3390/molecules26164992)
Supplement: Supplementary file 1 [file molecules-26-04992-s001.zip › molecules-1333782-supplementary.pdf]

Asymmetric dearomative (3+2)-cycloaddition involving nitro-substituted benzoheteroarenes under H-bonding catalysis

Maciej Saktura,[‡] Anna Skrzyńska,[‡,*] Sebastian Frankowski, Sylwia Wódka, and Łukasz Albrecht*

Institute of Organic Chemistry
Faculty of Chemistry,

Lodz University of Technology

Zeromskiego 116, 90-924 Łódź, Poland

e-mail: lukasz.albrecht@p.lodz.pl

anna.skrzynska@p.lodz.pl

http://www.a-teamlab.p.lodz.pl

Contents

1. General methods S2
2. Organocatalytic synthesis of **3** – general procedure S3
3. Enantioselective synthesis of (1*R*,3*S*,3a*S*,8b*S*)-1'-methyl-3a-nitro-1-(trifluoromethyl)-1,2,3a,8b-tetrahydrospiro[benzofuro[2,3-c]pyrrole-3,3'-indolin]-2'-one (**3a**) on a 1 mmol scale S13
4. Transformations of **3a** S14

4.1 Synthesis of (1*R*,3*S*)-1'-methyl-1-(trifluoromethyl)-1,2-dihydrospiro[benzofuro[2,3-c]pyrrole-3,3'-indolin]-2'-one (**5**) S14

4.2 Synthesis of (1*R*,3*S*,3a*S*,8b*S*)-1'-methyl-1-(trifluoromethyl)-1,2,3a,8b-tetrahydrospiro[benzofuro[2,3-c]pyrrole-3,3'-indolin]-2'-one (**6**) S14

5. Crystal and X-ray data for (1*R*,3*S*,3a*S*,8b*S*)-1'-methyl-3a-nitro-1-(trifluoromethyl)-1,2,3a,8b-tetrahydrospiro[benzofuro[2,3-c]pyrrole-3,3'-indolin]-2'-one (**3a**) S16
6. NMR data S18
7. UPC^2 traces S38

1. General methods

NMR spectra were acquired on a Bruker Ultra Shield 700 instrument, running at 700 MHz for ^{1}H and 176 MHz for ^{13}C, respectively. Chemical shifts (δ) are reported in ppm relative to residual solvent signals ($CDCl_3$: 7.26 ppm for ^{1}H NMR, 77.16 ppm for ^{13}C NMR). Mass spectra were recorded on a Bruker Maxis Impact spectrometer using electrospray (ES+) ionization referenced to the mass of the charged species. Optical rotations were measured on a Perkin-Elmer 241 polarimeter and $[\alpha]_D$ values are given in deg•cm•g^{-1}•dm^{-1}; concentration *c* is listed in g•(100 mL)$^{-1}$. Analytical thin layer chromatography (TLC) was performed using pre-coated aluminum-backed plates (Merck Kieselgel 60 F254) and visualized by ultraviolet irradiation or Hanessian's stain. The enantiomeric ratio (er) of the products was determined by chiral stationary phase UPC^2 (Daicel Chiralpak IA column). Unless otherwise noted, analytical grade solvents and commercially available reagents were used without further purification. For flash chromatography (FC) silica gel (60, 35-70 µm, Merck KGaA). 2-Nitrobenzofurans **1**, 2-nitro-benzo[*b*]thiophene **1r**, and imines **2** were obtained using literature procedures [1-3].

1. Lu, S.-C.; Zheng, P.-R.; Liu, G. Iodine(III)-mediated tandem oxidative cyclization for construction of 2-nitrobenzo[*b*]furans. *J. Org. Chem*. **2012**, *77*, 7711–7717.
2. Hayes, C.O.; Bell, W.K.; Cassidy, B.R.; Willson, C.G. Synthesis and characterization of a two stage, nonlinear photobase generator. *J. Org. Chem*. **2015**, *80*, 7530–7535.
3. Ma, M.; Zhu, Y.; Sun, Q.; Li, X.; Su, J.; Zhao, L.; Zhao, Y.; Qiu, S.; Yan, W.; Wang, K.; Wang, R. The asymmetric synthesis of CF3-containing spiro[pyrrolidin-3,2′-oxindole] through the organocatalytic 1,3-dipolar cycloaddition reaction. *Chem. Commun.* **2015**, *51*, 8789–8792.

2. Organocatalytic synthesis of 3 – general procedure

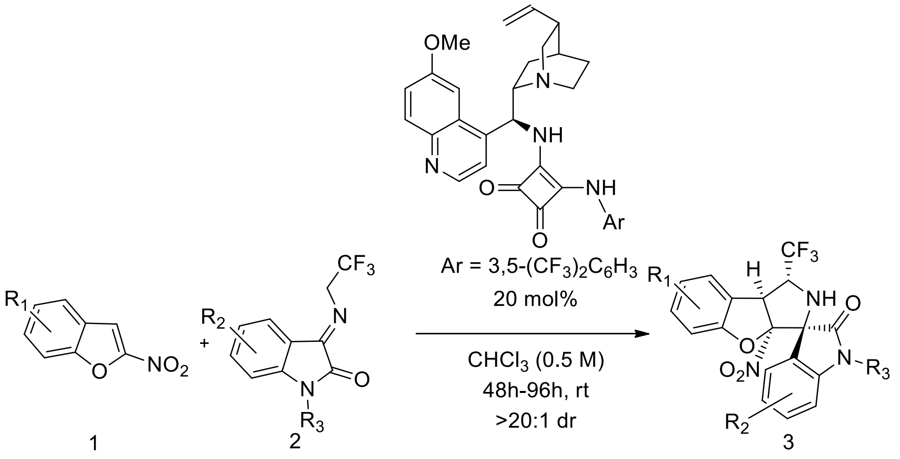

In an ordinary 4 mL glass vial, equipped with a Teflon-coated magnetic stirring bar and a screw cap, the corresponding nitro-substituted benzoheteroarene **1** (1.0 equiv., 0.05 mmol), catalyst **4h** (0.2 equiv., 0.02 mmol, 6.3 mg) and the corresponding imine **2** (1.5 equiv., 0.075 mmol) were dissolved in $CHCl_3$ (0.1 mL). The reaction mixture was stirred for indicated time at ambient temperature. After full conversion of the starting material **1** (as confirmed by ^{1}H NMR of a crude reaction mixture), the reaction mixture was directly subjected to flash chromatography on silica gel to obtain pure products **3**.

The racemic samples of products **3** for chiral UPC^2 separation studies were prepared using equimolar mixture of quinine and quinidine as catalyst.

(1*R*,3*S*,3a*S*,8b*S*)-1'-Methyl-3a-nitro-1-(trifluoromethyl)-1,2,3a,8b-tetrahydrospiro[benzofuro[2,3-c]pyrrole-3,3'-indolin]-2'-one (3a)

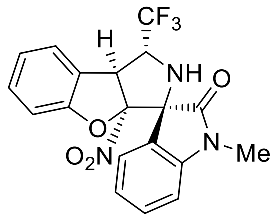

Following the general procedure product **3a** (reaction time: 2 days; >20:1 dr in a crude reaction mixture) was isolated after flash chromatography (eluent: hexanes/ dichlorometane 1:1 to dichloromethane 100%) in 88% (17.8 mg) yield as light-yellow oil. ^{1}H NMR (700MHz, $CDCl_3$) δ 7.47 – 7.40 (m, 2H), 7.35 – 7.29 (m, 2H), 7.17-7.14 (m, 1H), 7.12-7.09 (m, 1H), 7.00-6.98 (m, 1H), 6.92 (d, *J* = 7.8 Hz, 1H), 5.25 (d, *J* = 7.2 Hz, 1H), 4.07-4.01 (m, 1H), 3.21 (s, 3H), 2.88 (d, *J* = 9.1 Hz, 1H). ^{13}C NMR (176 MHz, $CDCl_3$) δ 173.2, 156.6, 144.8, 131.3, 130.4, 126.6, 126.2, 125.9, 125.1, 125.0, 124.8 (q, *J* = 281.1 Hz), 124.5, 123.3, 111.1, 109.3, 71.72, 66.0 (q, *J* = 32.5 Hz), 51.9 (q, *J* = 2.5 Hz), 26.8. The er was determined by UPC^2 using a chiral Chiralpack IA column gradient from 100% CO_2 up to 40%; *i*-PrOH, 2.5 mL/min; τ_{major} = 2.84 min, τ_{minor} = 3.19 min, (95.5:4.5 er). $[\alpha]_D^{24}$ = -150.4 (c= 0.52, $CHCl_3$). HRMS calculated for $[C_{19}H_{14}F_3N_3O_4+Na^+]$: 428.0829; found: 428.0834.

(1*R*,3*S*,3a*S*,8b*S*)-1'-Allyl-3a-nitro-1-(trifluoromethyl)-1,2,3a,8b-tetrahydrospiro[benzofuro[2,3-c]pyrrole-3,3'-indolin]-2'-one (3b)

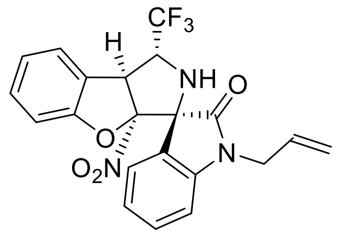

Following the general procedure product **3b** (reaction time: 2 days; >20:1 dr in a crude reaction mixture) was isolated after flash chromatography (eluent: hexanes/dichlorometane 1:1 to dichloromethane 100%) in 90% yield (19.4 mg) as light-yellow oil. ^{1}H NMR (700MHz, $CDCl_3$) δ 7.45 – 7.38 (m, 2H), 7.36 – 7.31 (m, 2H), 7.17 – 7.15 (m, 1H), 7.11 – 7.09 (m, 1H), 7.02 – 6.98 (m, 1H), 6.92 – 6.91 (m, 1H), 5.81 (dddd, *J*= 17.2, 10.5, 5.6, 5.0 Hz, 1H), 5.35 – 5.22 (m, 3H), 4.44 (ddt, *J*= 16.4, 5.0, 1.7 Hz, 1H), 4.19 (ddt, *J*= 16.4, 5.6, 1.6 Hz, 1H), 4.07 – 4.02 (m, 1H), 2.88 (d, *J*= 9.1 Hz, 1H). ^{13}C NMR (176 MHz, CDCl3) δ 173.1, 156.5, 144.0, 131.2, 130.6, 130.4, 126.7, 126.2, 126.1, 125.2, 125.0, 124.8 (q, *J*= 280.4 Hz) 124.6, 123.2, 118.4, 111.1, 110.2, 71.6, 66.1 (q, *J*= 66.1 Hz), 51.9 (q, *J*= 2.1 Hz), 42.9. The er was determined by UPC^2 using a chiral Chiralpack IA column gradient from 100% CO_2 up to 40%; *i*-PrOH, 2.5 mL/min; τ_{major} = 2.99 min, τ_{minor} = 3.37 min, (94:6 er). $[\alpha]_D^{24}$ = -118.6 (c= 0.4, $CHCl_3$). HRMS calculated for $[C_{21}H_{16}F_3N_3O_4+Na^+]$: 454.0985; found: 454.0990.

(1*R*,3*S*,3a*S*,8b*S*)-1'-Benzyl-3a-nitro-1-(trifluoromethyl)-1,2,3a,8b-tetrahydrospiro[benzofuro[2,3-c]pyrrole-3,3'-indolin]-2'-one (3c)

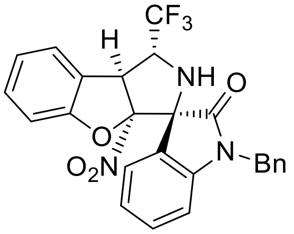

Following the general procedure product **3c** (reaction time: 6 days; >20:1 dr in a crude reaction mixture) was isolated after flash chromatography (eluent: hexanes/dichlorometane 1:1 to dichloromethane 100%) in 92% yield (22.1 mg) as light-yellow oil. ^{1}H NMR (700 MHz, $CDCl_3$) δ 7.44 (d, *J*= 7.5 Hz, 1H), 7.36–7.26 (m, 8H), 7.18–7.15 (m, 1H), 7.07-7.05 (m, 1H), 7.00 (dd, *J*= 8.0, 0.7 Hz, 1H), 6.76 (dd, *J*= 7.9, 0.7 Hz, 1H), 5.30 (d, *J*= 7.2 Hz, 1H), 5.05 (d, *J*= 15.8, 1H), 4.73 (d, *J*= 15.8 Hz, 1H), 4.10-4.04 (m, 1H), 2.93 (d, J = 9.1 Hz, 1H). ^{13}C NMR (176 MHz, $CDCl_3$) δ 173.4, 156.6, 143.9, 135.0, 131.1, 130.4, 129.0 (2C), 128.0, 127.5 (2C), 126.6, 126.2, 126.0, 125.3, 125.0, 124.8 (q. *J*= 279.8 Hz), 124.5, 123.3, 111.1, 110.4, 71.8, 66.1, (q, *J*= 32.4 Hz), 52.0, 44.5. The er was determined by UPC^2 using a chiral Chiralpack IA column gradient from 100% CO_2 up to 40%; *i*-PrOH, 2.5 mL/min; τ_{major} = 3.48 min, τ_{minor} = 3.79 min, (93:7 er). $[\alpha]_D^{24}$ = -80.6 (c= 1.0, $CHCl_3$). HRMS calculated for $[C_{25}H_{18}F_3N_3O_4+Na^+]$: 504.1142; found: 504.1144.

(1*R*,3*S*,3a*S*,8b*S*)-3a-Nitro-1-(trifluoromethyl)-1,2,3a,8b-tetrahydrospiro[benzofuro[2,3-c]pyrrole-3,3'-indolin]-2'-one (3d)

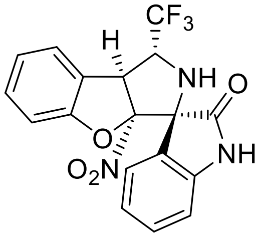

Following the general procedure product **3d (**reaction time: 4 days; >20:1 dr in a crude reaction mixture) was isolated after flash chromatography (eluent: dichloromethane 100% to dichloromethane/ethyl acetate 98:2) in 87% yield (17.0 mg) as light-yellow oil. ^{1}H NMR (700MHz, $CDCl_3$) δ 7.86 (bs, 1H), 7.43 (d, *J* = 7.5 Hz, 1H), 7.39-7.36 (m, 1H), 7.36 – 7.30 (m, 2H), 7.17-7.16 (m, 1H), 7.10-7.07 (m, 1H), 7.01 (dd, *J* = 8.1, 0.8 Hz, 1H), 6.97-6.95 (m, 1H), 5.23 (d, *J* = 7.1 Hz, 1H), 4.07-4.01 (m, 1H), 2.88 (d, *J* = 8.8 Hz, 1H). ^{13}C NMR (176 MHz, $CDCl_3$) δ 174.8, 156.5, 141.7, 131.3, 130.5, 126.7, 126.4, 126.0, 125.6, 125.0, 124.8 (q, *J* = 280.9 Hz), 124.6, 123.3, 111.1, 111.0, 71.9, 66.0 (q, *J* = 32.5 Hz), 51.8 (q, *J* = 2.3 Hz). The er was determined by UPC^2 using a chiral Chiralpack IA column gradient from 100% CO_2 up to 40%; *i*-PrOH, 2.5 mL/min; τ_{major} = 3.71 min, τ_{minor} = 4.03 min, (96:4 er). $[\alpha]_D^{24}$ = -128.5 (c= 1.0, $CHCl_3$). HRMS calculated for $[C_{18}H_{12}F_3N_3O_4+Na^+]$: 414.0672; found: 414.0679.

(1*R*,3*S*,3a*S*,8b*S*)-5'-Methoxy-3a-nitro-1-(trifluoromethyl)-1,2,3a,8b-tetrahydrospiro[benzofuro[2,3-c]pyrrole-3,3'-indolin]-2'-one (3e)

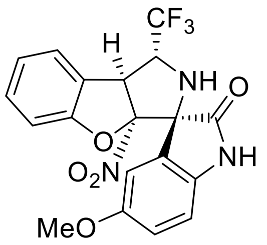

Following the general procedure product **3e** (reaction time: 2 days; >20:1 dr in a crude reaction mixture) was isolated after flash chromatography (eluent: hexanes/ dichloromethane 1:1 to dichloromethane 100%) in 70% yield (14.7 mg) as light-yellow oil. ^{1}H NMR (700MHz, $CDCl_3$) δ 7.73 (bs, 1H), 7.42 (d, *J* = 7.5 Hz, 1H), 7.35-7.32 (m, 1H), 7.17-7.14 (m, 1H), 7.01 (d, *J* = 8.1 Hz, 1H), 6.92 – 6.85 (m, 3H), 5.23 (d, *J* = 7.1 Hz, 1H), 4.06-4.00 (m, 1H), 3.74 (s, 3H), 2.87 (d, *J* = 8.8 Hz, 1H). ^{13}C NMR (176 MHz, $CDCl_3$) δ 174.7, 156.6, 156.2, 134.9, 130.5, 126.7, 126.6, 125.9,125.0, 124.7 (q, *J* = 281.7 Hz), 124.58, 115.6, 113.8, 111.4, 111.1, 72.3, 66.0 (q, *J* = 31.7 Hz), 56.0, 51.8 (q, *J* = 2.3 Hz). The er was determined by UPC^2 using a chiral Chiralpack IA column gradient from 100% CO_2 up to 40%; *i*-PrOH, 2.5 mL/min; τ_{major} = 3.68 min, τ_{minor} = 4.16 min, (96:4 er). $[\alpha]_D^{24}$ = -131.5 (c= 0.4, $CHCl_3$). HRMS calculated for [$C_{19}H_{14}F_3N_3O_5$+Na^+]: 444.0778; found: 444.0782.

(1*R*,3*S*,3a*S*,8b*S*)-5'-Methyl-3a-nitro-1-(trifluoromethyl)-1,2,3a,8b-tetrahydrospiro[benzofuro[2,3-c]pyrrole-3,3'-indolin]-2'-one (3f)

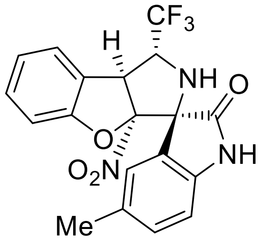

Following the general procedure product **3f** (reaction time: 2 days; >20:1 dr in a crude reaction mixture) was isolated after flash chromatography (eluent: hexanes/ dichloromethane 1:1 to dichloromethane 100%) in 82% yield (16.6 mg) as light-yellow oil. ^{1}H NMR (700MHz, $CDCl_3$) δ 7.77 (bs, 1H), 7.43 (d, *J*= 7.5 Hz, 1H), 7.35 – 7.33 (m, 1H), 7.18 – 7.15 (m, 2H), 7.11 – 7.10 (m, 1H), 7.023 – 7.02 (m, 1H), 6.85 (d, *J*= 7.9 Hz, 1H), 5.23 (d, *J*= 7.1 Hz, 1H), 4.06 – 4.01 (m, 1H), 2.86 (d, *J*= 8.8 Hz, 1H), 2.31 (s, 3H).^{13}C NMR (176 MHz, $CDCl_3$) δ 174.9, 156.6, 139.2, 132.9, 131.7, 130.4, 127.0, 126.8, 126.0, 125.5, 125.0, 124.8 (q, *J*= 280.1 Hz), 124.5, 111.1, 110.7, 72.0, 66.0 (q, *J*= 32.6 Hz), 51.8, 21.3. The er was determined by UPC^2 using a chiral Chiralpack IA column gradient from 100% CO_2 up to 40%; *i*-PrOH, 2.5 mL/min; τ_{major} = 3.55 min, τ_{minor} = 3.95 min, (95.5:4.5 er). $[\alpha]_D^{19}$ = -233.1 (c= 0.5, $CHCl_3$). HRMS calculated for [$C_{19}H_{14}F_3N_3O_4$+Na^+]: 428.0829; found: 428.0832.

(1*R*,3*S*,3a*S*,8b*S*)-5'-Bromo-3a-nitro-1-(trifluoromethyl)-1,2,3a,8b-tetrahydrospiro[benzofuro[2,3-c]pyrrole-3,3'-indolin]-2'-one (3g)

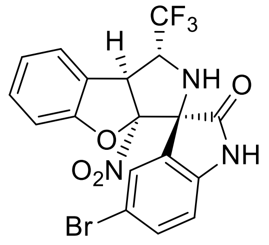

Following the general procedure product **3g** (reaction time: 4 days; >20:1 dr in a crude reaction mixture) was isolated after flash chromatography (eluent: dichloromethane 100% to dichlorometane/ethyl acetate 98:2) in 74% yield (17.4 mg) as light-yellow oil. ^{1}H NMR (700MHz, $CDCl_3$) δ 7.62 (bs, 1H), 7.43 (d, *J* = 7.5 Hz, 1H), 7.38 (dd, *J* = 8.2, 0.9 Hz, 1H), 7.34 (td, *J* = 7.9, 1.2 Hz, 1H), 7.25 (d, *J* = 7.5 Hz, 1H), 7.17 (td, *J* = 7.5, 0.8 Hz, 1H), 7.07 – 7.03 (m, 1H), 7.01 (d, *J* = 8.0 Hz, 1H), 5.21 (d, *J* = 7.0 Hz, 1H), 4.03 (dt, *J* = 8.5, 7.0 Hz, 1H), 2.87 (d, *J* = 8.6 Hz, 1H). ^{13}C NMR (176 MHz, $CDCl_3$) δ 173.6, 156.4, 139.6, 131.2, 130.5, 126.8, 126.6, 125.8, 125.0, 124.7, 124.7, 124.7 (q, *J* = 280.2 Hz), 124.1, 116.2, 111.1, 72.6, 66.2 (q, *J* = 32.2 Hz), 51.7. The er was determined by UPC^2 using a chiral Chiralpack IA column gradient from 100% CO_2 up to 40%; *i*-PrOH, 2.5 mL/min; τ_{major} = 3.66 min, τ_{minor} = 3.98 min, (95:5 er). $[\alpha]_D^{24}$ = -138.9 (c= 0.4, $CHCl_3$). HRMS calculated for $[C_{18}H_{11}BrF_3N_3O_4+Na^+]$: 491.9777; found: 491.9773.

(1*R*,3*S*,3a*S*,8b*S*)-5'-Chloro-3a-nitro-1-(trifluoromethyl)-1,2,3a,8b-tetrahydrospiro[benzofuro[2,3-c]pyrrole-3,3'-indolin]-2'-one (3h)

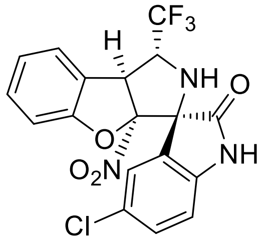

Following the general procedure product **3h** (reaction time: 4 days; >20:1 dr in a crude reaction mixture) was isolated after flash chromatography (eluent: dichloromethane 100% to dichlorometane/ethyl acetate 98:2) in 77% yield (16.4 mg) as light-yellow oil. ^{1}H NMR (700MHz, $CDCl_3$) δ 8.10 (bs, 1H), 7.43 (d, *J* = 7.5 Hz, 1H), 7.39 – 7.32 (m, 3H), 7.18 (td, *J* = 7.5, 0.9 Hz, 1H), 7.07 (dd, *J* = 8.0, 0.8 Hz, 1H), 6.91 (dd, *J* = 8.3, 0.5 Hz, 1H), 5.20 (d, *J* = 7.0 Hz, 1H), 4.02 (dp, *J* = 8.4, 6.9 Hz, 1H), 2.87 (d, *J* = 8.4 Hz, 1H).^{13}C NMR (176 MHz, $CDCl_3$) δ 174.9, 156.3, 140.4, 131.3, 130.6, 128.7, 127.0, 126.9, 126.7, 125.6, 124.9, 124.8, 124.7 (q, *J* = 280.2 Hz), 112.1, 111.3, 71.7, 66.1 (q, *J* = 32.2 Hz), 51.5. The er was determined by UPC^2 using a chiral Chiralpack IA column gradient from 100% CO_2 up to 40%; *i*-PrOH, 2.5 mL/min; τ_{major} = 3.52 min, τ_{minor} = 3.81 min, (95:5 er). $[\alpha]_D^{24}$ = -125.2 (c= 0.9, $CHCl_3$). HRMS calculated for $[C_{18}H_{11}ClF_3N_3O_4+Na^+]$: 448.0282; found: 448.0287.

(1*R*,3*S*,3a*S*,8b*S*)-7'-Chloro-3a-nitro-1-(trifluoromethyl)-1,2,3a,8b-tetrahydrospiro[benzofuro[2,3-c]pyrrole-3,3'-indolin]-2'-one (3i)

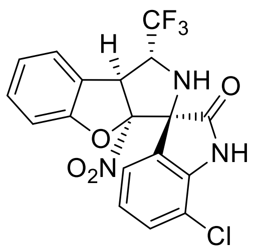

Following the general procedure product **3i** (reaction time: 4 days; >20:1 dr in a crude reaction mixture) was isolated after flash chromatography (eluent: dichloromethane 100% to dichlorometane/ethyl acetate 98:2) in 83% yield (17.6 mg) as light-yellow oil. ^{1}H NMR (700MHz, $CDCl_3$) δ 7.59 (bs, 1H), 7.43 (d, *J* = 7.5 Hz, 1H), 7.38 (dd, *J* = 8.2, 0.9 Hz, 1H), 7.34 (td, *J* = 8.0, 1.3 Hz, 1H), 7.25 (d, *J* = 7.5 Hz, 1H), 7.17 (td, *J* = 7.5, 0.7 Hz, 1H), 7.07 – 7.04 (m, 1H), 7.01 (d, *J* = 8.1 Hz, 1H), 5.21 (d, *J* = 7.0 Hz, 1H), 4.08 – 3.98 (m, 1H), 2.87 (d, *J* = 8.6 Hz, 1H). ^{13}C NMR (176 MHz, $CDCl_3$) δ 173.6, 156.4, 139.6, 131.2, 130.5, 126.8, 126.6, 125.8, 125.0, 124.7(q, *J* = 280.2 Hz), 124.7, 124.7, 124.1, 116.2, 111.1, 72.6, 66.2 (q, *J* = 32.5 Hz), 51.7. The er was determined by UPC^2 using a chiral Chiralpack IA column gradient from 100% CO_2 up to 40%; *i*-PrOH, 2.5 mL/min; τ_{major} = 3.66 min, τ_{minor} = 3.99 min, (94:6 er). $[\alpha]_D^{24}$ = -147.9 (c= 0.5, $CHCl_3$). HRMS calculated for $[C_{18}H_{11}ClF_3N_3O_4+Na^+]$: 448.0282; found: 448.0281.

(1*R*,3*S*,3a*S*,8b*S*)-3a,5'-Dinitro-1-(trifluoromethyl)-1,2,3a,8b-tetrahydrospiro[benzofuro[2,3-c]pyrrole-3,3'-indolin]-2'-one (3j)

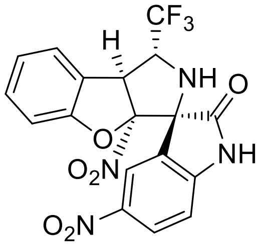

Following the general procedure product **3j** reaction time: 4 days; >20:1 dr in a crude reaction mixture) was isolated (eluent: dichloromethane 100% to dichloromethane/ethyl acetate 98:2) in 77% yield (16.8 mg) as light-yellow oil. ^{1}H NMR (700MHz, $CDCl_3$) δ δ 7.93 (bs, 1H), 7.52 (dd, *J* = 8.3, 2.0 Hz, 1H), 7.48 (t, *J* = 2.4 Hz, 1H), 7.43 (d, *J* = 7.4 Hz, 1H), 7.37 (ddd, *J* = 9.1, 6.5, 2.6 Hz, 1H), 7.19 – 7.16 (m, 1H), 7.07 (d, *J* = 8.0 Hz, 1H), 6.86 (d, *J* = 8.3 Hz, 1H), 5.20 (d, *J* = 7.0 Hz, 1H), 4.06 – 3.98 (m, 1H), 2.85 (d, *J* = 8.4 Hz, 1H). ^{13}C NMR (176 MHz, $CDCl_3$) δ 174.8, 156.3, 140.9, 134.2, 130.6, 129.8, 127.3, 126.7, 125.6, 124.9, 124.8, 124.7 (q, *J* = 279.9 Hz), 115.8, 112.5, 111.29, 71.6, 66.1 (q, *J* = 32.4 Hz), 51.5. The er was determined by UPC^2 using a chiral Chiralpack IA column gradient from 100% CO_2 up to 40%; *i*-PrOH, 2.5 mL/min; τ_{major} = 3.62 min, τ_{minor} = 3.94 min, (96:4 er). $[\alpha]_D^{24}$ = -147.7 (c= 0.6, $CHCl_3$). HRMS calculated for $[C_{18}H_{11}F_3N_4O_6+Na^+]$: 459.0523; found: 459.0528

(1*R*,3*S*,3a*S*,8b*S*)-5',7'-Dibromo-3a-nitro-1-(trifluoromethyl)-1,2,3a,8b-tetrahydrospiro[benzofuro[2,3-c]pyrrole-3,3'-indolin]-2'-one (3k)

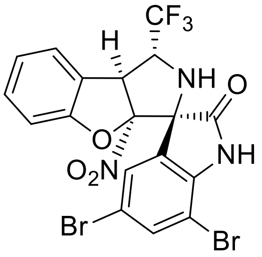

Following the general procedure product **3k** (reaction time: 4 days; >20:1 dr in a crude reaction mixture) was isolated after flash chromatography (eluent: hexanes/dichloromethane 1:1 to dichloromethane 100%) in 76% yield (16.5 mg) as a light-yellow oil. ^{1}H NMR (700MHz, $CDCl_3$) δ 7.82 (s, 1H), 7.69 (d, *J* = 1.7 Hz, 1H), 7.45 (d, *J* = 1.7 Hz, 1H), 7.42 (t, *J* = 8.0 Hz, 1H), 7.37 (ddd, *J* = 9.0, 6.8, 1.4 Hz, 1H), 7.18 (td, *J* = 7.5, 0.8 Hz, 1H), 7.07 (d, *J* = 8.1 Hz, 1H), 5.17 (d, *J* = 6.9 Hz, 1H), 4.01 (dp, *J* = 14.0, 7.0 Hz, 1H), 2.86 (d, *J* = 8.2 Hz, 1H). ^{13}C NMR (176 MHz, $CDCl_3$) δ 173.2, 156.2, 140.5, 136.2, 130.6, 128.8, 127.9, 126.6, 125.5, 125.0, 124.6 (q, *J* = 280.4 Hz), 124.9, 115.9, 111.3, 104.4, 72.6, 66.2 (q, *J* = 32.4 Hz), 51.4. The er was determined by UPC^2 using a chiral Chiralpack IA column gradient from 100% CO_2 up to 40%; *i*-PrOH, 2.5 mL/min; τ_{major} = 3.64 min, τ_{minor} = 3.84 min, (89:11 er). $[\alpha]_D^{24}$ = -102.6 (c = 0.6, $CHCl_3$). HRMS calculated for [$C_{18}H_{10}Br_2F_3N_3O_4+K^+$]: 585.8622; found: 585.8644.

(1*R*,3*S*,3a*S*,8b*S*)-7-Methoxy-1'-methyl-3a-nitro-1-(trifluoromethyl)-1,2,3a,8b-tetrahydrospiro[benzofuro[2,3-c]pyrrole-3,3'-indolin]-2'-one (3l)

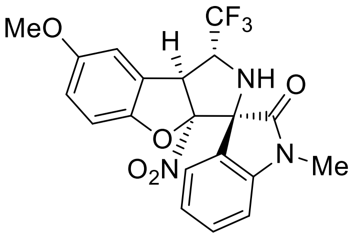

Following the general procedure product **3l** (reaction time: 2 days; >20:1 dr in a crude reaction mixture) was isolated after flash chromatography (eluent: hexanes/dichloromethane 1:1 to dichloromethane 100%) in 76% yield (16.5 mg) as a light-yellow oil.^{1}H NMR (700MHz, $CDCl_3$) δ 7.45 – 7.41 (m, 1H), 7.30 – 7.27 (m, 2H), 7.12 – 7.08 (m, 1H), 6.92 (d, *J* = 7.8 Hz, 1H), 6.67 (dd, *J* = 8.4, 2.3 Hz, 1H), 6.54 (d, *J* = 2.3 Hz, 1H), 5.18 (d, *J* = 7.2 Hz, 1H), 4.02 (dp, *J* = 9.1, 6.9 Hz, 1H), 3.79 (s, 3H), 3.20 (s, 3H), 2.88 (t, *J* = 10.3 Hz, 1H). ^{13}C NMR (176 MHz, $CDCl_3$) δ 173.1, 162.0, 157.9, 144.7, 131.3, 127.0, 125.9, 125.4, 125.2, 124.9 (q, *J* = 280.1 Hz), 123.3, 118.2, 110.1, 109.3, 97.8, 71.9, 66.4 (q, *J* = 31.8 Hz), 55.9, 51.6, 26.8. The er was determined by UPC^2 using a chiral Chiralpack IA column gradient from 100% CO_2 up to 40%; i-PrOH, 2.5 mL/min; τ_{major} = 3.20 min, τ_{minor} = 3.48 min, (93:7 er). $[\alpha]_D^{24}$ = -97.8 (c = 0.6, $CHCl_3$). HRMS calculated for [$C_{20}H_{16}F_3N_3O_5+Na^+$]: 458.0934; found: 458.0936

(1*R*,3*S*,3a*S*,8b*S*)-1',7-Dimethyl-3a-nitro-1-(trifluoromethyl)-1,2,3a,8b-tetrahydrospiro[benzofuro[2,3-c]pyrrole-3,3'-indolin]-2'-one (3m)

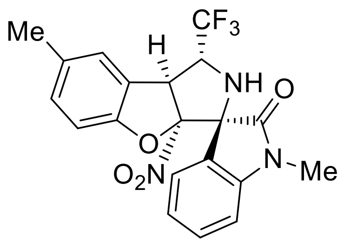

Following the general procedure product **3m** (reaction time: 2 days; >20:1 dr in a crude reaction mixture) was isolated isolated after flash chromatography (eluent: hexanes/dichloromethane 1:1 to dichloromethane 100%) in 73% yield (15.3 mg) as a light-yellow oil. ^{1}H NMR (700MHz, $CDCl_3$) δ 7.45 – 7.42 (m, 1H), 7.32 (dd, *J* = 7.5, 0.7 Hz, 1H), 7.21 (s, 1H), 7.11 (ddd, *J* = 8.5, 4.8, 0.8 Hz, 2H), 6.91 (d, *J* = 7.8 Hz, 1H), 6.86 (d, *J* = 8.2 Hz, 1H), 5.20 (d, *J* = 7.2 Hz, 1H), 4.09 – 3.95 (m, 1H), 3.20 (s, 3H), 2.88 (d, *J* = 9.2 Hz, 1H), 2.37 (s, 3H). ^{13}C NMR (176 MHz, $CDCl_3$) δ 173.27, 154.57, 144.78, 134.34, 131.26, 130.76, 126.59, 126.34, 125.99, 125.39, 125.19, 124.9 (q, *J* = 280.2 Hz), 123.25, 110.65, 109.21, 71.67, 51.94, 66.0 (q, *J* = 32.1 Hz), 26.78, 21.08. The er was determined by UPC^2 using a chiral Chiralpack IA column gradient from 100% CO_2 up to 40%; *i*-PrOH, 2.5 mL/min; τ_{major} = 3.08 min, τ_{minor} = 3.38 min, (95:5 er). $[\alpha]_D^{21}$ = -129.6 (c = 0.6, $CHCl_3$). HRMS calculated for $[C_{20}H_{16}F_3N_3O_4+Na^+]$: 442.0985; found: 442.0991.

(1*R*,3*S*,3a*S*,8b*S*)-7-(*tert*-Butyl)-1'-methyl-3a-nitro-1-(trifluoromethyl)-1,2,3a,8b-tetrahydrospiro[benzofuro[2,3-c]pyrrole-3,3'-indolin]-2'-one (3n)

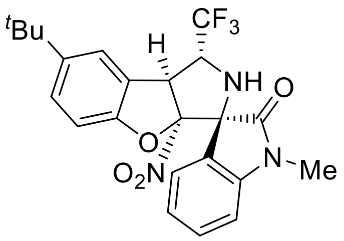

Following the general procedure product **3n** (reaction time: 2 days; >20:1 dr in a crude reaction mixture) was isolated after flash chromatography (eluent: hexanes/dichloromethane 1:1 to dichloromethane 100%) in 95% yield (21.9 mg) as a light-yellow oil. ^{1}H NMR (700MHz, $CDCl_3$) δ 7.46 – 7.40 (m, 2H), 7.34 (ddd, *J*= 7.5, 3.8, 1.6 Hz, 2H), 7.12 - 7.10 (m 1H), 6.92 – 6.91 (m, 2H), 5.23 (d, *J*= 7.2 Hz, 1H), 4.08 – 4.02 (m, 1H), 3.19 (s, 3H), 2.87 (d, *J*= 9.1 Hz, 1H), 1.34 (s, 9H).^{13}C NMR (176 MHz, $CDCl_3$) δ 173.4, 154.3, 148.1, 144.8, 131.3, 127.3, 126.4, 126.3, 126.0, 125.2, 124.9 (q, *J*= 280.4 Hz), 123.3, 121.8, 110.3, 109.2, 71.6, 66.0 (q, *J*= 32.0 Hz), 52.0, 34.9, 31.7 (3C), 26.8. The er was determined by UPC^2 using a chiral Chiralpack IA column gradient from 100% CO_2 up to 40%; *i*-PrOH, 2.5 mL/min; τ_{major} = 3.03 min, τ_{minor} = 3.22 min, (95:5 er). $[\alpha]_D^{24}$ = -136.1 (c = 1.0, $CHCl_3$). HRMS calculated for $[C_{23}H_{22}F_3N_3O_4+Na^+]$: 484.1455; found: 484.1461.

(1*R*,3*S*,3a*S*,8b*S*)-7-Chloro-1'-methyl-3a-nitro-1-(trifluoromethyl)-1,2,3a,8b-tetrahydrospiro[benzofuro[2,3-c]pyrrole-3,3'-indolin]-2'-one (3o)

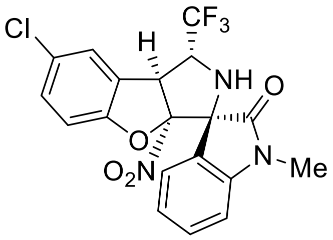

Following the general procedure product **3o** (reaction time: 2 days; >20:1 dr in a crude reaction mixture) was isolated after flash chromatography (eluent: hexanes/dichloromethane 1:1 to dichloromethane 100%) in 80% yield (17.6 mg) as a light-yellow oil. ^{1}H NMR (700MHz, $CDCl_3$) δ 7.47 – 7.43 (m, 1H), 7.40 (d, *J* = 2.1 Hz, 1H), 7.32 – 7.29 (m, 2H), 7.13 – 7.09 (m, 1H), 6.93 (dt, *J* = 7.8, 4.6 Hz, 2H), 5.22 (d, *J* = 7.2 Hz, 1H), 4.05 (dp, *J* = 9.0, 6.9 Hz, 1H), 3.20 (s, 3H), 2.90 (d, *J* = 9.1 Hz, 1H). ^{13}C NMR (176 MHz, $CDCl_3$) δ 172.89, 154.95, 144.65, 131.29, 130.32, 129.59, 128.36, 126.24, 125.71, 125.10, 124.64, 124.5 (q, *J* = 280.1 Hz), 123.16, 112.01, 109.19, 71.44, 65.7 (q, *J* = 32.4 Hz), 51.65, 26.66. The er was determined by UPC^2 using a chiral Chiralpack IA column gradient from 100% CO_2 up to 40%; *i*-PrOH, 2.5 mL/min; τ_{major} = 3.21 min, τ_{minor} = 3.57 min, (93:7 er). $[\alpha]_D^{24}$ = -95.5 (c = 1.0, $CHCl_3$). HRMS calculated for [$C_{19}H_{13}ClF_3N_3O_4$+K^+]: 478.0178; found: 478.0185.

(1*R*,3*S*,3a*S*,8b*S*)-1'-Methyl-3a,7-dinitro-1-(trifluoromethyl)-1,2,3a,8b-tetrahydrospiro[benzofuro[2,3-c]pyrrole-3,3'-indolin]-2'-one (3p)

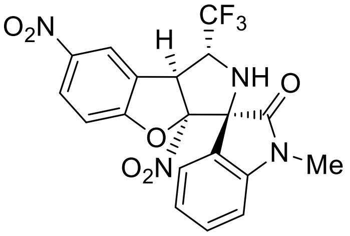

Following the general procedure product **3p** (reaction time: 2 days; >20:1 dr in a crude reaction mixture) was isolated after flash chromatography (eluent: hexanes/dichloromethane 1:1 to dichloromethane 100%) in 81% yield (18. 2 mg) as a light-yellow oil.^{1}H NMR (700 MHz, Acetone-*d6*) δ 8.41 – 8.36 (m, 2H), 7.59 (ddd, *J*= 7.5, 1.3, 0.6 Hz, 1H), 7.51-7.49 (m, 1H), 7.40-7.39 (m, 1H), 7.17 – 7.10 (m, 2H), 5.43 (d, *J*= 6.8 Hz, 1H), 4.47 – 4.41 (m, 1H), 4.36 (d, *J*= 9.0 Hz, 1H), 3.20 (s, 3H).^{13}C NMR (176 MHz, Acetone-*d6*) δ 174.4, 161.8, 146.1, 145.9, 132.2, 130.5, 128.4, 128.1, 127.7, 126.3 (q, *J*= 279.6 Hz), 125.4, 123.8, 122.1, 112.6, 110.2, 72.5, 66.2 (q, *J*= 32.3 Hz), 52.0, 26.9.The er was determined by UPC^2 using a chiral Chiralpack IA column gradient from 100% CO_2 up to 40%; *i*-PrOH, 2.5 mL/min; τ_{major} = 3.52 min, τ_{minor} = 3.79 min, (95.5:4.5 er) $[\alpha]_D^{20}$ = -150.9 (c = 0.6, acetone). HRMS calculated for [$C_{19}H_{13}F_3N_4O_6$+K^+]: 489.0419; found: 489.0423.

(1*R*,3*S*,3a*S*,8b*S*)-6-Bromo-1'-methyl-3a-nitro-1-(trifluoromethyl)-1,2,3a,8b-tetrahydrospiro[benzofuro[2,3-c]pyrrole-3,3'-indolin]-2'-one (3q)

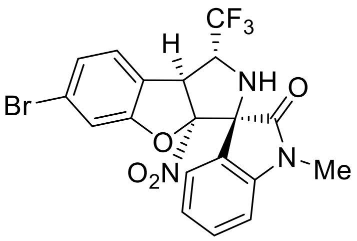

Following the general procedure product **3q** (reaction time: 2 days; >20:1 dr in a crude reaction mixture) was isolated after flash chromatography (eluent: hexanes/dichlorometane 1:1 to dichloromethane 100%) in 82% yield (19.9 mg) as a light-yellow oil. ^{1}H NMR (700MHz, $CDCl_3$) δ 7.48 – 7.43 (m, 1H), 7.32 – 7.28 (m, 3H), 7.18 (d, *J* = 1.5 Hz, 1H), 7.14 – 7.10 (m, 1H), 6.97 – 6.89 (m, 1H), 5.19 (d, *J* = 7.2 Hz, 1H), 4.06 – 3.98 (m, 1H), 3.20 (d, *J* = 1.3 Hz, 3H), 2.89 (d, *J* = 9.1 Hz, 1H). ^{13}C NMR (176 MHz, $CDCl_3$) δ 173.1, 157.2, 144.8, 131.5, 127.8, 126.4, 126.0, 126.0, 125.9, 124.8, 124.7 (q, *J*= 280.2 Hz), 123.5, 123.4, 114.9, 109.4, 71.6, 65.9 (q, *J* = 32.3 Hz), 51.5, 26.8. The er was determined by UPC^2 using a chiral Chiralpack IA column gradient from 100% CO_2 up to 40%; *i*-PrOH, 2.5 mL/min; τ_{major} = 3.29 min, τ_{minor} = 3.59 min, (94:6 er). $[\alpha]_D^{24}$ = -124.5 (c = 0.7, $CHCl_3$). HRMS calculated for $[C_{19}H_{13}BrF_3N_3O_4+Na^+]$: 505.9934; found: 505.9930.

(1*R*,3*R*,3a*S*,8b*S*)-1'-Methyl-3a-nitro-1-(trifluoromethyl)-1,2,3a,8b-tetrahydrospiro[benzo[4,5]thieno[2,3-c]pyrrole-3,3'-indolin]-2'-one (3r)

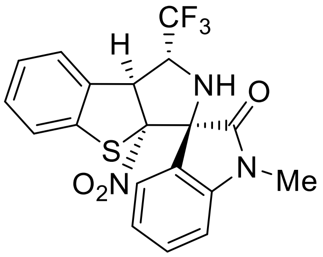

Following the modified general procedure product **3r** (catalyst **4d** instead of **4h** was used. Reaction time: 2 days; >20:1 dr in a crude reaction mixture) was isolated after flash chromatography (eluent: hexanes/dichlorometane 1:1 to 3:2) in 81% yield (17.1 mg) as a light-yellow oil.^{1}H NMR (700 MHz, $CDCl_3$) δ 7.79 (ddd, *J* = 7.5, 1.2, 0.5 Hz, 1H), 7.48 – 7.45 (m, 1H), 7.43 (d, J = 7.5 Hz, 1H), 7.32 – 7.30 (m, 1H), 7.25 – 7.22 (m, 2H), 7.18 – 7.16 (m, 1H), 6.91 – 6.89 (m, 1H), 5.51 (d, *J* = 8.5 Hz, 1H), 4.11 – 4.06 (m, 1H), 3.18 (s, 3H), 2.90 (d, *J* = 8.9 Hz, 1H). ^{13}C NMR (176 MHz, $CDCl_3$) δ 174.5, 145.4, 138.3, 135.3, 131.7, 129.7, 127.0, 126.2, 126.0, 125.2 (q, *J* = 280.1 Hz), 124.0, 123.2, 122.5, 111.8, 109.3, 70.3, 63.5 (q, *J* = 31.6 Hz), 56.1, 26.7.The er was determined by UPC^2 using a chiral Chiralpack IA column gradient from 100% CO_2 up to 40%; *i*-PrOH, 2.5 mL/min; τ_{major} = 4.35 min, τ_{minor} = 3.63 min, (8.5:91.5 er) $[\alpha]_D^{24}$ = -62.5 (c = 0.7, $CHCl_3$). HRMS calculated for $[C_{19}H_{14}F_3N_3O_3S+K^+]$: 444.0600; found: 444.0591.

3. Enantioselective synthesis of (1*R*,3*S*,3a*S*,8b*S*)-1'-methyl-3a-nitro-1-(trifluoromethyl)-1,2,3a,8b-tetrahydrospiro[benzofuro[2,3-c]pyrrole-3,3'-indolin]-2'-one (3a) on 1 mmol scale

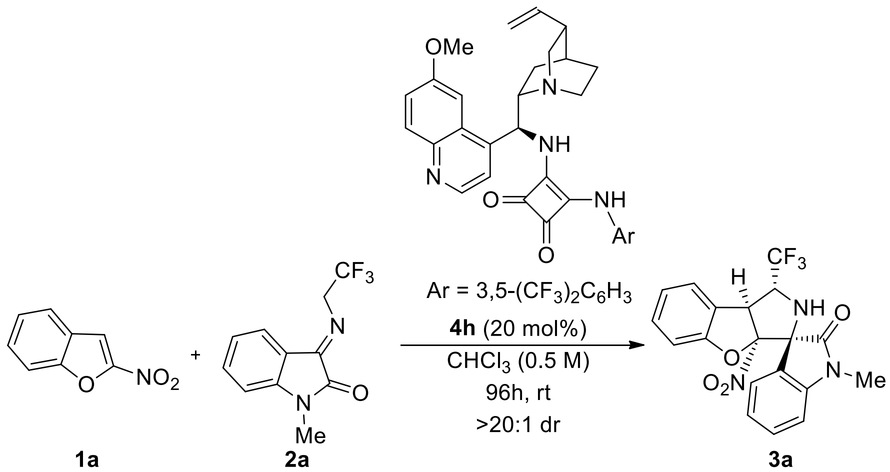

In an ordinary 12 mL glass vial, equipped with a Teflon-coated magnetic stirring bar and a screw cap, 2-nitrobenzofuran **1a** (1.0 equiv., 1.0 mmol, 163 mg), catalyst **4h** (0.2 equiv., 0.2 mmol, 126 mg) and corresponding imine **2a** (1.5 equiv., 0.15 mmol, 363 mg) were dissolved in $CHCl_3$ (6 mL). The reaction mixture was stirred for 48h at ambient temperature and was directly subjected to flash chromatography on silica gel (eluent: from hexanes/dichloromethane 1:1 to 100% dichloromethane) to obtain product **3a** as a single diastereoisomer (>20:1, 95:5 er) in 77% yield (312,1 mg).

4. Transformations of 3a

4.1 Synthesis of (1*R*,3*S*)-1'-methyl-1-(trifluoromethyl)-1,2-dihydrospiro[benzofuro[2,3-c]pyrrole-3,3'-indolin]-2'-one (5)

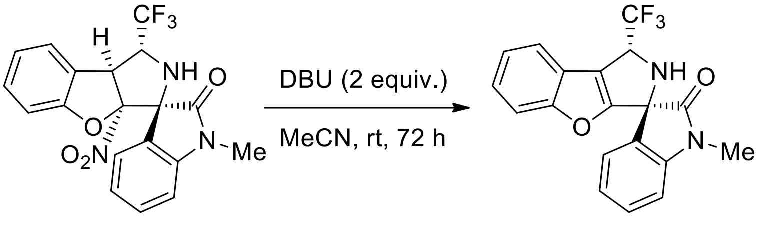

3a **5**

To a stirred solution of **3a** (1.0 equiv, 0.128 mmol, 51 mg) in MeCN (1.5 mL) 1,8-diazabicyclo[5.4.0]undec-7-ene (DBU) (2.0 equiv, 0.256 mmol, 39 mg) was added. The reaction mixture was stirred for 72 h at room temperature and subsequently purified by flash chromatography on silica gel (eluent hexanes/ethyl acetate 4:1) to obtain product **5** as single diastereoisomer (>20:1) in 75 % yield (34.4 mg). (1*R*,3*S*)-1'-Methyl-1-(trifluoromethyl)-1,2-dihydrospiro[benzofuro[2,3-c]pyrrole-3,3'-indolin]-2'-one (**5**). ^{1}H NMR (700 MHz, $CDCl_3$) δ 7.60 – 7.56 (m, 1H), 7.41 – 7.36 (m, 2H), 7.33 – 7.28 (m, 2H), 7.14 (dd, *J* = 7.3, 0.6 Hz, 1H), 7.06 (td, *J* = 7.6, 0.8 Hz, 1H), 6.92 (d, *J* = 7.9 Hz, 1H), 5.27 (p, *J* = 6.1 Hz, 1H), 3.28 (s, 3H), 3.24 (d, *J* = 6.4 Hz, 1H). ^{13}C NMR (176 MHz, $CDCl_3$) δ 173.9, 161.4, 160.2, 144.2, 130.9, 128.3, 125.0, 124.5 (q, *J*=279.4 Hz), 124.0, 123.9, 123.6, 123.4, 120.1, 118.8, 112.7, 109.0, 66.7, 58.6 (q, *J*=34.7 Hz), 26.9. $[\alpha]_D^{20}$ = -5.7 (c = 0.7, $CHCl_3$). HRMS calculated for $[C_{19}H_{13}F_3N_2O_2+Na^+]$: 381.0821; found: 381.0827.

4.2 Synthesis of (1*R*,3*S*,3a*S*,8b*S*)-1'-methyl-1-(trifluoromethyl)-1,2,3a,8b-tetrahydrospiro[benzofuro[2,3-c]pyrrole-3,3'-indolin]-2'-one (6)

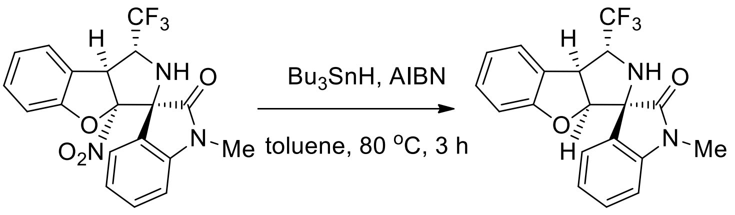

3a **6**

To a stirred solution of **3a** (1.0 equiv., 0.05 mmol, 20.3 mg) in dry toluene (0.5 mL) tributyltin hydride (4.0 equiv., 0.2 mmol, 58 mg) and AIBN (2.0 equiv., 0.1 mmol, 16.4 mg) were added in room temperature. The reaction mixture was stirred for 3 h at 80 ^{o}C, cooled to room

temperature and CCl_4 (0.15 mL) was added dropwise. After stirring for 5 minutes saturated KF aq. solution (10 mL) was added and resulting mixture was extracted with AcOEt (3 x 10 mL). Combined organic layers were dried over Na_2SO_4, filtered and concentrated under reduced pressure to obtain crude product, which was purified by flash chromatography on silica gel (eluent: hexanes/ dichlorometane 1:1 to dichloromethane 100%) to obtain product **6** as single diastereoisomer (>20:1) in 68 % yield (12,3 mg). (1*R*,3*S*,3a*S*,8b*S*)-1'-methyl-1-(trifluoromethyl)-1,2,3a,8b-tetrahydrospiro[benzofuro[2,3-c]pyrrole-3,3'-indolin]-2'-one (**6**). ^{1}H NMR (700 MHz, $CDCl_3$) δ 7.42 (ddd, *J* = 7.4, 1.3, 0.6 Hz, 1H), 7.35 (td, *J* = 7.7, 1.3 Hz, 1H), 7.30 (dd, *J* = 7.5, 1.3 Hz, 1H), 7.19 (dddd, *J* = 8.1, 7.5, 1.4, 0.7 Hz, 1H), 7.13 (td, *J* = 7.5, 1.0 Hz, 1H), 6.95 (td, *J* = 7.5, 1.0 Hz, 1H), 6.83 (dt, *J* = 7.7, 0.7 Hz, 1H), 6.73 (ddt, *J* = 8.1, 1.0, 0.5 Hz, 1H), 5.33 (d, *J* = 10.3 Hz, 1H), 4.74 (qd, *J* = 6.6, 4.9 Hz, 1H), 4.31 (dd, *J* = 10.3, 6.1 Hz, 1H), 3.13 (s, 3H), 2.23 (d, *J* = 4.9 Hz, 1H). 13C NMR (176 MHz, $CDCl_3$) δ 177.0, 160.2, 144.3, 130.1, 129.5, 128.9, 126.7, 125.68 (q, *J* = 278.2 Hz), 124.7, 123.9, 123.4, 121.6, 109.7, 108.5, 91.8, 72.5, 65.37 (q, J = 30.1 Hz), 47.0, 26.3. $[\alpha]_D^{19}$= +29,4 (c = 0.7, $CHCl_3$). HRMS calculated for [$C_{19}H_{15}F_3N_2O_2$+Na^+]: 383.0978; found: 383.0983.

5. Crystal and X-ray data for (1*R*,3*S*,3a*S*,8b*S*)-1'-methyl-3a-nitro-1-(trifluoromethyl)-1,2,3a,8b-tetrahydrospiro[benzofuro[2,3-c]pyrrole-3,3'-indolin]-2'-one (3a)

The single-crystal X-ray diffraction study at a low temperature of 100 K revealed that compound **3a** ($C_{19}H_{14}F_3N_3O_4$) crystallizes in the non-centrosymmetric orthorhombic space group $P2_12_12_1$ (Z = 4) and the crystal structure consists of one crystallographically independent formula unit in the unit cell (Figure 1).

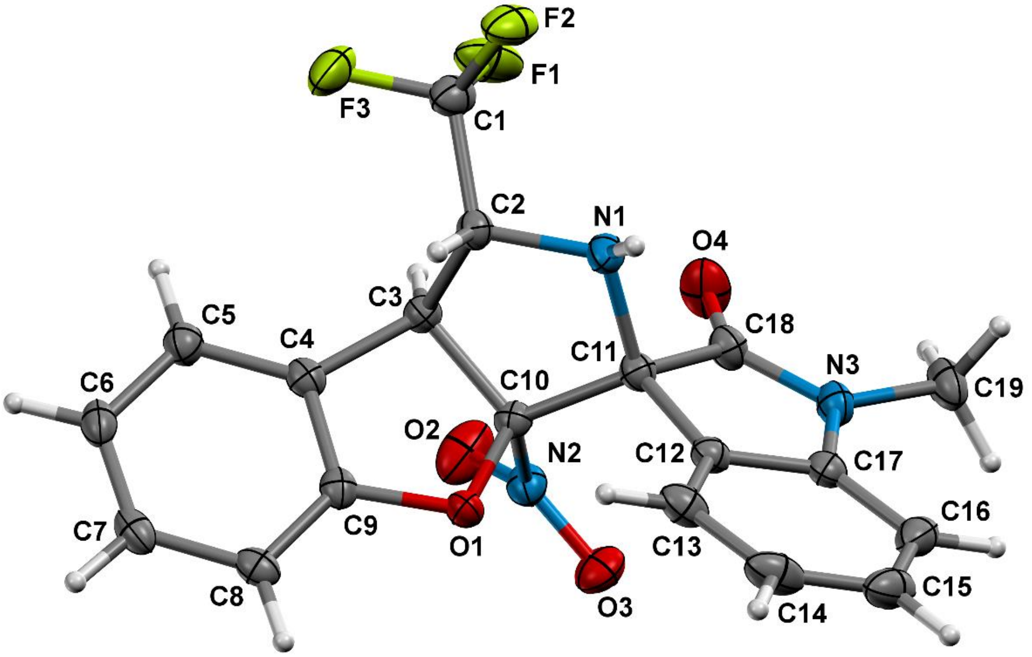

Figure 1. The molecular structure of the compound **3a** at 100 K, with atom numbering. The displacement ellipsoids are drawn at the 50% probability level. Hydrogen atoms are drawn with an arbitrary radius.

Single crystal X-ray diffraction analysis was performed at 100 K by the ω-scan technique on a RIGAKU XtaLAB Synergy, Dualflex, Pilatus 300K diffractometer[4] with PhotonJet micro-focus X-ray Source Cu-Kα (λ = 1.54184 Å). Data collection, cell refinement, data reduction and absorption correction were performed using CrysAlis PRO software [4]. The crystal structure was solved by using direct methods with the SHELXT 2018/2 program [5]. Atomic scattering factors were taken from the International Tables for X-ray Crystallography. Positional parameters of non-H-atoms were refined by a full-matrix least-squares method on F^2 with anisotropic thermal parameters by using the SHELXL 2018/3 program [6]. All hydrogen atoms were found from the difference Fourier maps. The N-H hydrogen atom was refined freely with an isotropic displacement parameter, and all other hydrogen atoms were refined with a riding model.

3a: Formula $C_{19}H_{14}F_3N_3O_4$, monoclinic, space group $P2_12_12_1$, Z = 4, unit cell constants a = 9.3913(1), b = 12.9280(1), c = 14.3277(1) Å, V = 1739.54(3) Å^3. A total of 48421 reflections angles in the range of 4.61 to 70.07° were collected of which 3294 were unique (R_{int} = 2.27%) and 3290 of these were greater than 2σ(I). The final anisotropic full-matrix least-squares refinement on F^2 with 268 parameters converged at R_1 = 2.40% and wR_2 = 6.13% for all data. The largest peak in the final difference electron density synthesis was 0.197 e Å^{-3} and the largest hole was -0.129 e Å^{-3}. The goodness-of-fit was 1.112. The absolute configuration was determined from anomalous scattering, by calculating the x Flack parameter [7] of 0.05(2) using 1384 quotients.

CCDC 2091820 contains the supplementary crystallographic data for this paper. These data can be obtained free of charge from The Cambridge Crystallographic Data Centre via www.ccdc.cam.ac.uk/structures

4. Rigaku OD. CrysAlis PRO. Rigaku Oxford Diffraction Ltd, Yarnton, Oxfordshire, England, **2019**.
5. Sheldrick, G. M. SHELXT - Integrated space-group and crystal-structure determination. *Acta Cryst. A71*, **2015**, 3-8.
6. Sheldrick, G. M. Crystal structure refinement with SHELXL. *Acta Cryst. C71*, **2015**, , 3-8.
7. Parsons, S.; Flack, H. D.; Wagner, T. Use of intensity quotients and differences in absolute structure refinement *Acta Cryst. B69*, **2013**, 249-259.

6. NMR data

(1*R*,3*S*,3a*S*,8b*S*)-1'-Methyl-3a-nitro-1-(trifluoromethyl)-1,2,3a,8b-tetrahydrospiro[benzofuro[2,3-c]pyrrole-3,3'-indolin]-2'-one (3a)

^{1}H NMR

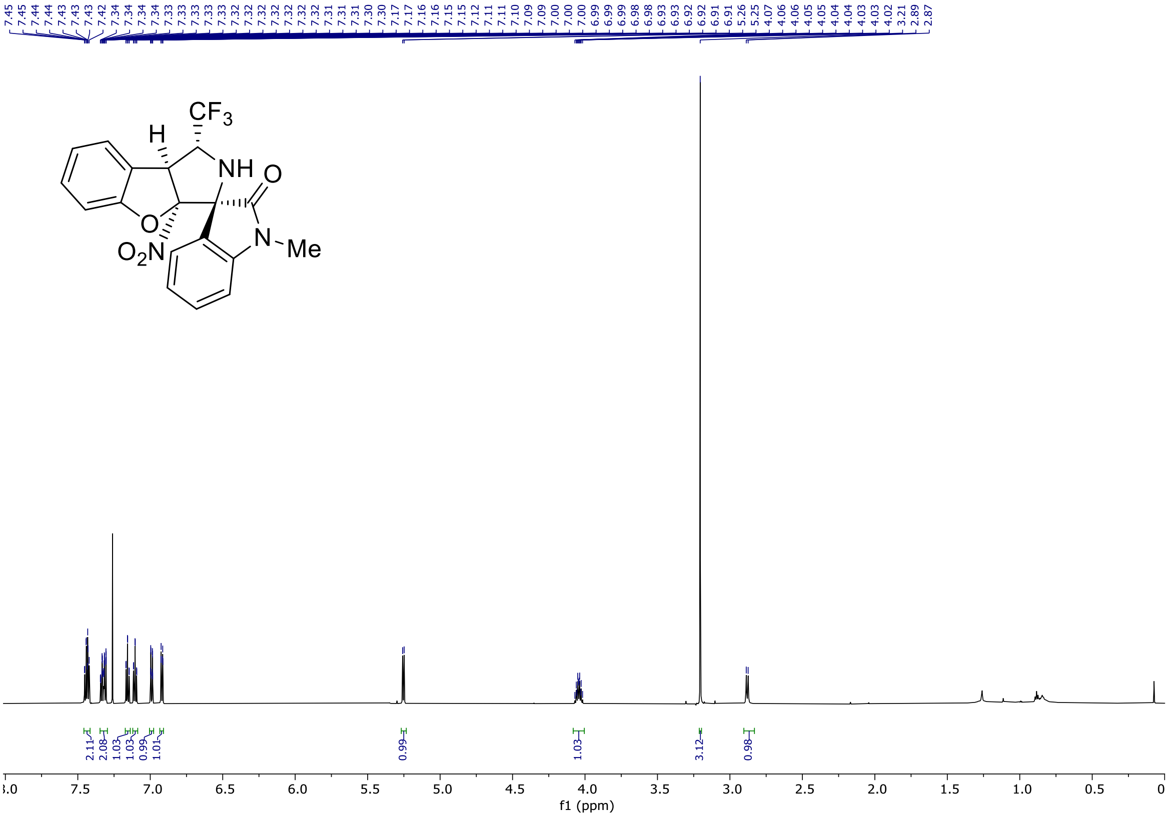

^{13}C NMR

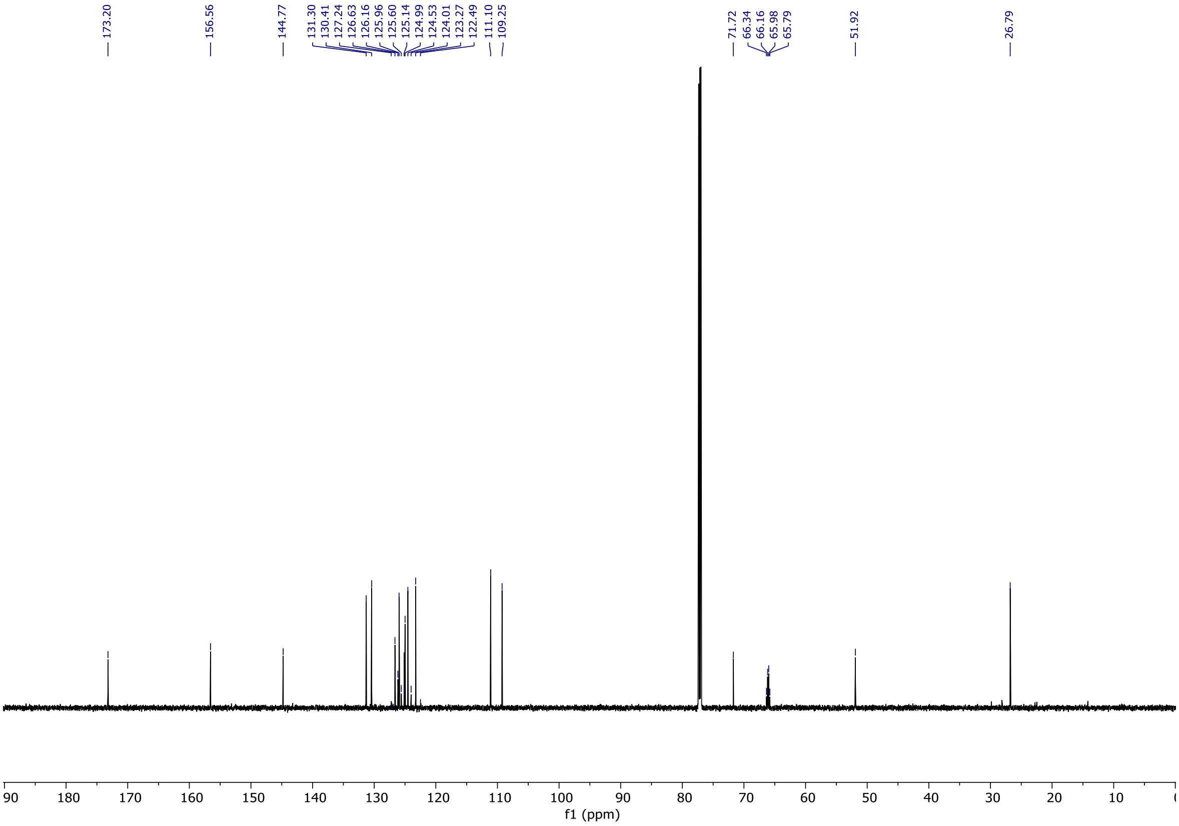

(1*R*,3*S*,3a*S*,8b*S*)-1'-Allyl-3a-nitro-1-(trifluoromethyl)-1,2,3a,8b-tetrahydrospiro [benzofuro[2,3-c]pyrrole-3,3'-indolin]-2'-one (3b)

^{1}H NMR

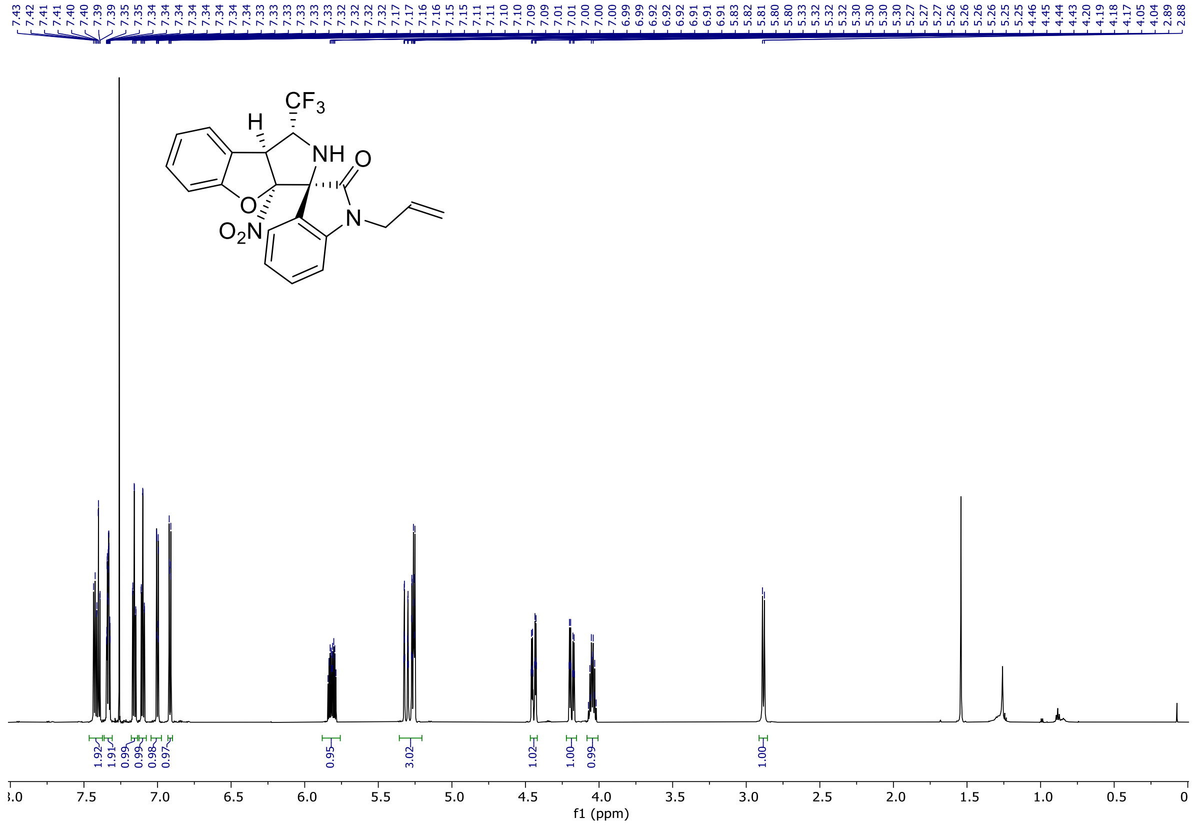

^{13}C NMR

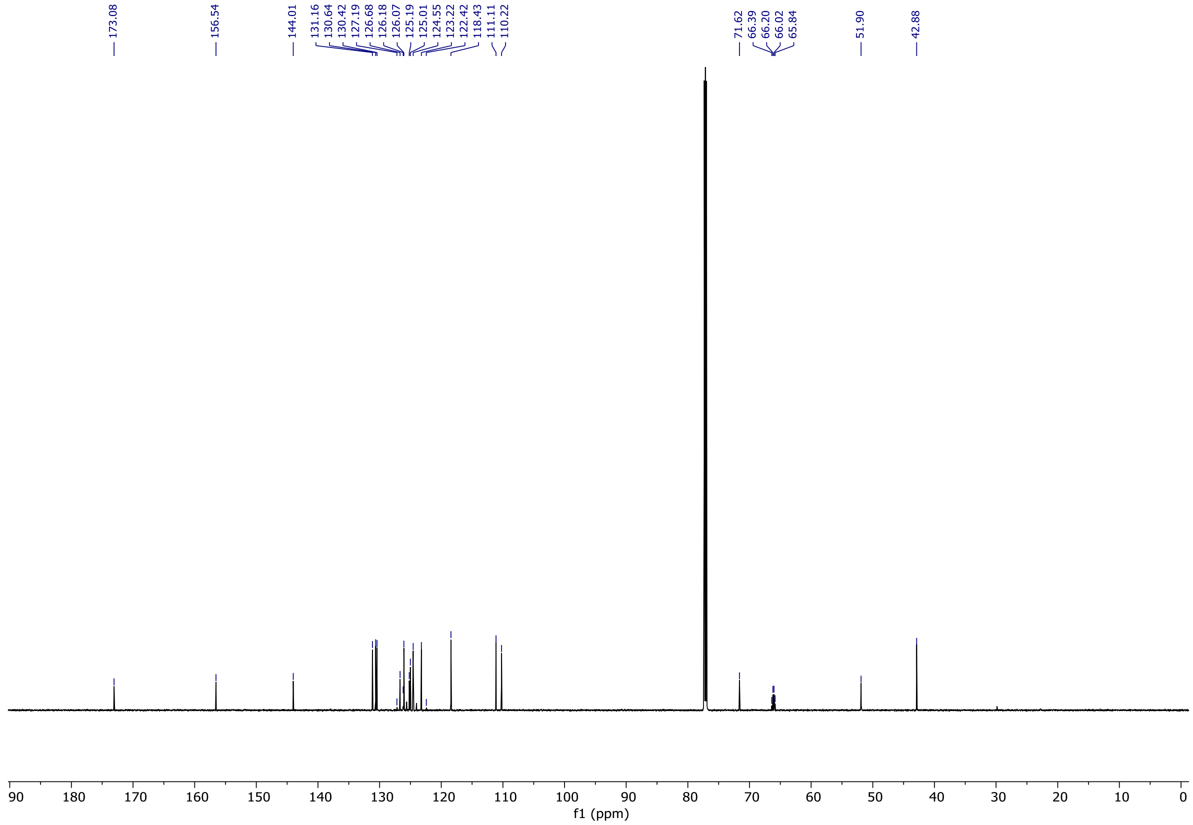

(1*R*,3*S*,3a*S*,8b*S*)-1'-Benzyl-3a-nitro-1-(trifluoromethyl)-1,2,3a,8b-tetrahydrospiro[benzofuro[2,3-c]pyrrole-3,3'-indolin]-2'-one (3c)

^{1}H NMR

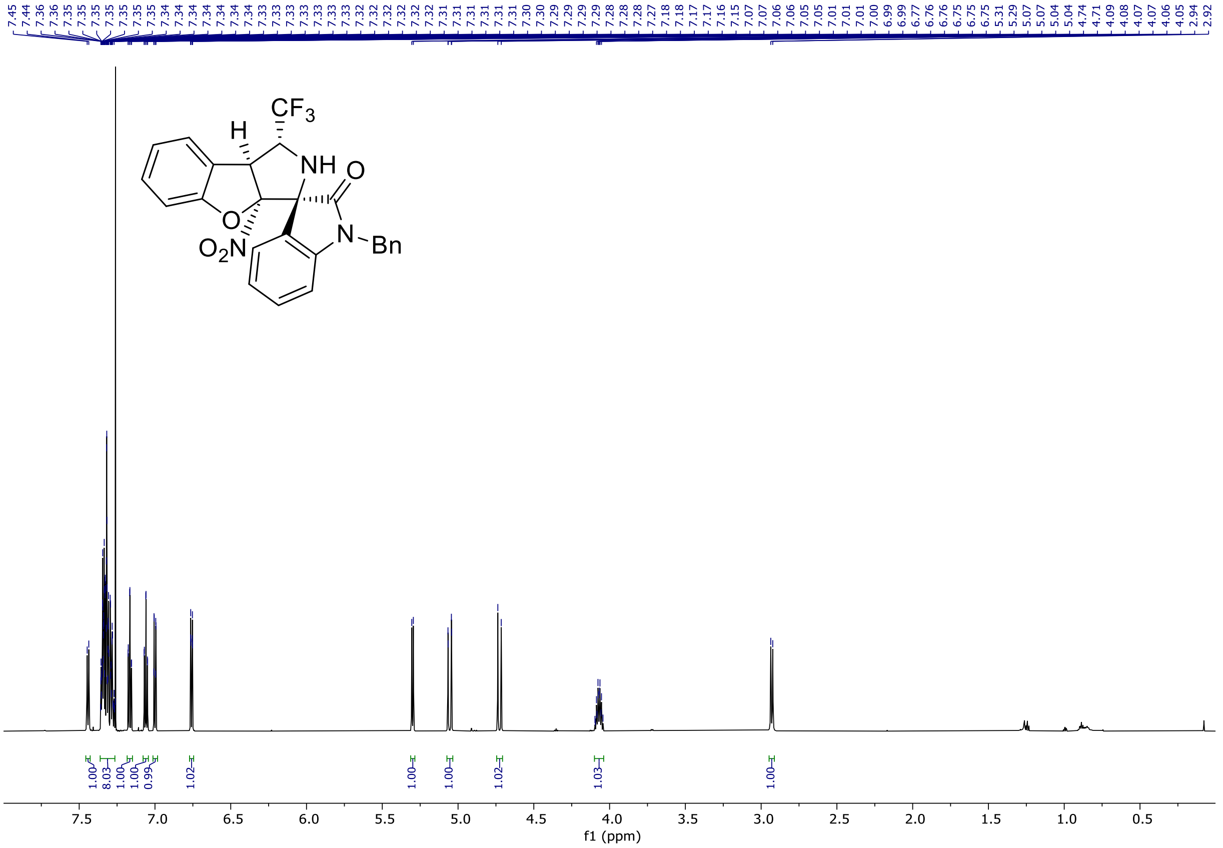

^{13}C NMR

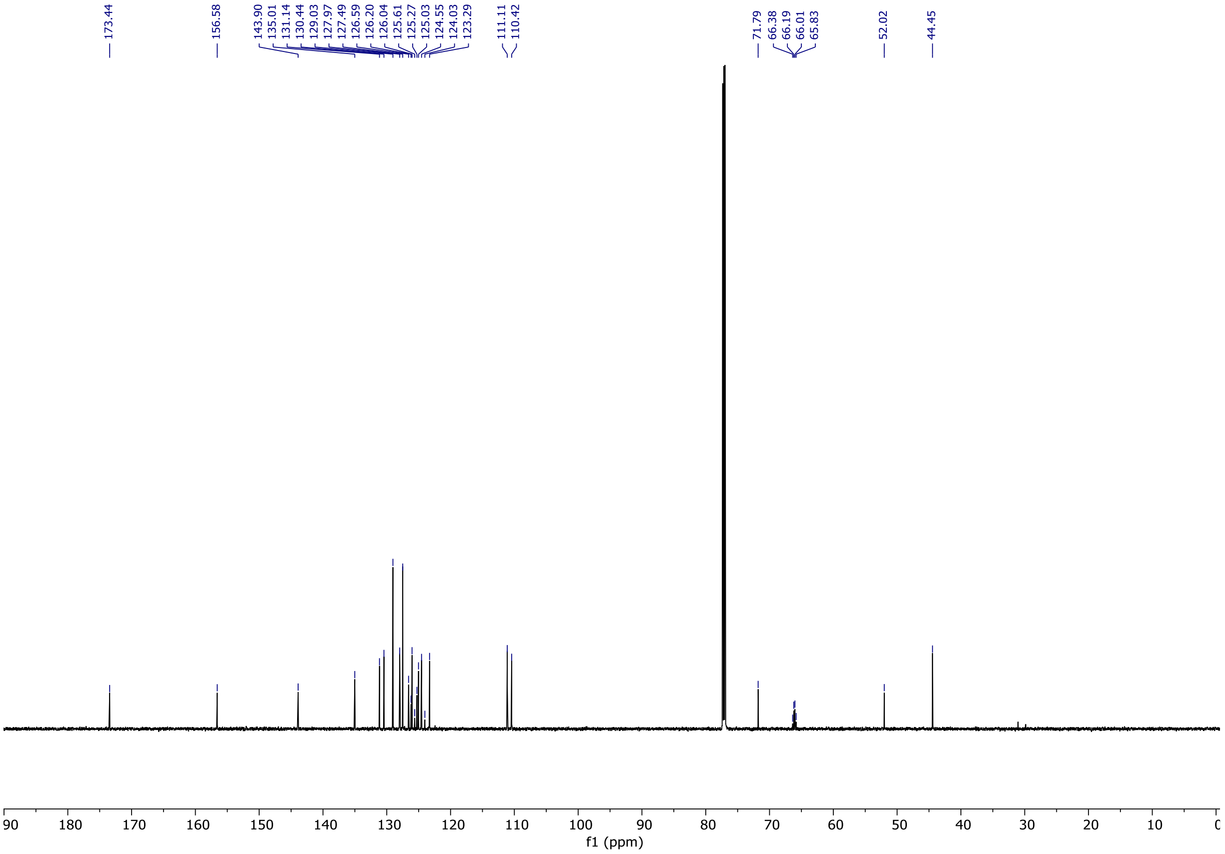

(1*R*,3*S*,3a*S*,8b*S*)-3a-Nitro-1-(trifluoromethyl)-1,2,3a,8b-tetrahydrospiro[benzofuro[2,3-c]pyrrole-3,3'-indolin]-2'-one (3d)

^{1}H NMR

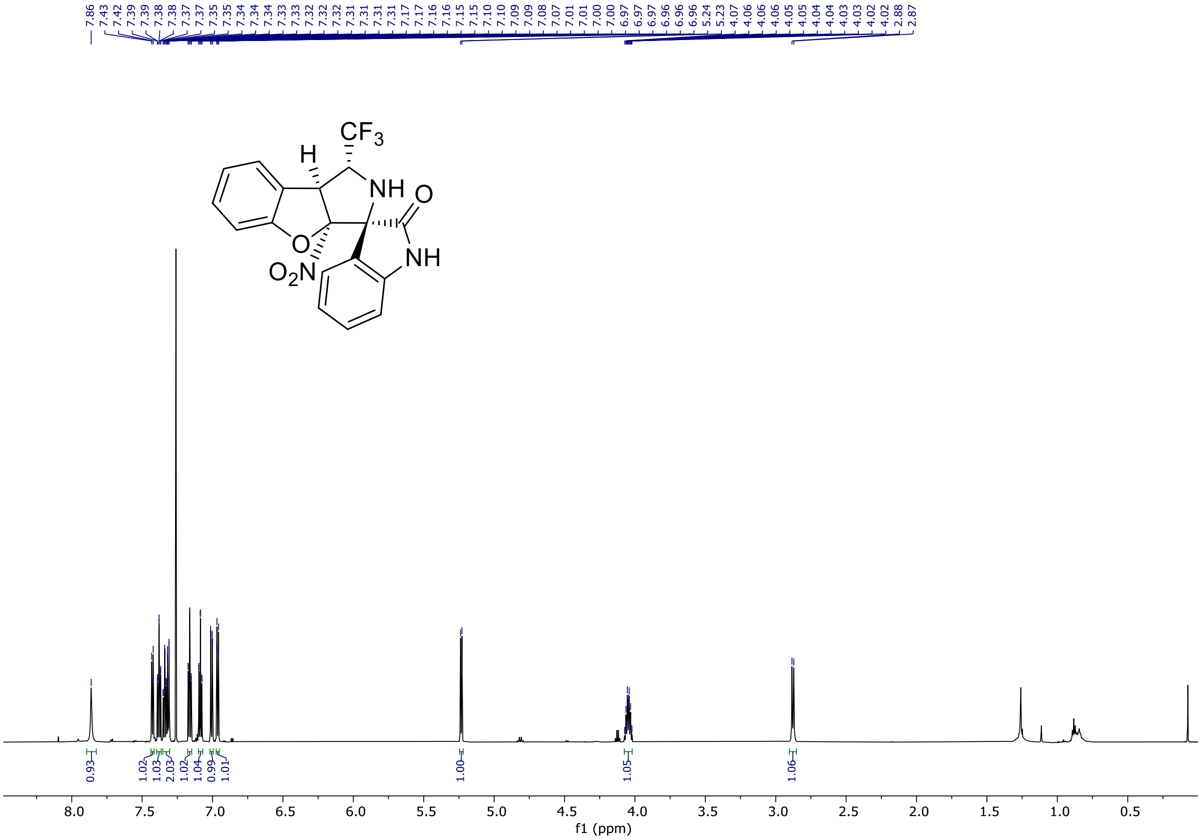

^{13}C NMR

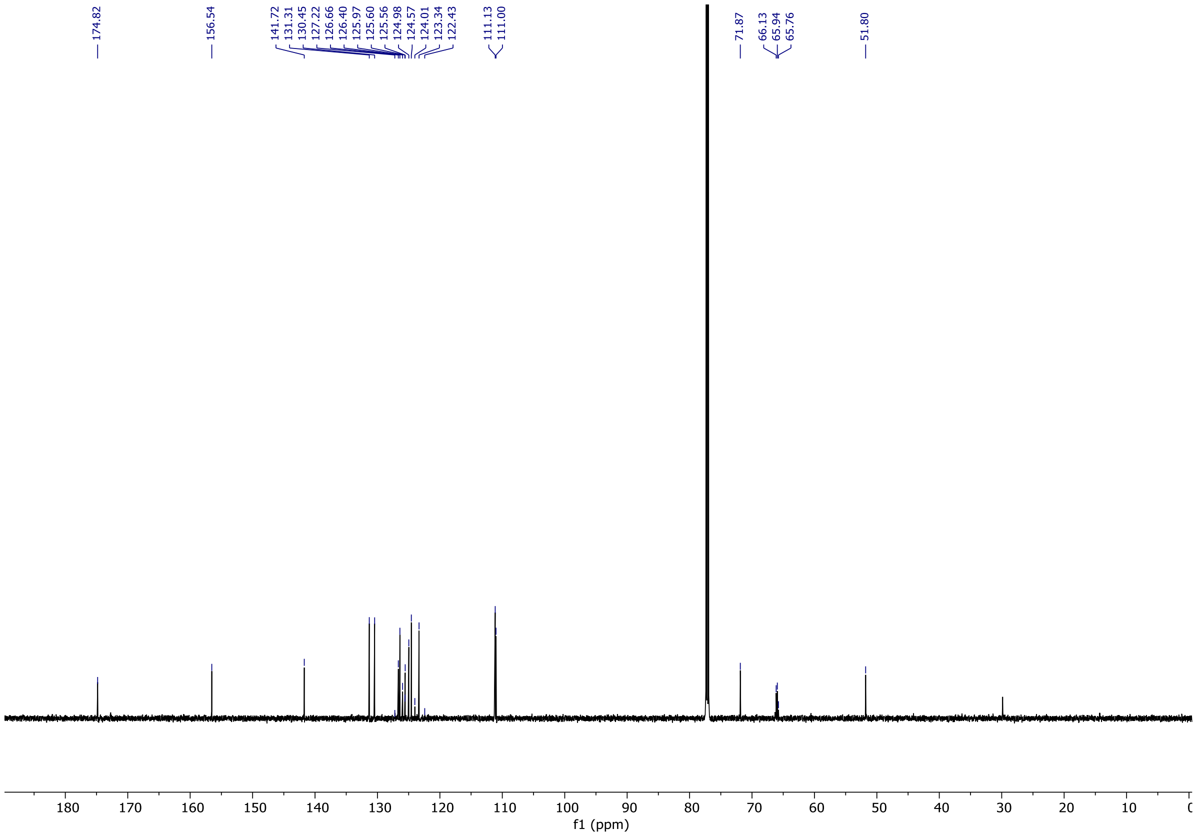

(1*R*,3*S*,3a*S*,8b*S*)-5'-Methoxy-3a-nitro-1-(trifluoromethyl)-1,2,3a,8b-tetrahydrospiro[benzofuro[2,3-c]pyrrole-3,3'-indolin]-2'-one (3e)

^{1}H NMR

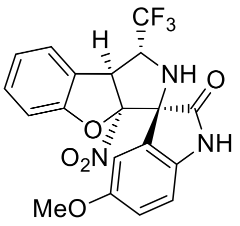

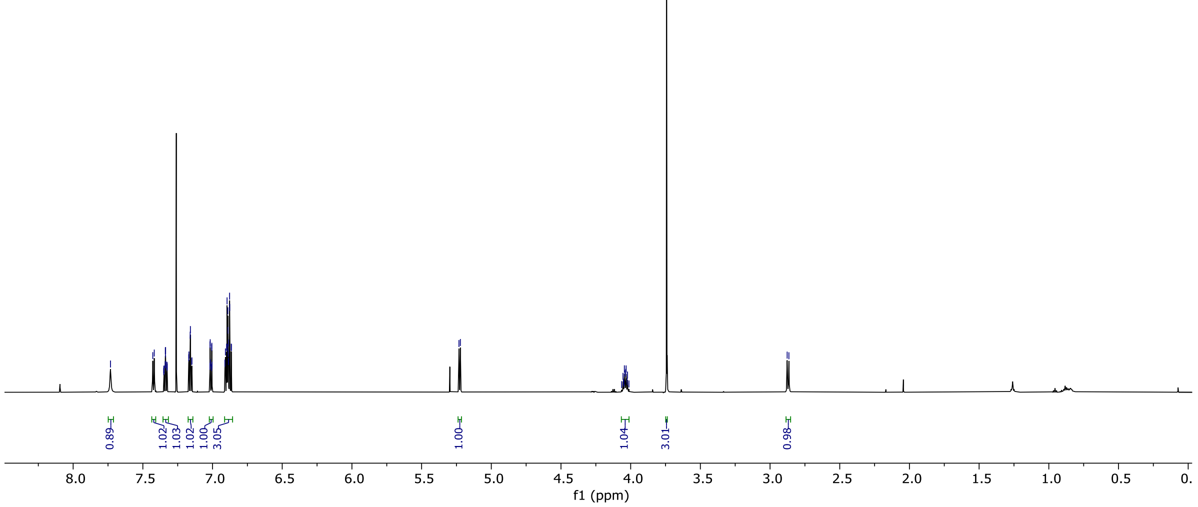

^{13}C NMR

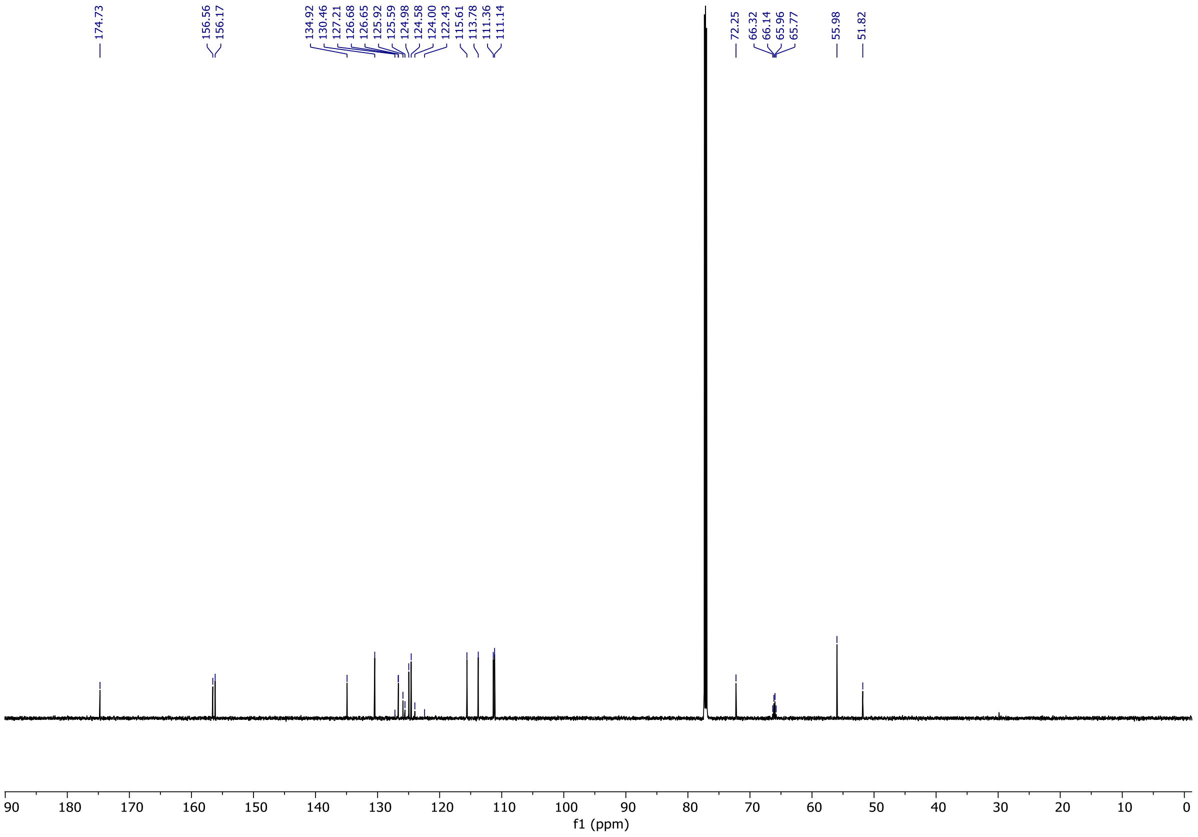

(1*R*,3*S*,3a*S*,8b*S*)-5'-Methyl-3a-nitro-1-(trifluoromethyl)-1,2,3a,8b-tetrahydrospiro[benzofuro[2,3-c]pyrrole-3,3'-indolin]-2'-one (3f)

^{1}H NMR

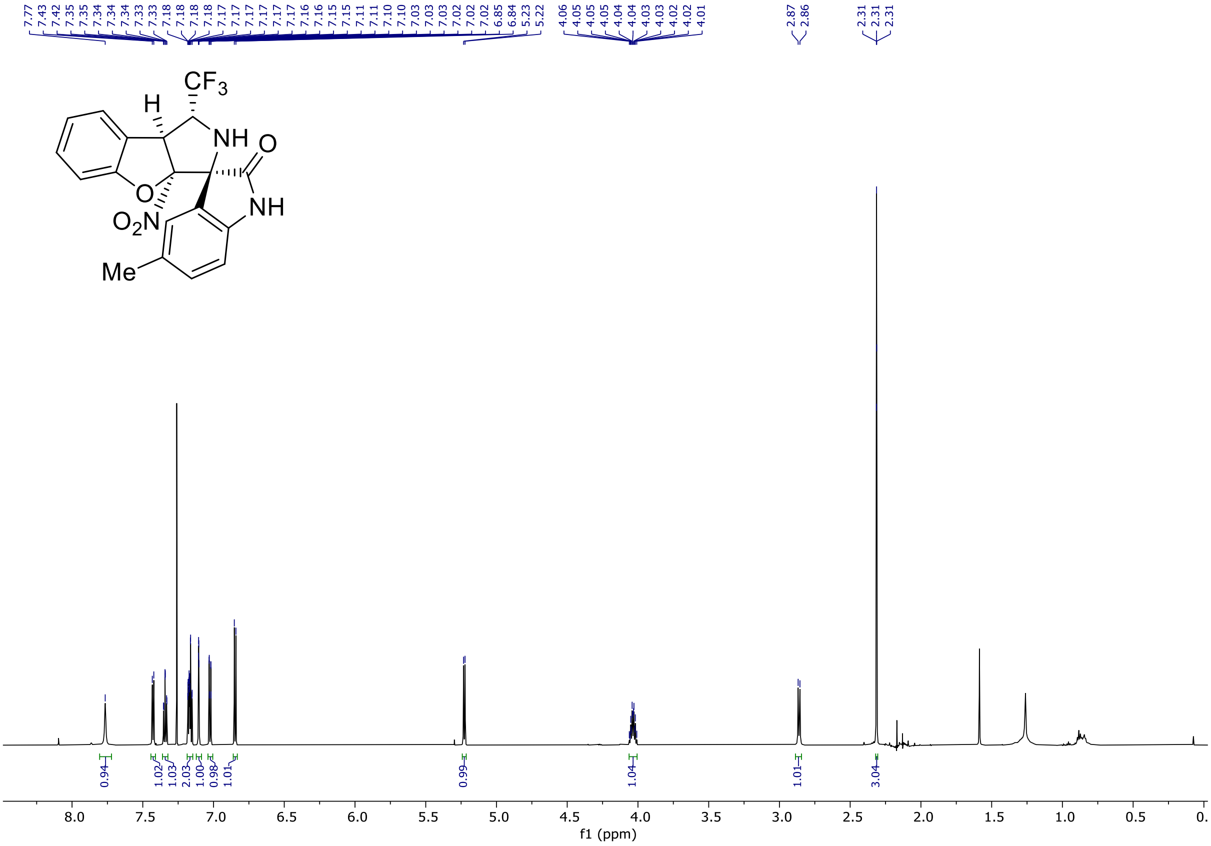

^{13}C NMR

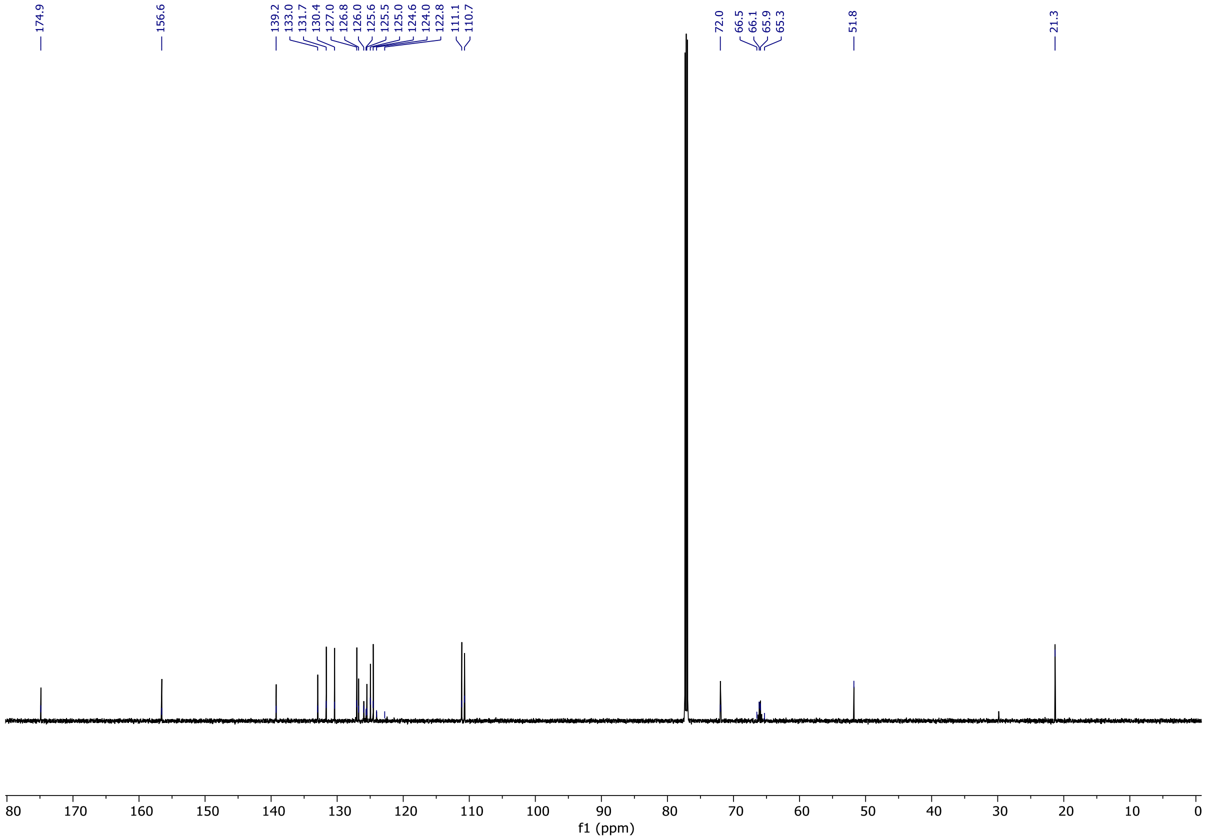

(1*R*,3*S*,3a*S*,8b*S*)-5'-Bromo-3a-nitro-1-(trifluoromethyl)-1,2,3a,8b-tetrahydrospiro[benzofuro[2,3-c]pyrrole-3,3'-indolin]-2'-one (3g)

^{1}H NMR

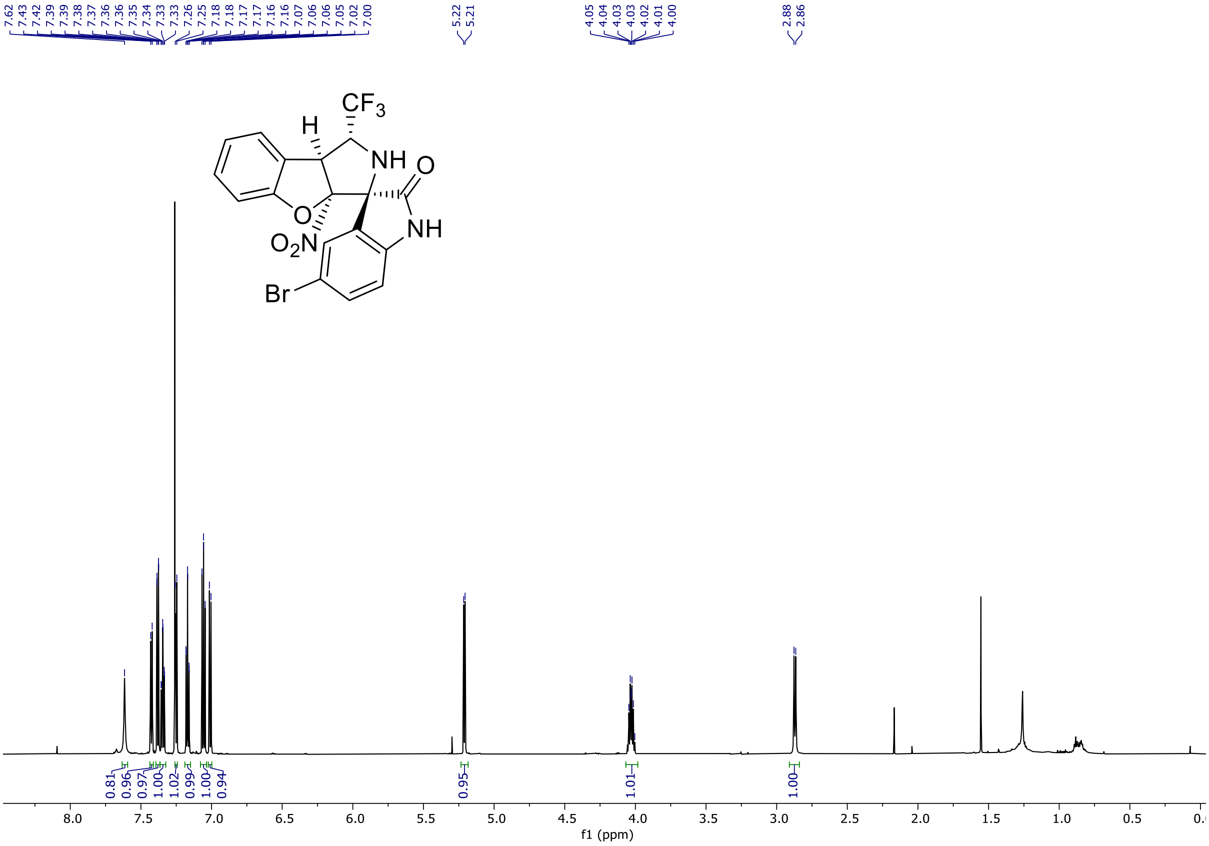

^{13}C NMR

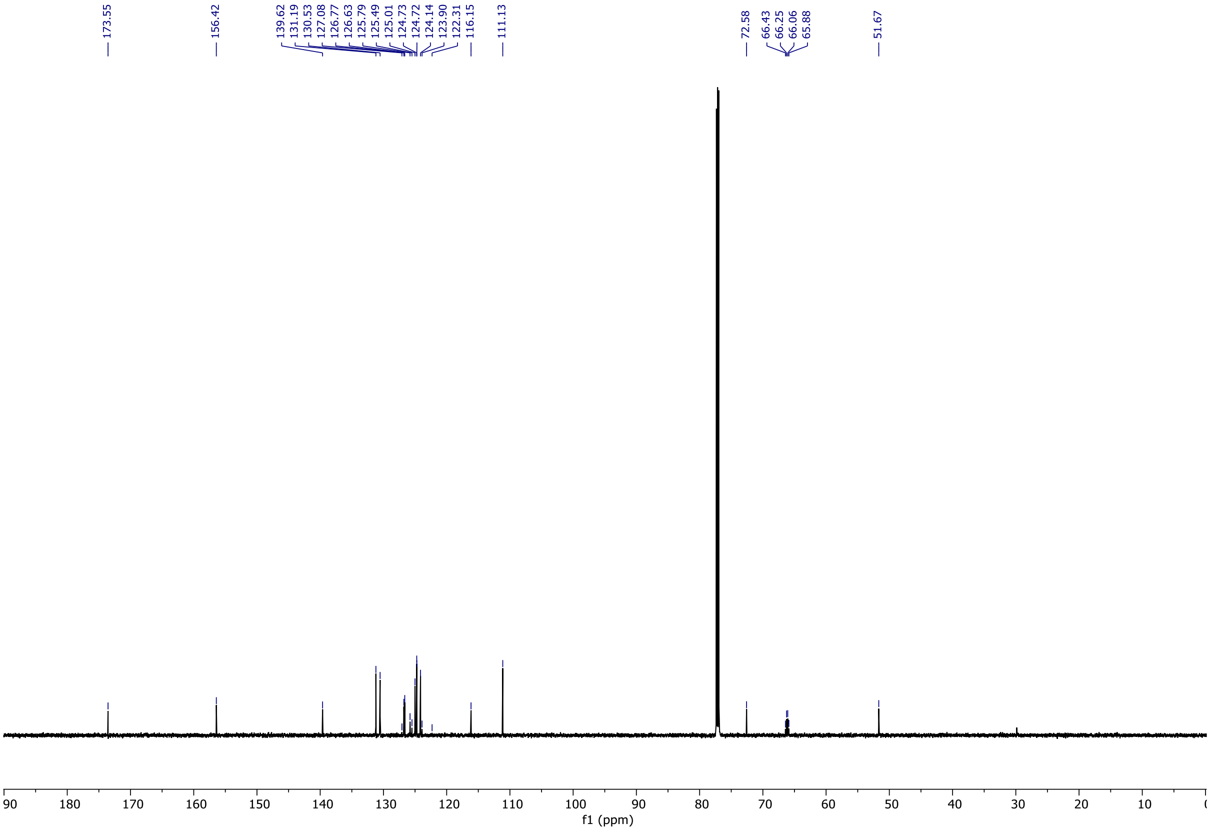

(1*R*,3*S*,3a*S*,8b*S*)-5'-Chloro-3a-nitro-1-(trifluoromethyl)-1,2,3a,8b-tetrahydrospiro[benzofuro[2,3-c]pyrrole-3,3'-indolin]-2'-one (3h)

^{1}H NMR

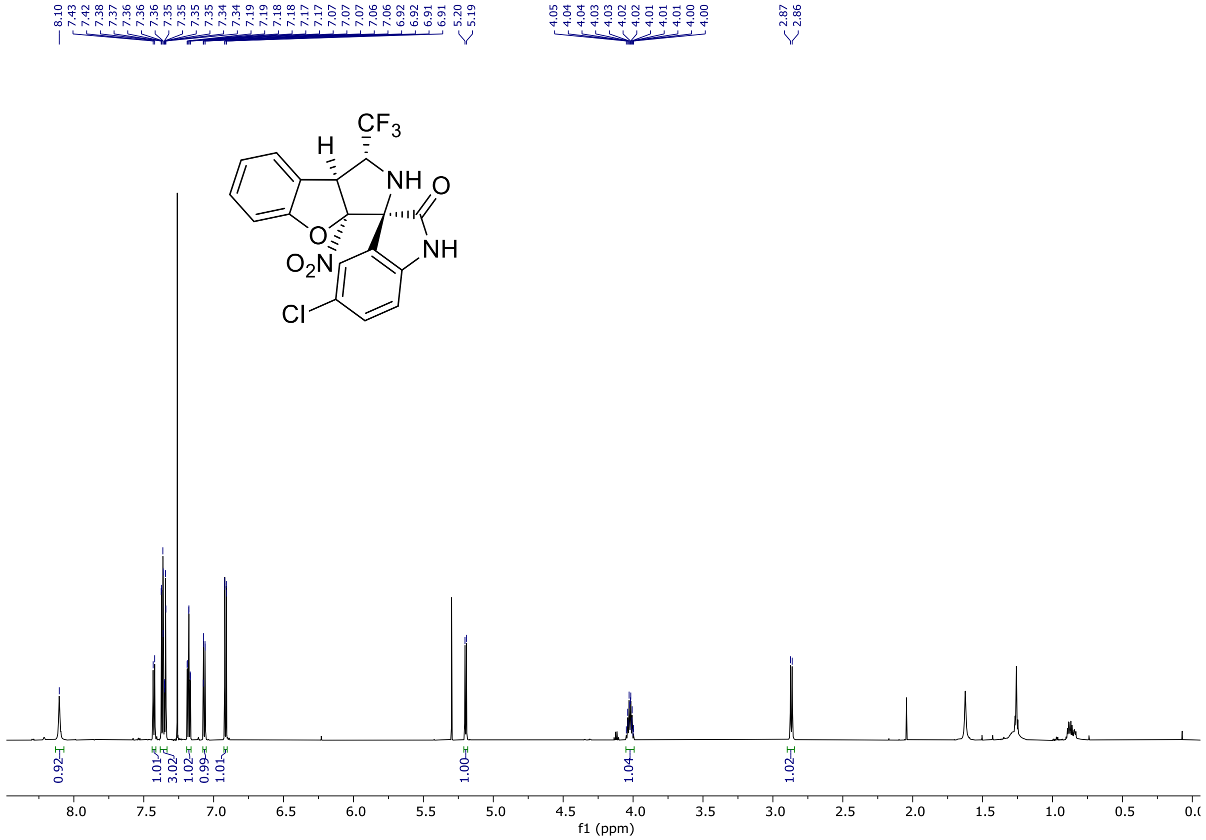

^{13}C NMR

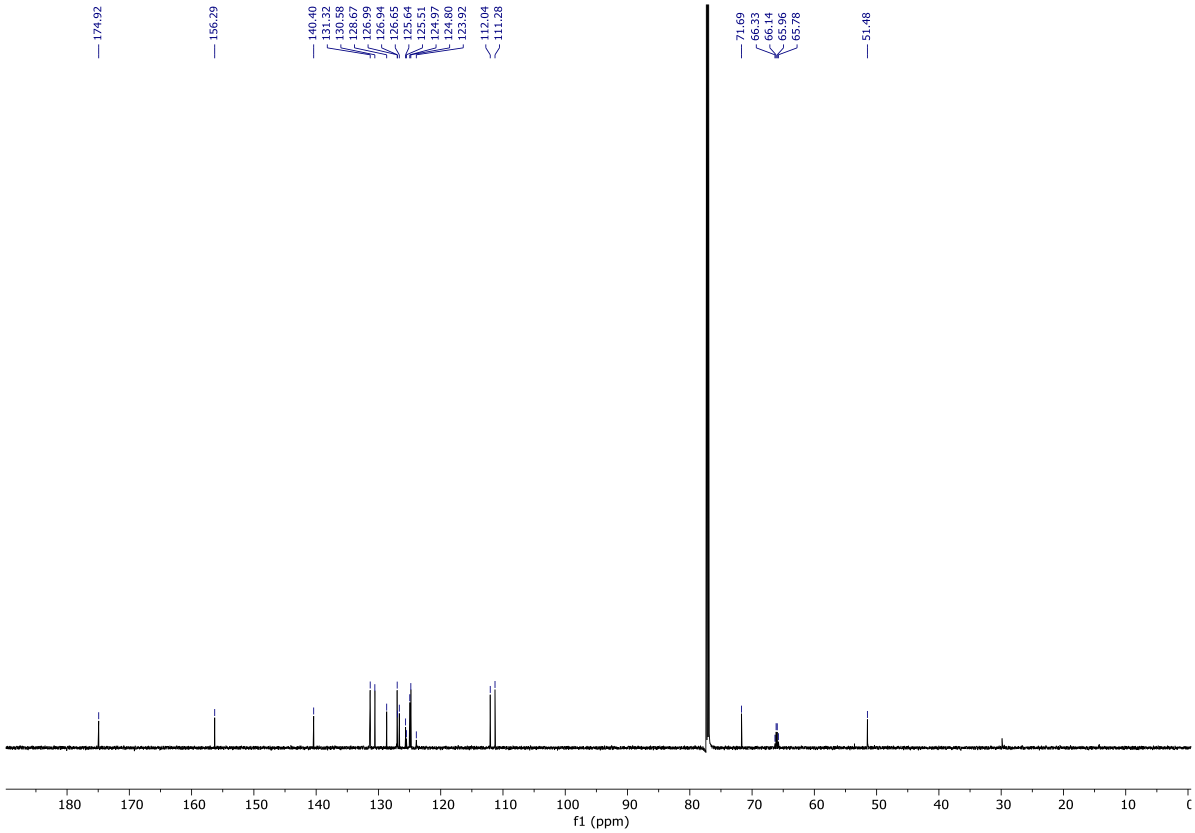

(1*R*,3*S*,3a*S*,8b*S*)-7'-Chloro-3a-nitro-1-(trifluoromethyl)-1,2,3a,8b-tetrahydrospiro[benzofuro[2,3-c]pyrrole-3,3'-indolin]-2'-one (3i)

^{1}H NMR

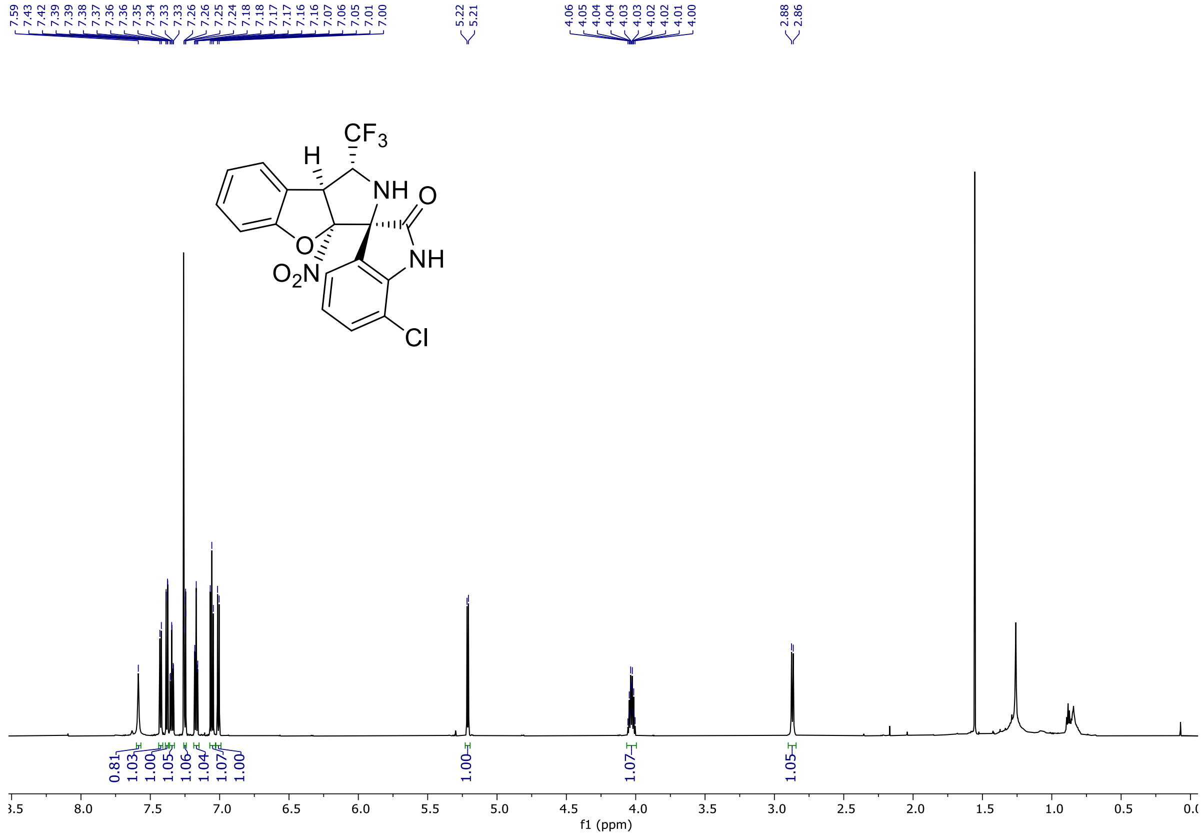

^{13}C NMR

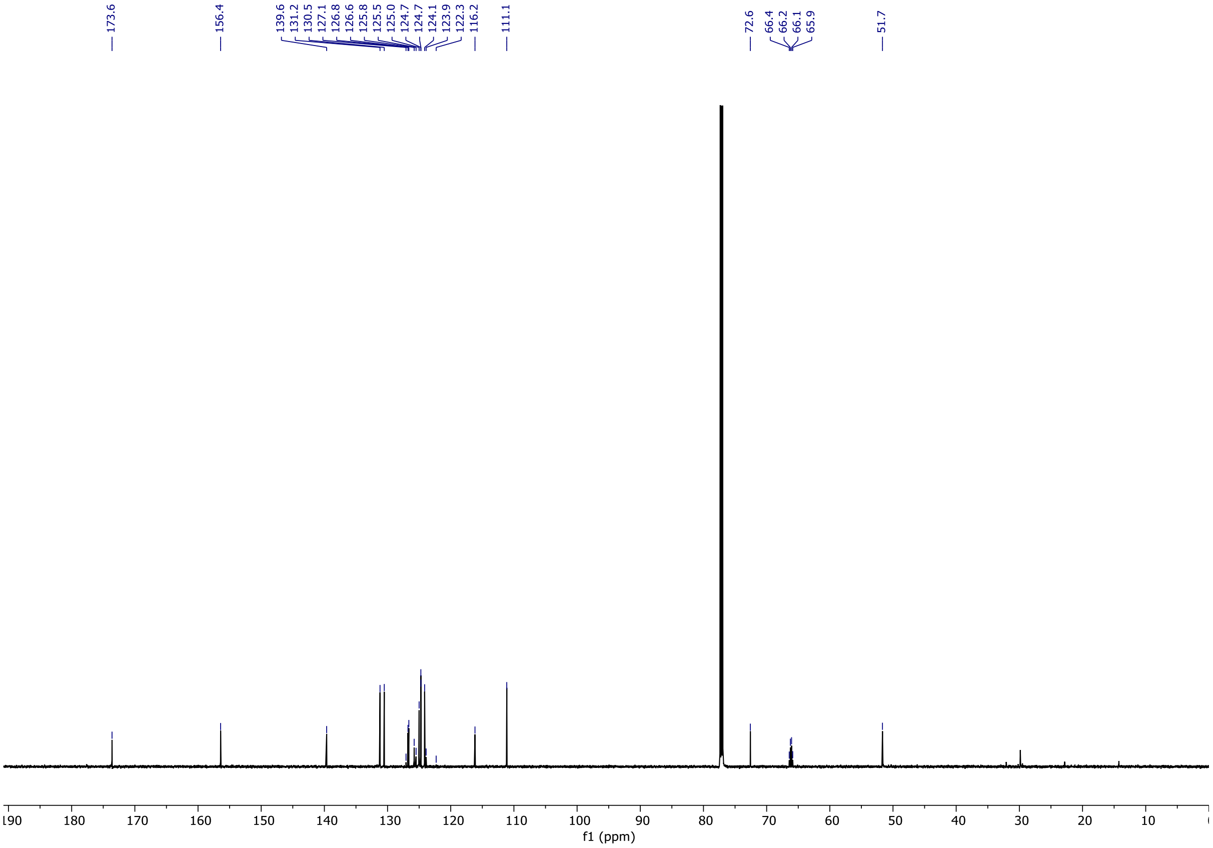

(1*R*,3*S*,3a*S*,8b*S*)-3a,5'-Dinitro-1-(trifluoromethyl)-1,2,3a,8b-tetrahydrospiro[benzofuro[2,3-c]pyrrole-3,3'-indolin]-2'-one (3j)

^{1}H NMR

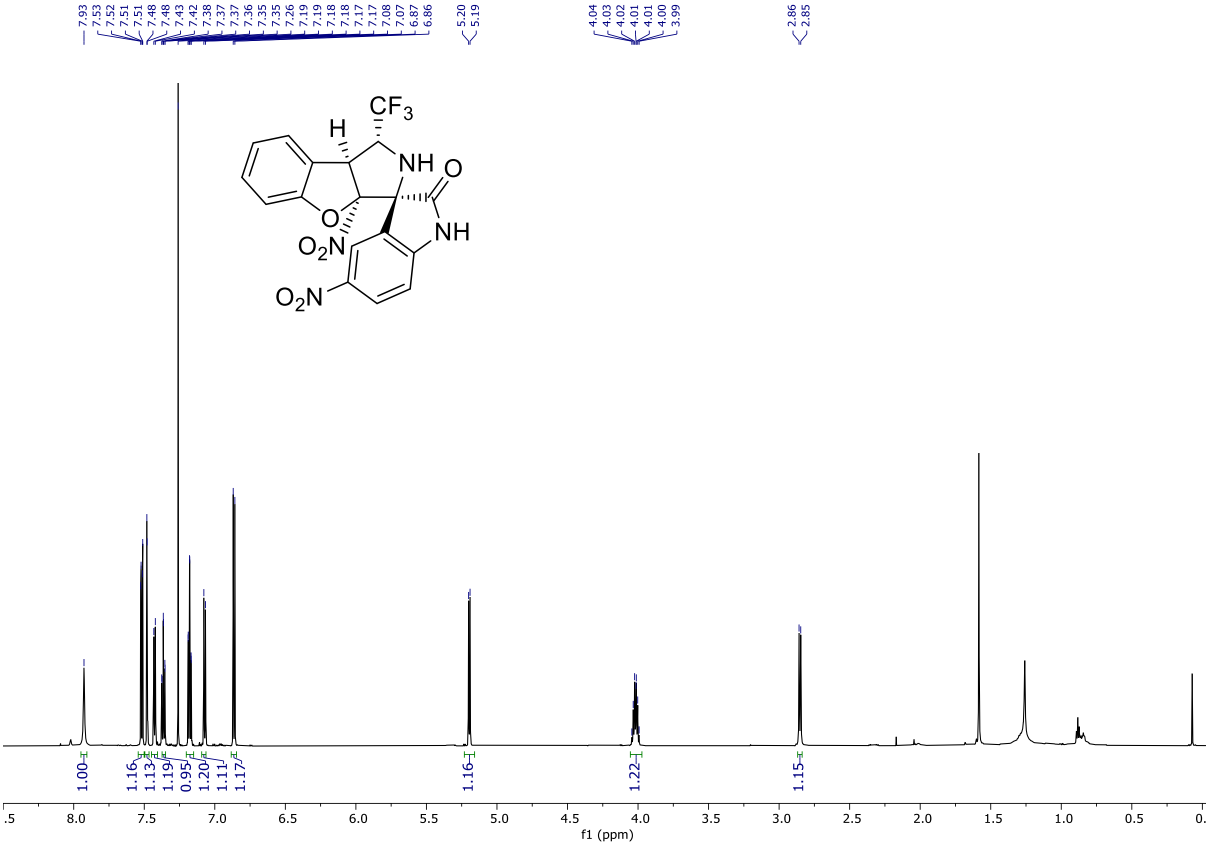

^{13}C NMR

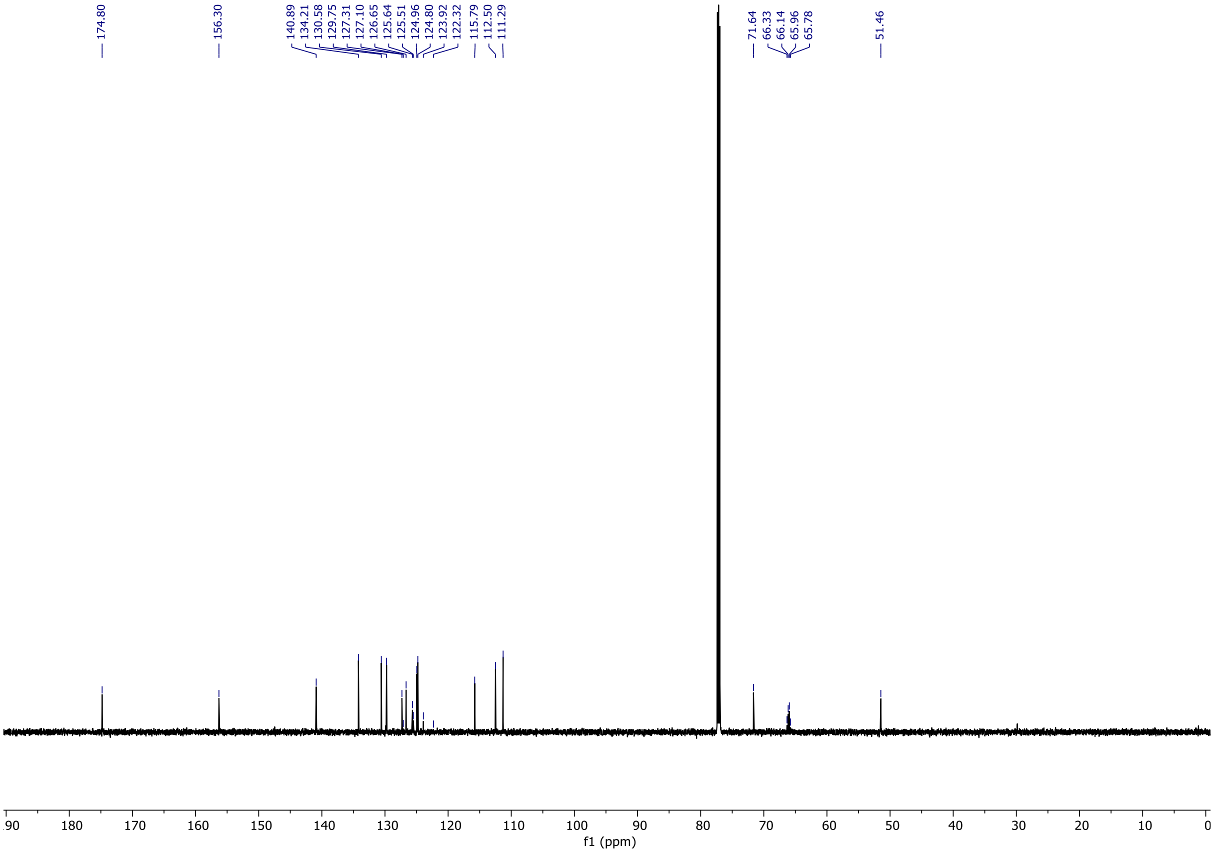

(1*R*,3*S*,3a*S*,8b*S*)-5',7'-Dibromo-3a-nitro-1-(trifluoromethyl)-1,2,3a,8b-tetrahydrospiro[benzofuro[2,3-c]pyrrole-3,3'-indolin]-2'-one (3k)

^{1}H NMR

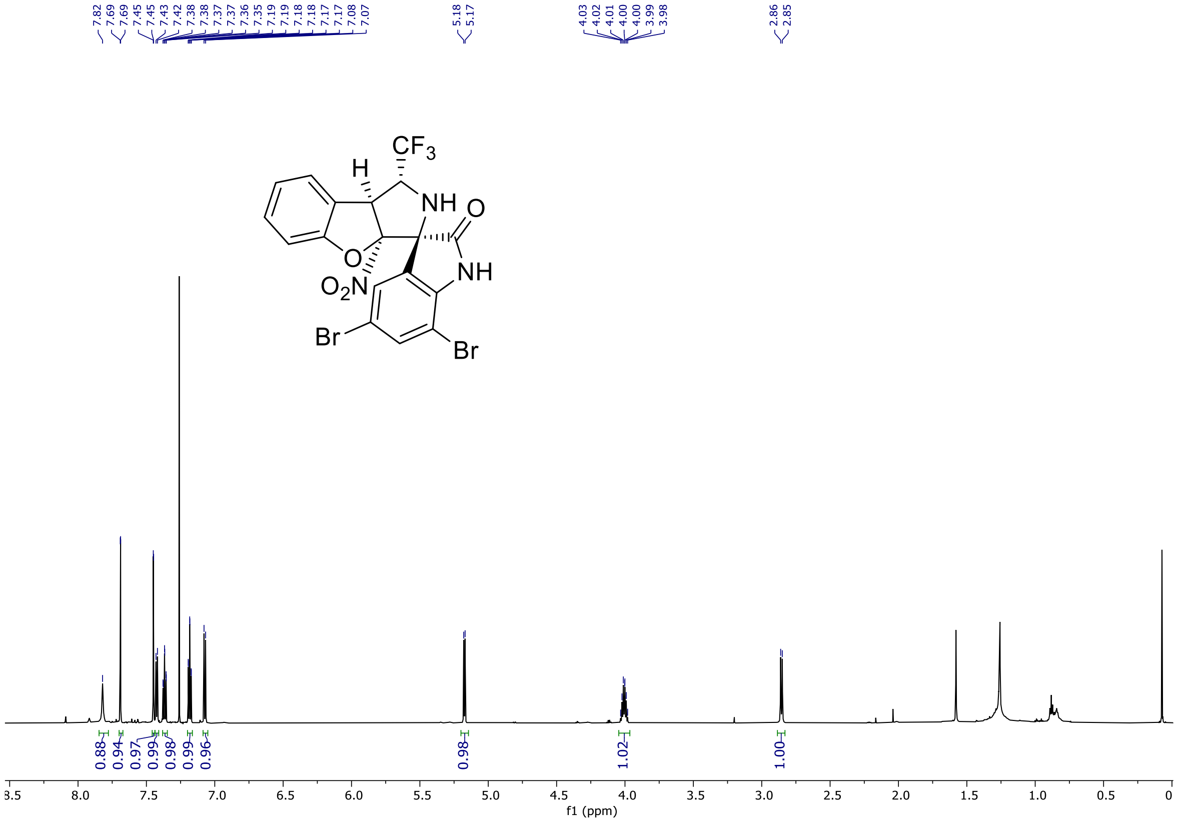

^{13}C NMR

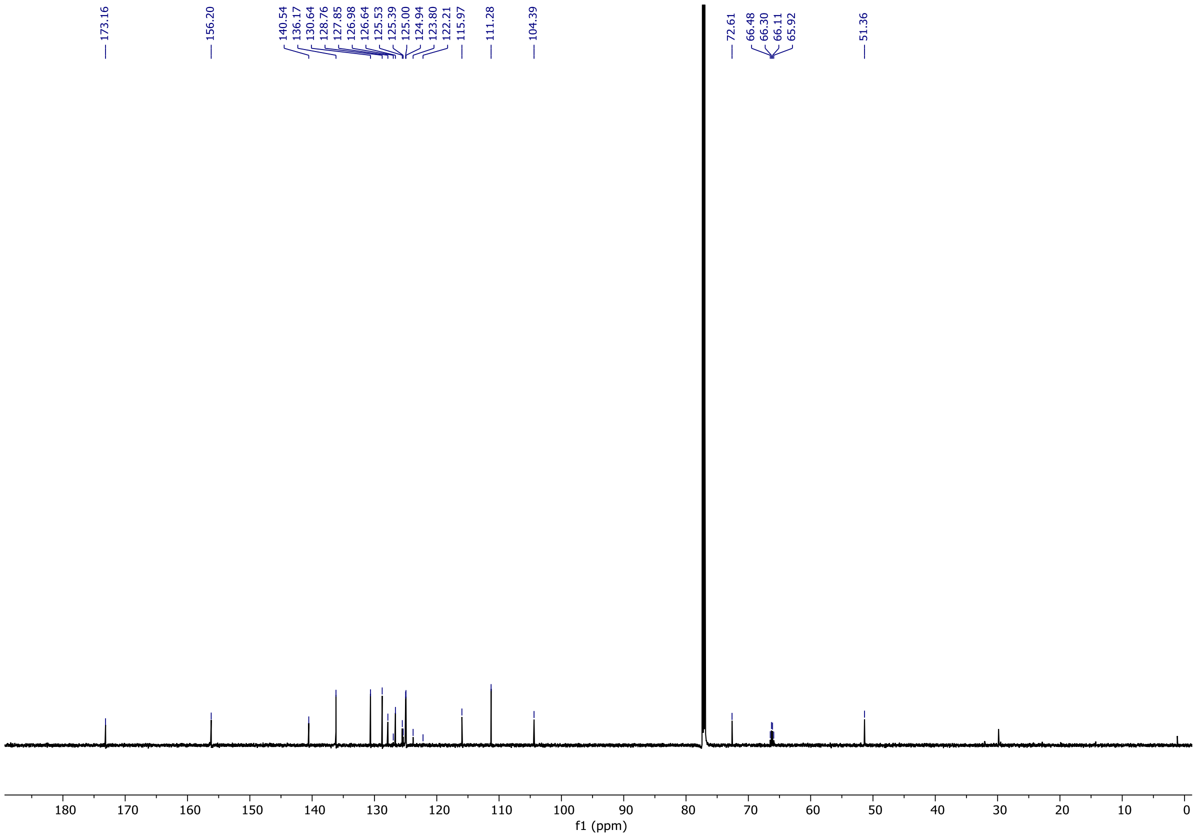

(1*R*,3*S*,3a*S*,8b*S*)-7-Methoxy-1'-methyl-3a-nitro-1-(trifluoromethyl)-1,2,3a,8b-tetrahydrospiro[benzofuro[2,3-c]pyrrole-3,3'-indolin]-2'-one (3l)

^{1}H NMR

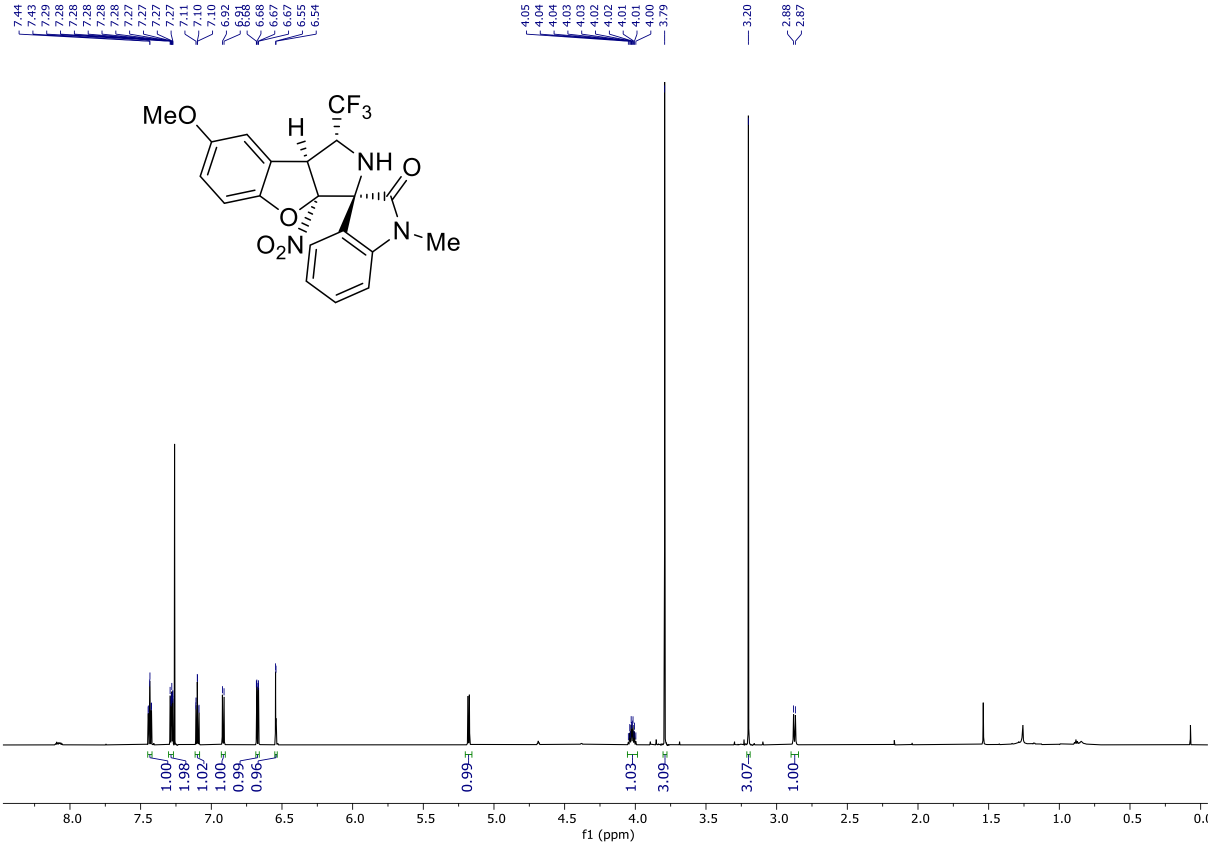

^{13}C NMR

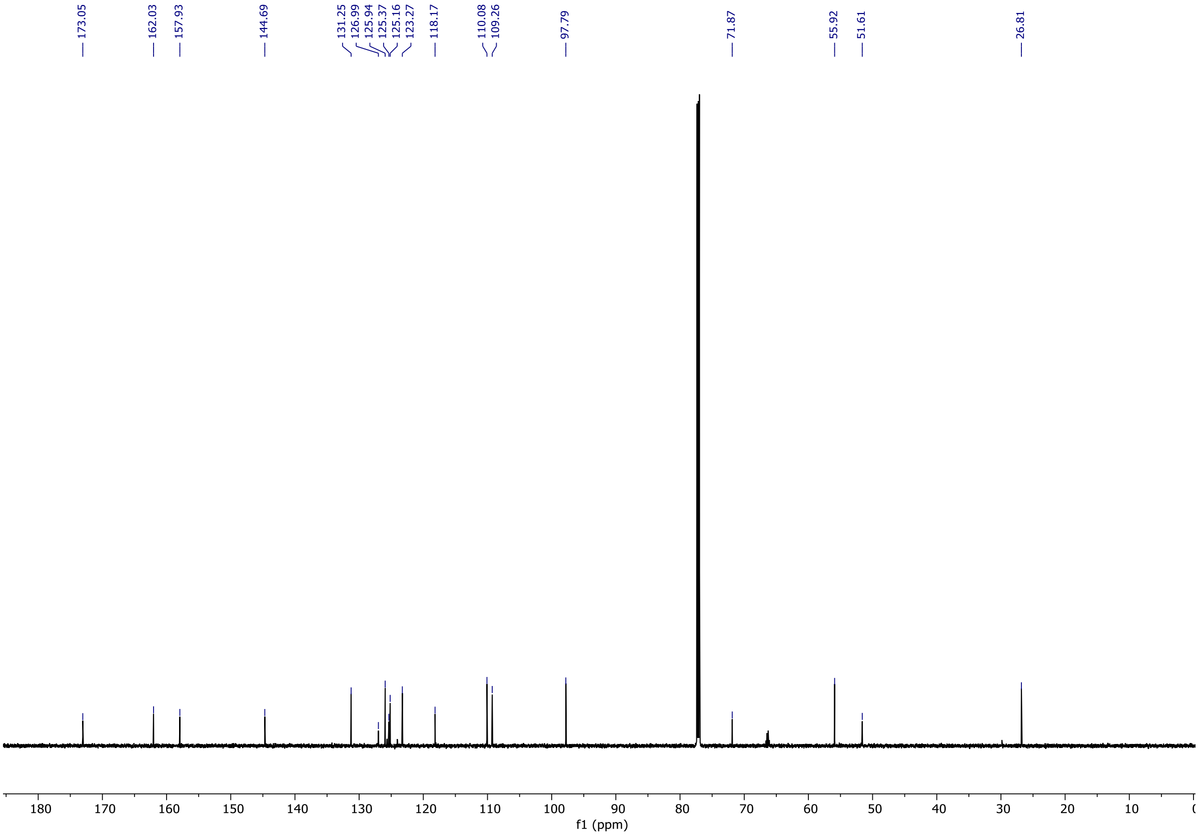

(1*R*,3*S*,3a*S*,8b*S*)-1',7-Dimethyl-3a-nitro-1-(trifluoromethyl)-1,2,3a,8b-tetrahydrospiro[benzofuro[2,3-c]pyrrole-3,3'-indolin]-2'-one (3m)

^{1}H NMR

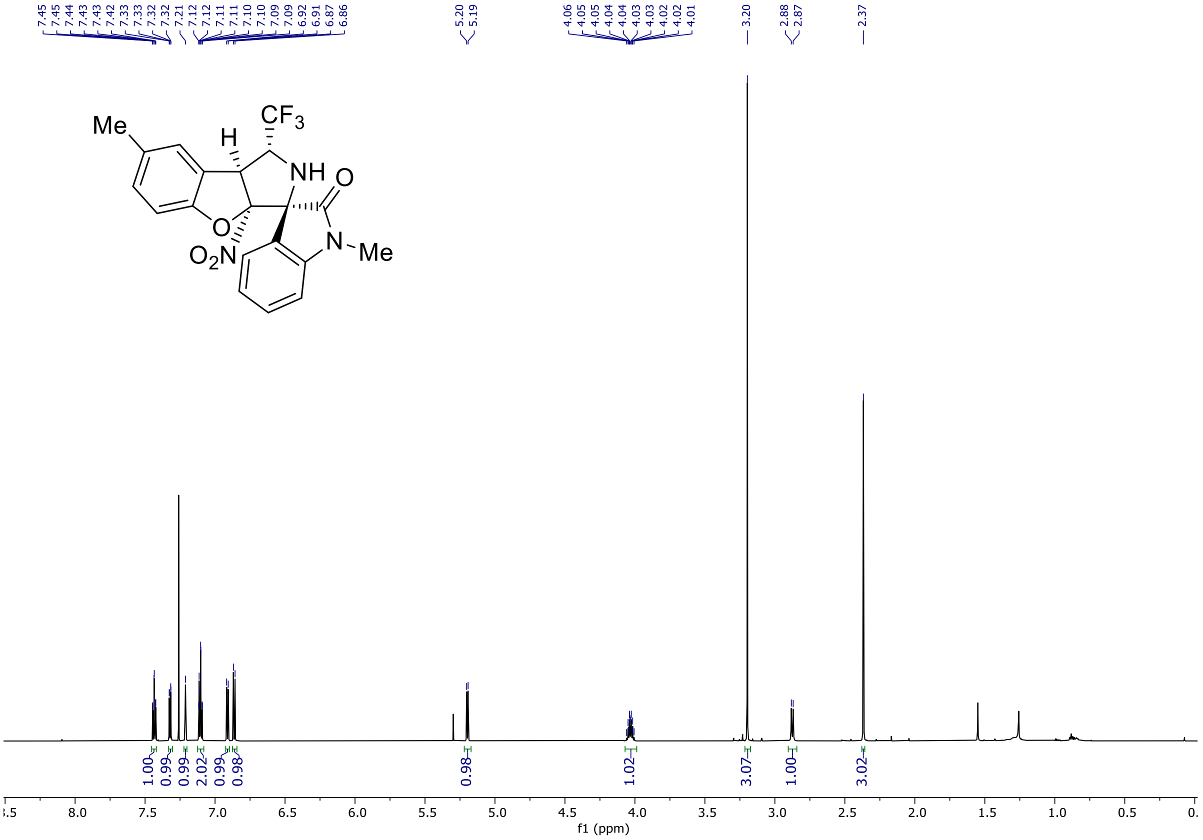

^{13}C NMR

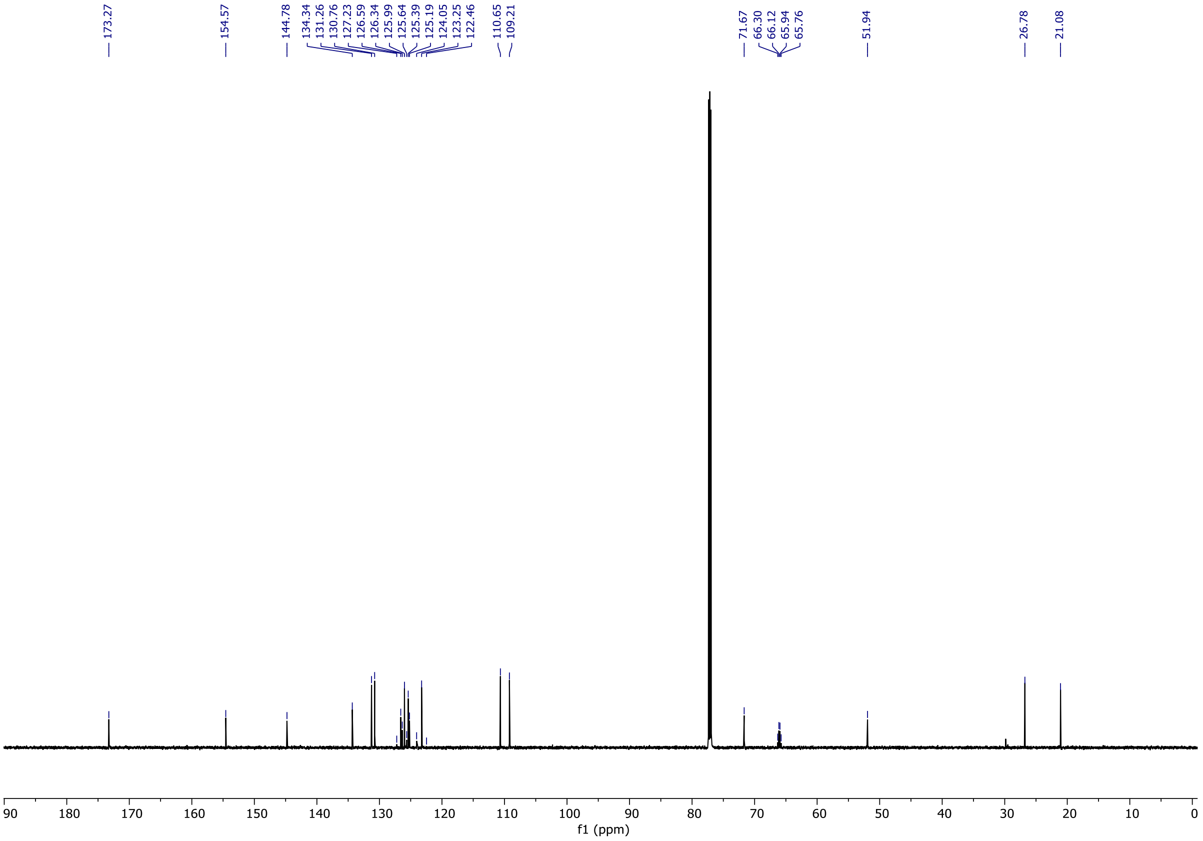

(1*R*,3*S*,3a*S*,8b*S*)-7-(*tert*-Butyl)-1'-methyl-3a-nitro-1-(trifluoromethyl)-1,2,3a,8b-tetrahydrospiro[benzofuro[2,3-c]pyrrole-3,3'-indolin]-2'-one (3n)

^{1}H NMR

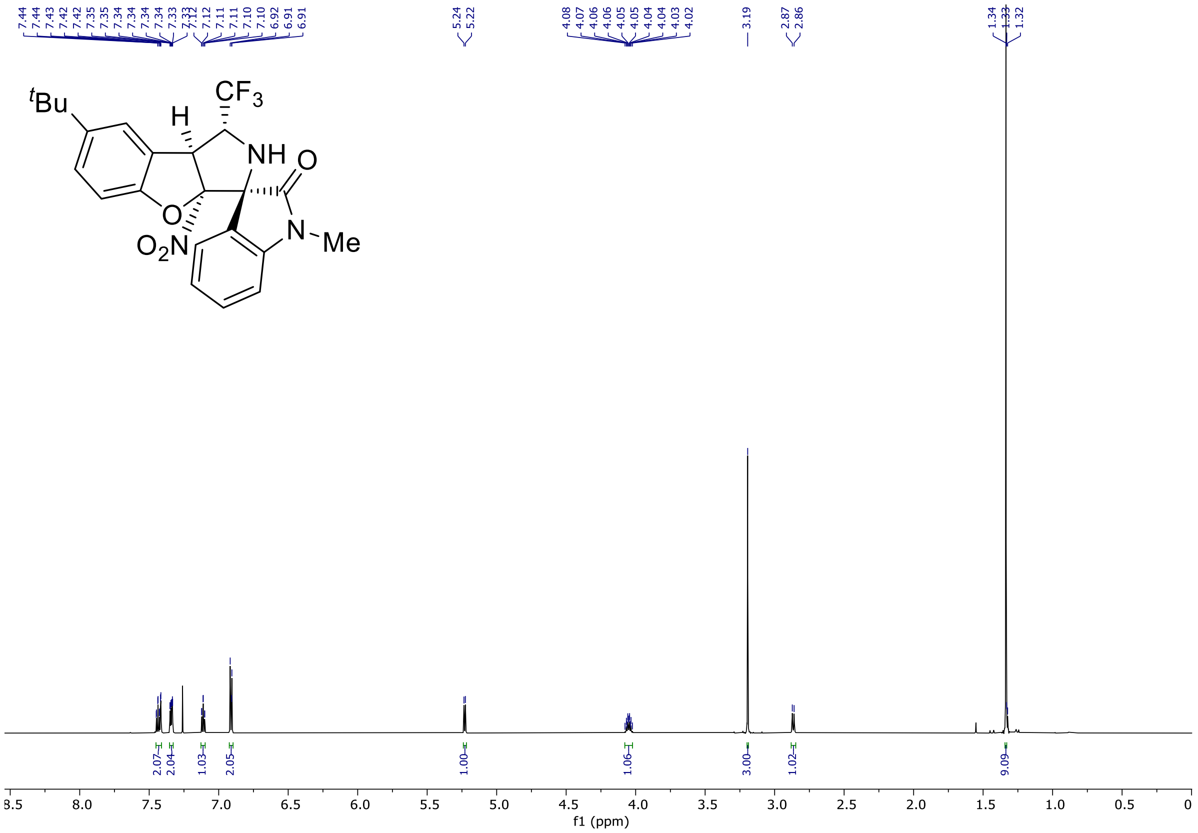

^{13}C NMR

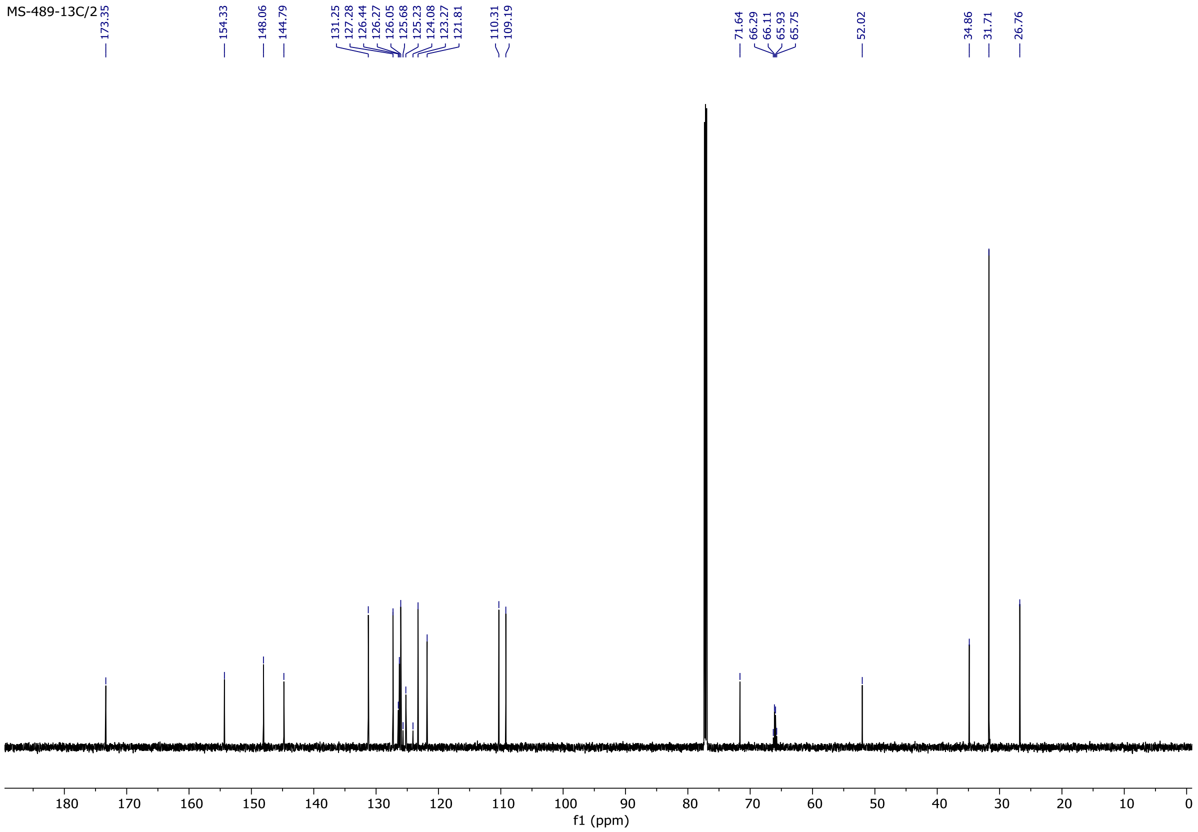

(1*R*,3*S*,3a*S*,8b*S*)-7-Chloro-1'-methyl-3a-nitro-1-(trifluoromethyl)-1,2,3a,8b-tetrahydrospiro[benzofuro[2,3-c]pyrrole-3,3'-indolin]-2'-one (3o)

^{1}H NMR

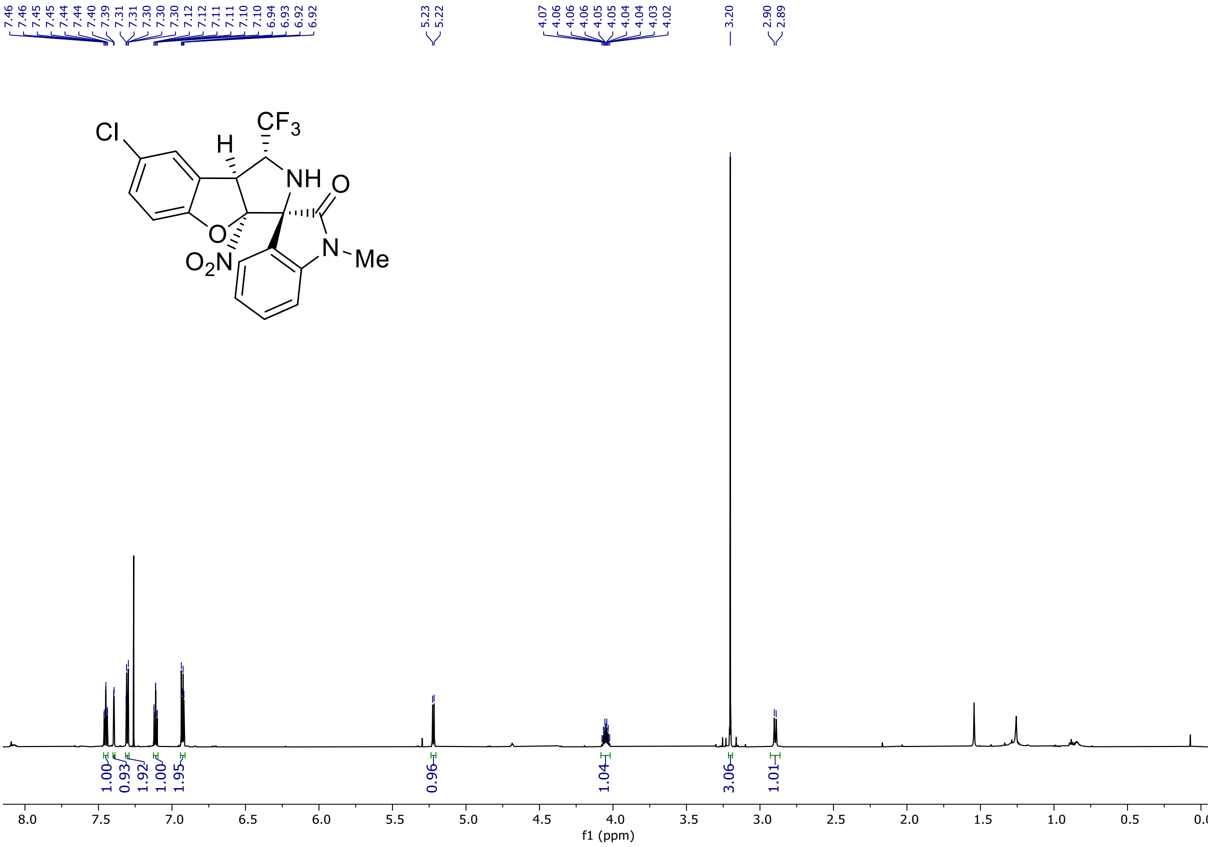

^{13}C NMR

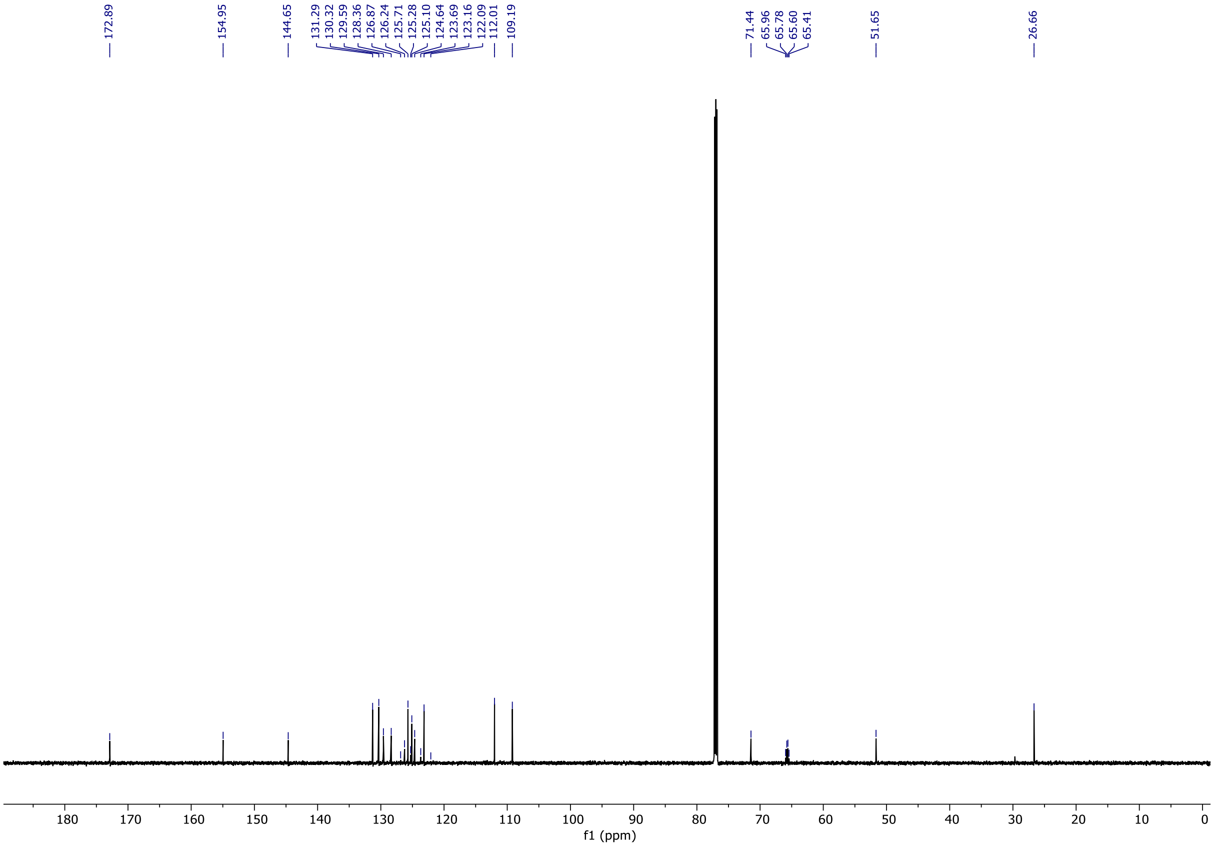

(1*R*,3*S*,3a*S*,8b*S*)-1'-Methyl-3a,7-dinitro-1-(trifluoromethyl)-1,2,3a,8b-tetrahydrospiro[benzofuro[2,3-c]pyrrole-3,3'-indolin]-2'-one (3p)

^{1}H NMR

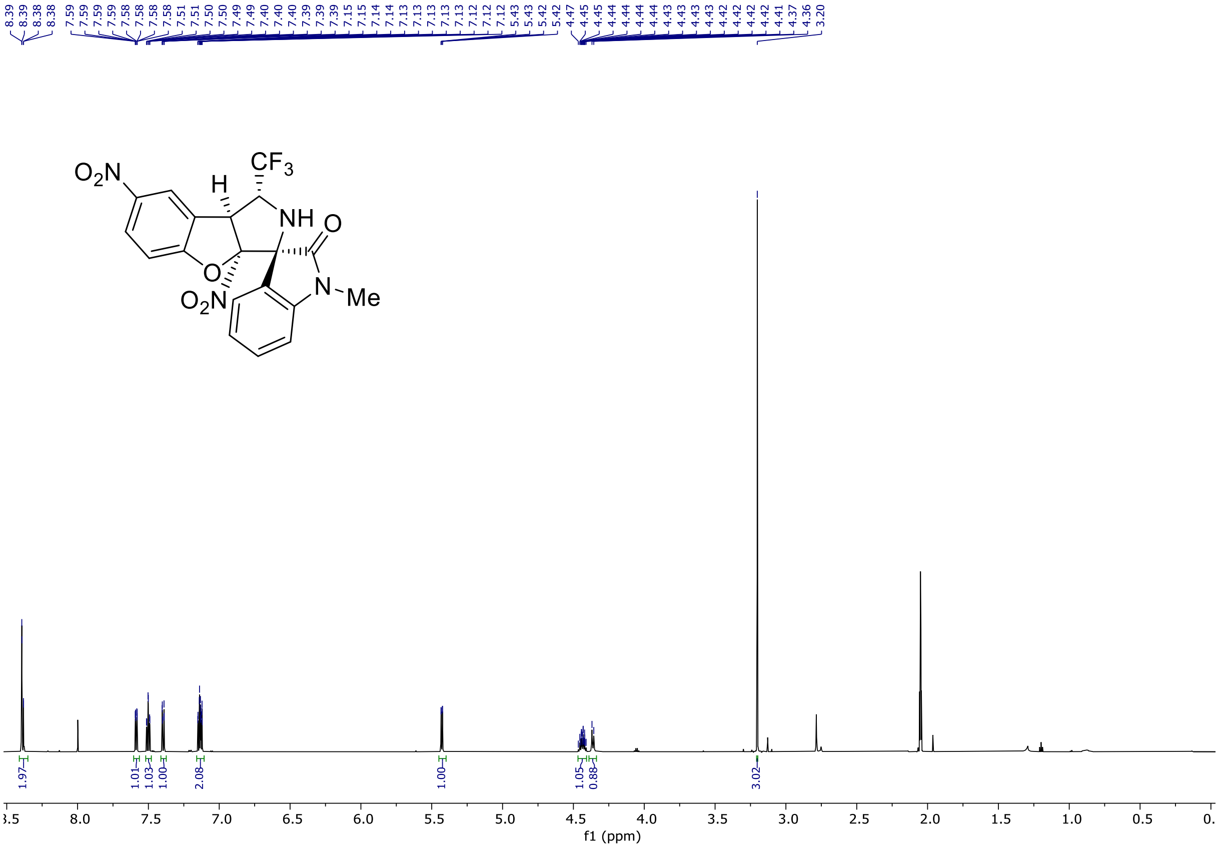

^{13}C NMR

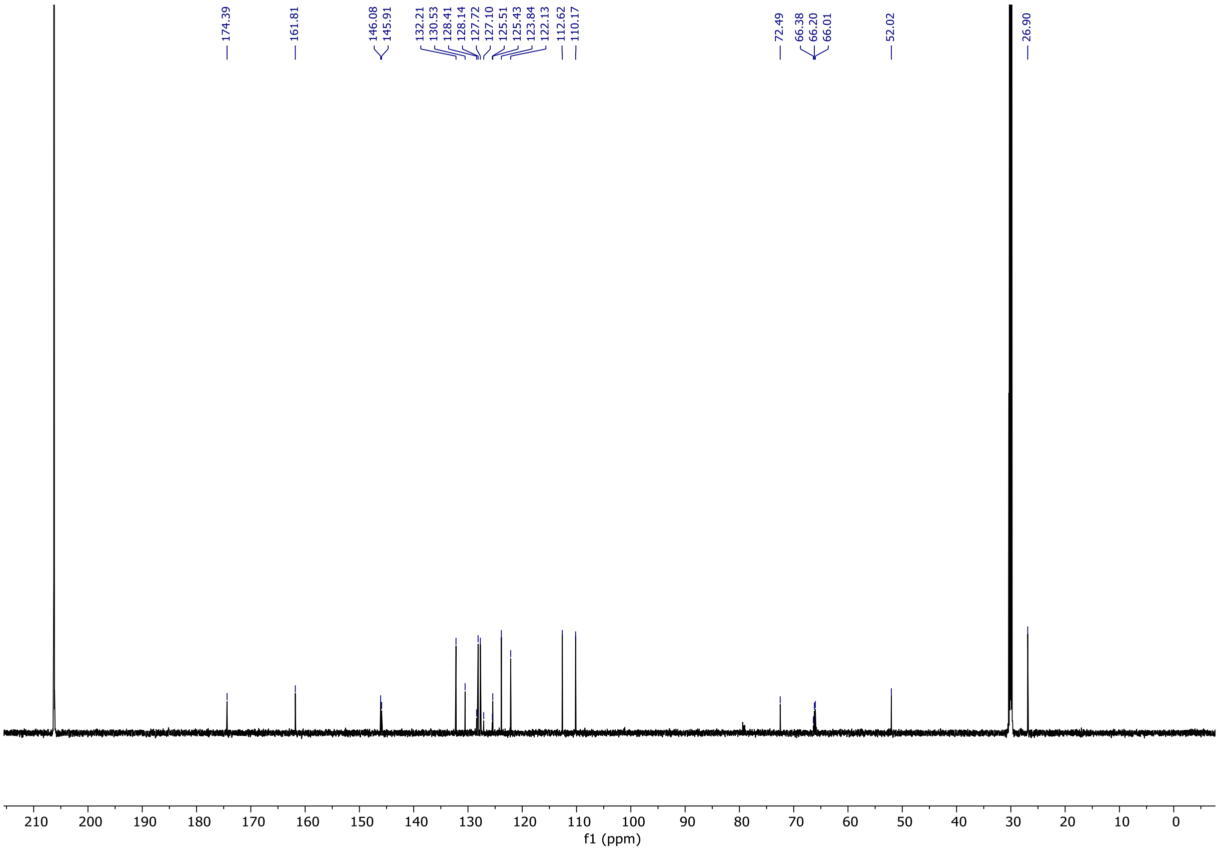

(1*R*,3*S*,3a*S*,8b*S*)-7-Bromo-1'-methyl-3a-nitro-1-(trifluoromethyl)-1,2,3a,8b-tetrahydrospiro[benzofuro[2,3-c]pyrrole-3,3'-indolin]-2'-one (3q)

^{1}H NMR

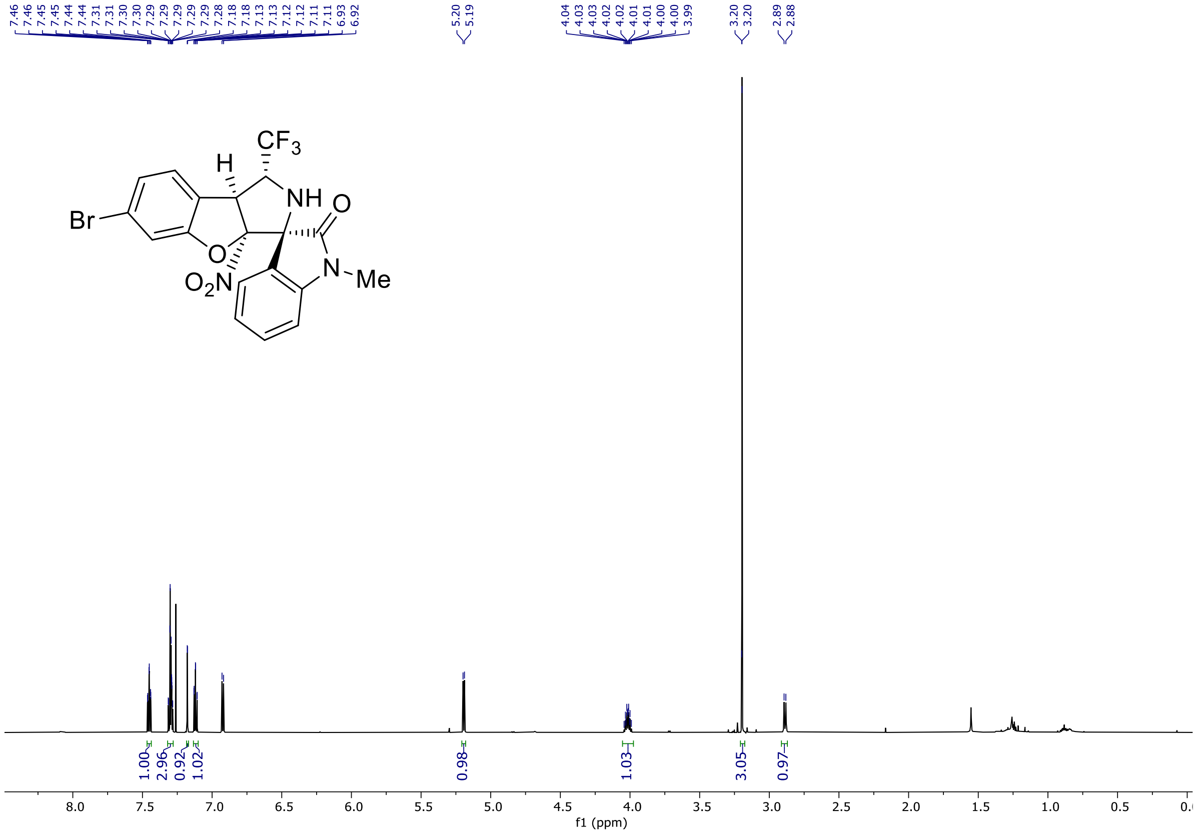

^{13}C NMR

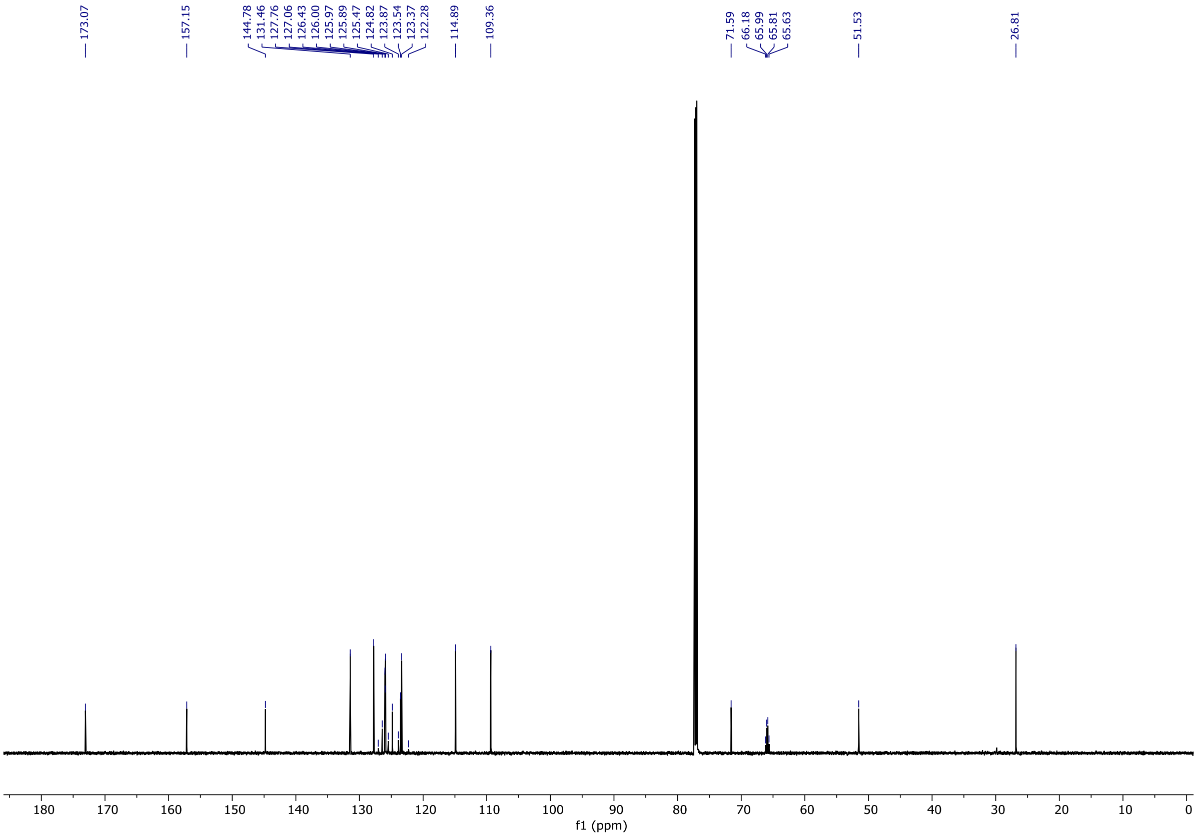

(1*R*,3*R*,3a*S*,8b*S*)-1'-Methyl-3a-nitro-1-(trifluoromethyl)-1,2,3a,8b-tetrahydrospiro[benzo[4,5]thieno[2,3-c]pyrrole-3,3'-indolin]-2'-one (3r)

^{1}HNMR

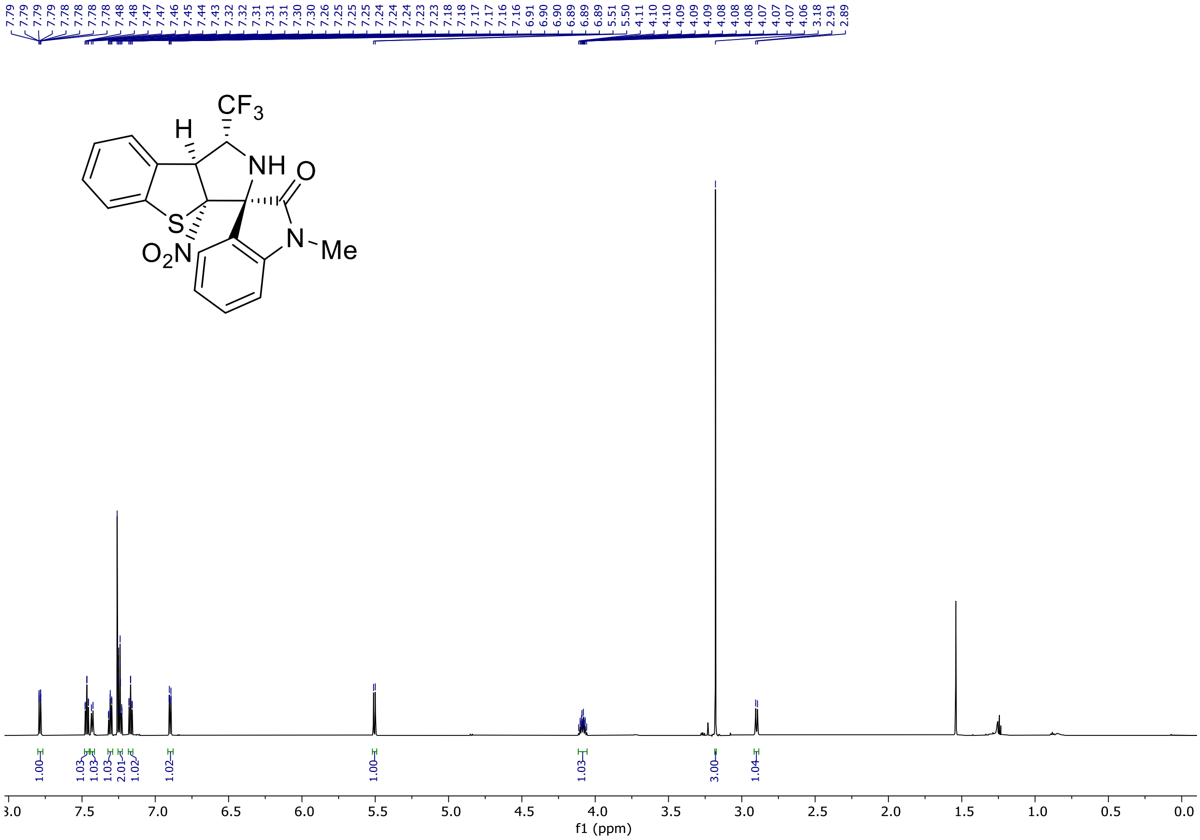

^{13}C NMR

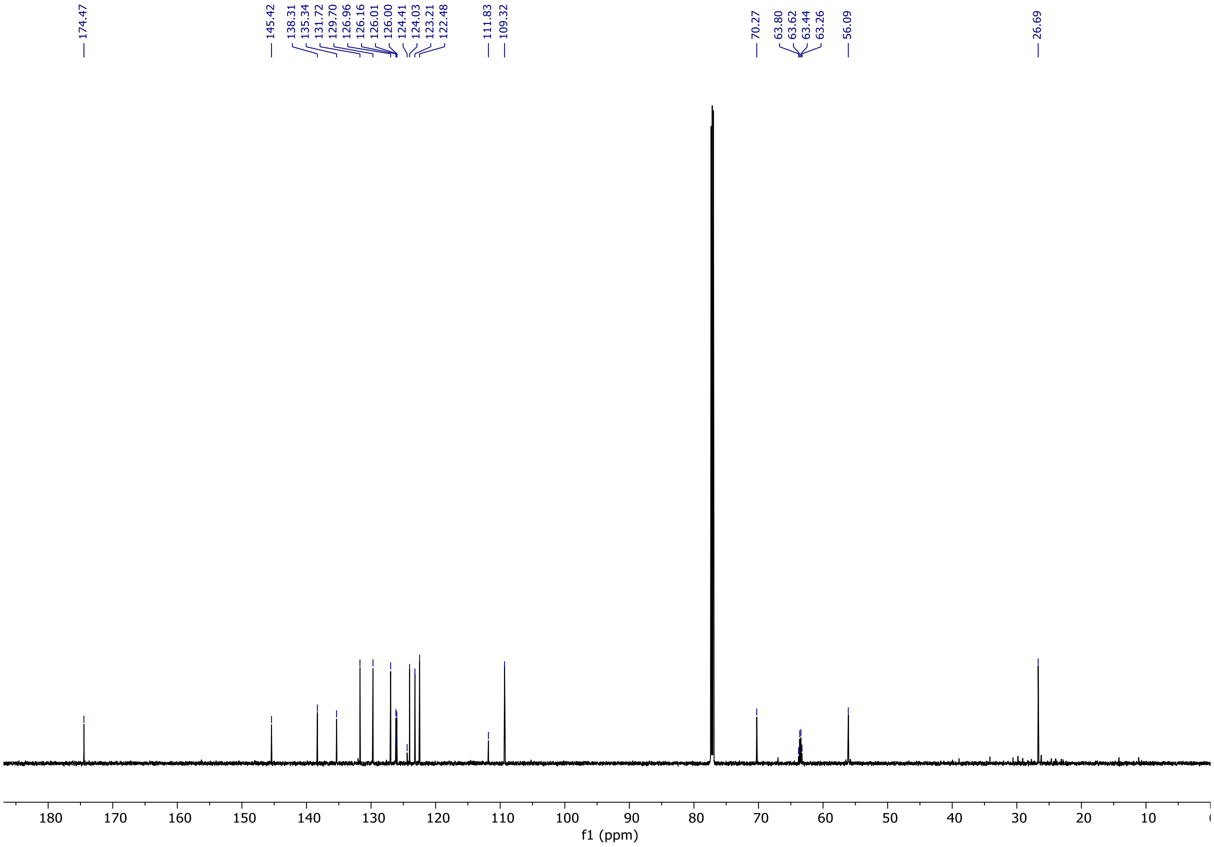

(1*R*,3*S*)-1'-Methyl-1-(trifluoromethyl)-1,2-dihydrospiro[benzofuro[2,3-c]pyrrole-3,3'-indolin]-2'-one (5)

^{1}H NMR

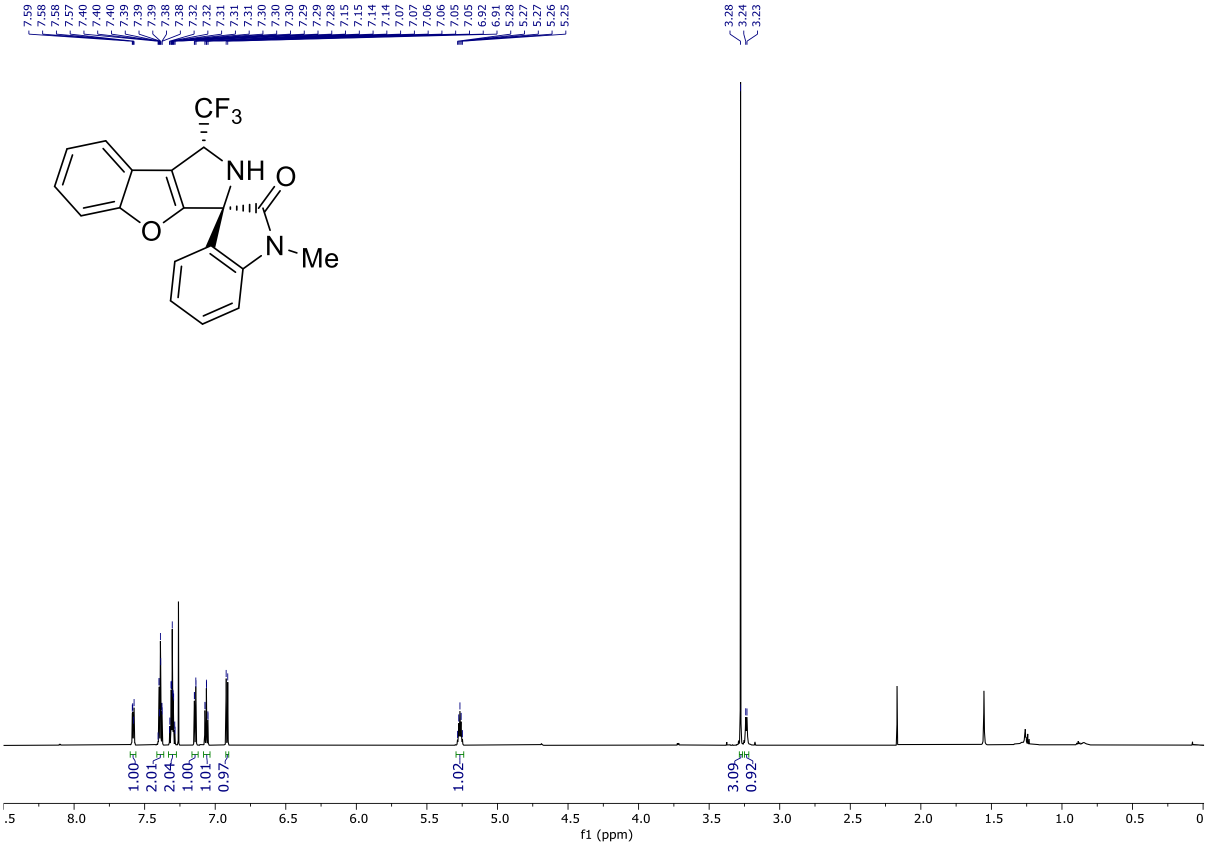

^{13}C NMR

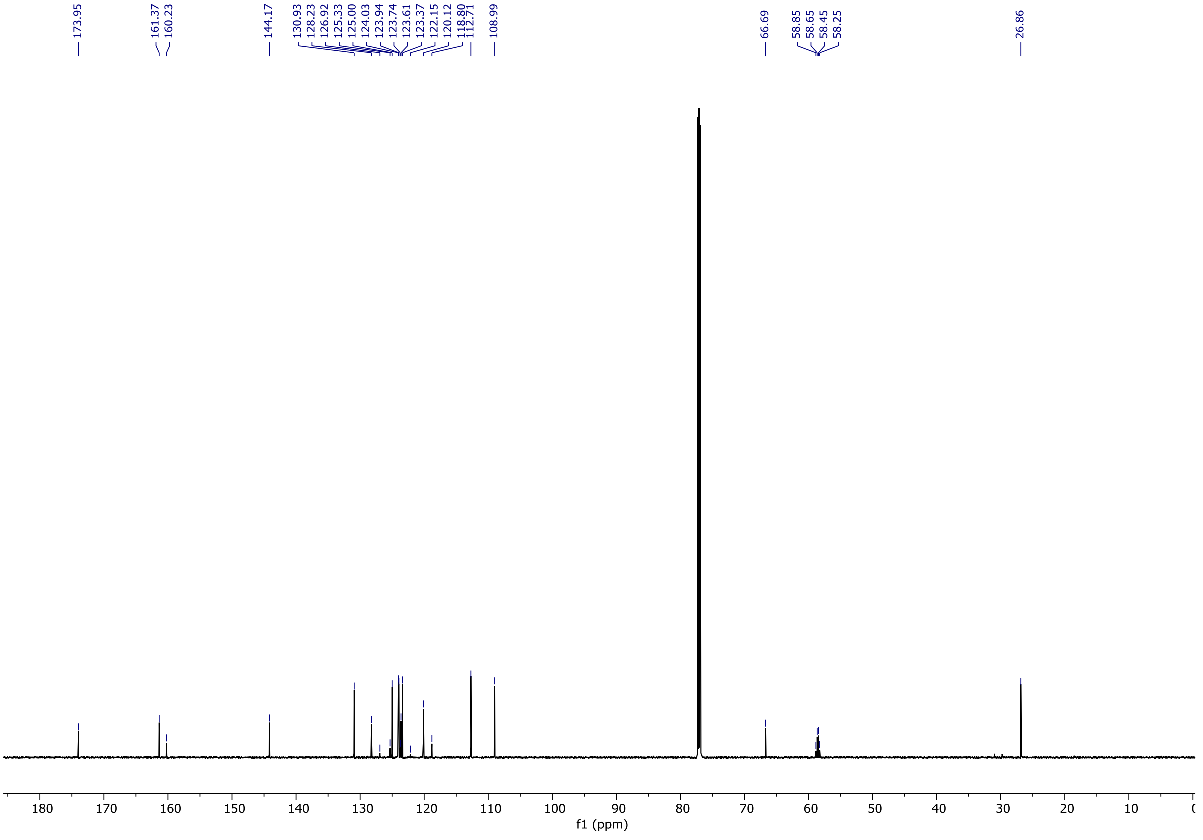

(1*R*,3*S*,3a*S*,8b*S*)-1'-Methyl-1-(trifluoromethyl)-1,2,3a,8b-tetrahydrospiro[benzofuro[2,3-c]pyrrole-3,3'-indolin]-2'-one (6)

^{1}H NMR

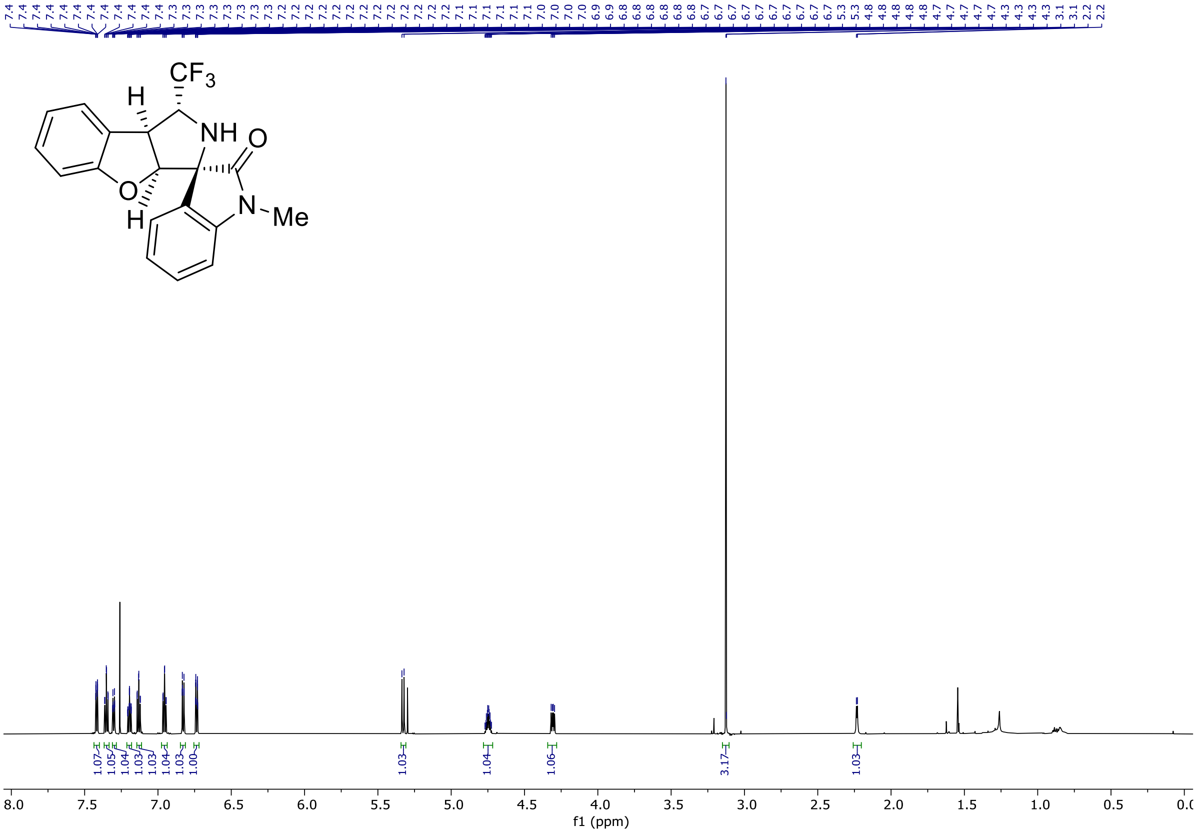

^{13}C NMR

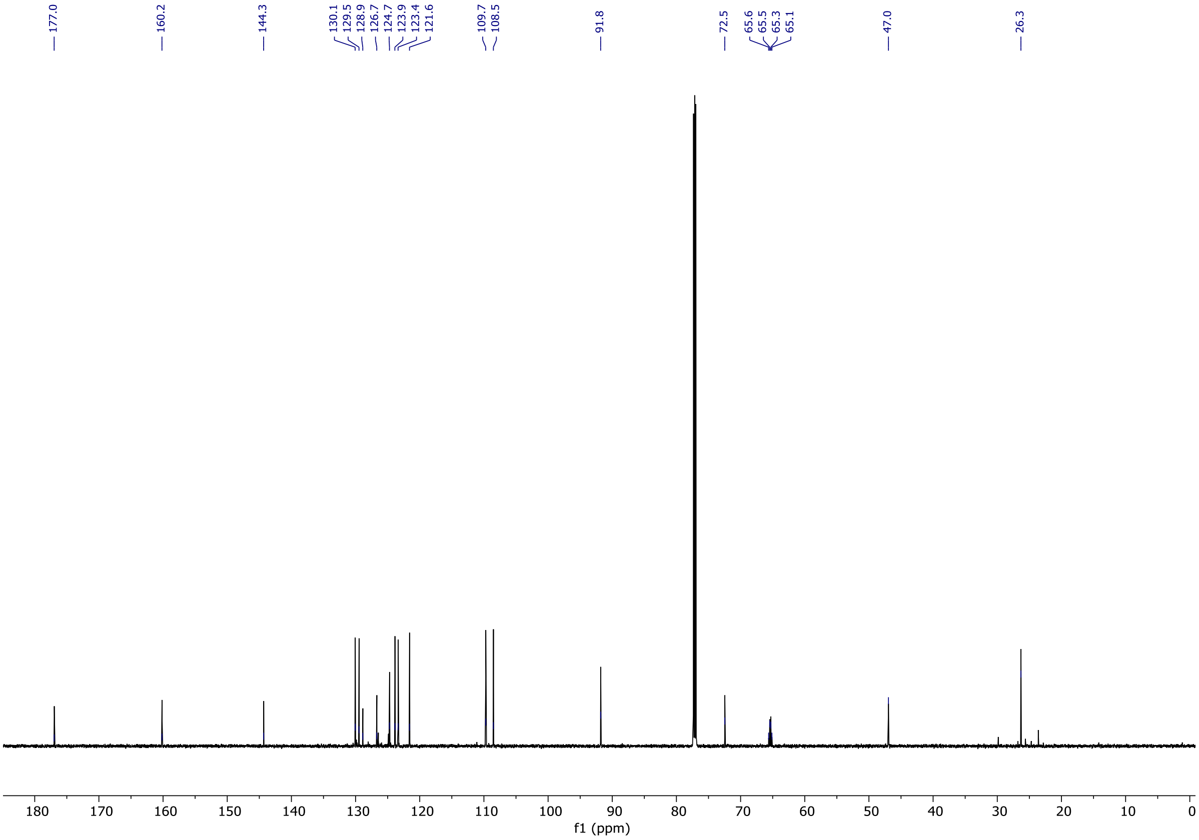

7. UPC^2 trace

(1*R*,3*S*,3a*S*,8b*S*)-1'-Methyl-3a-nitro-1-(trifluoromethyl)-1,2,3a,8b-tetrahydrospiro[benzofuro[2,3-c]pyrrole-3,3'-indolin]-2'-one (3a)

Racemic sample

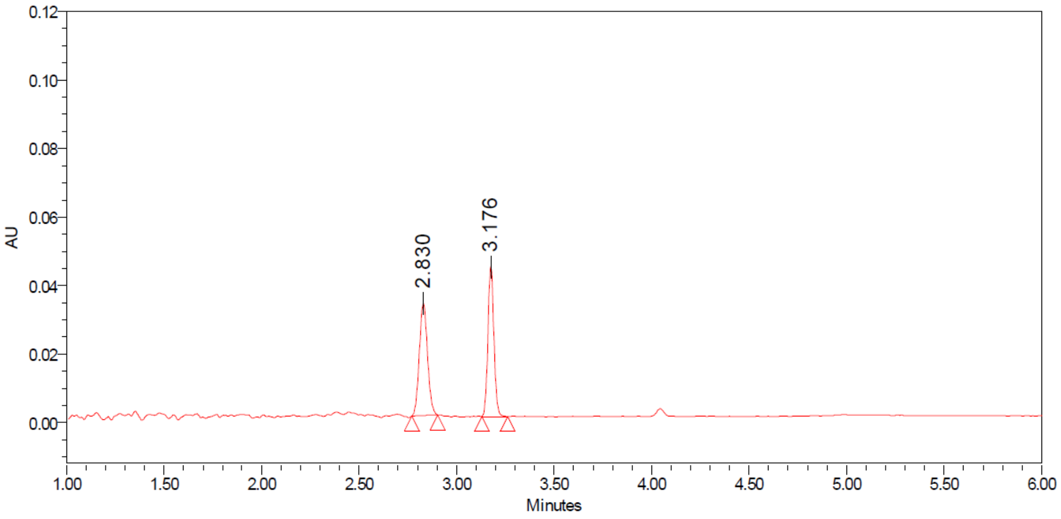

Peak Results

	RT
1	2.830
2	3.176

Enantiomerically enriched sample

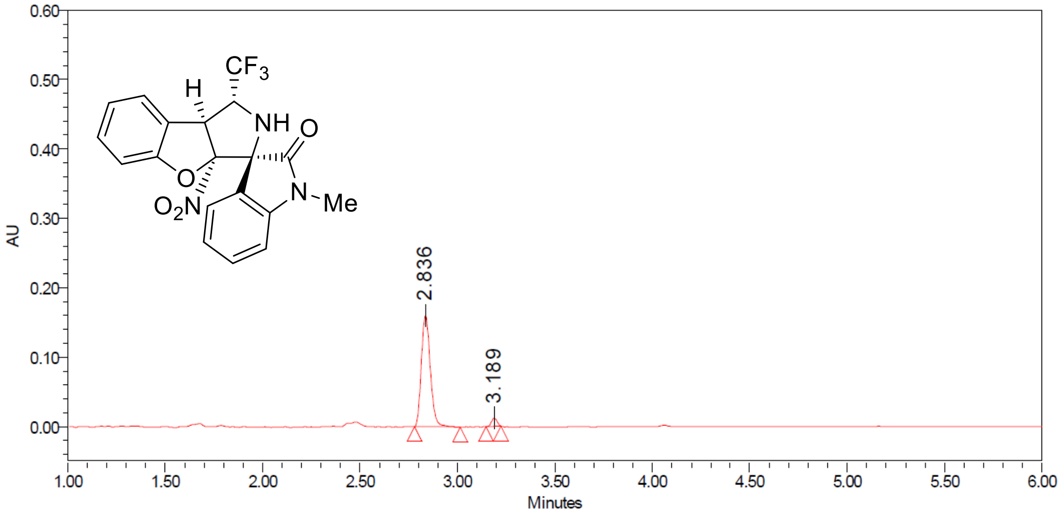

Peak Results

	RT	% Area
1	2.836	95.54
2	3.189	4.46

(1*R*,3*S*,3a*S*,8b*S*)-1'-Allyl-3a-nitro-1-(trifluoromethyl)-1,2,3a,8b-tetrahydrospiro[benzofuro[2,3-c]pyrrole-3,3'-indolin]-2'-one (3b)

Racemic sample

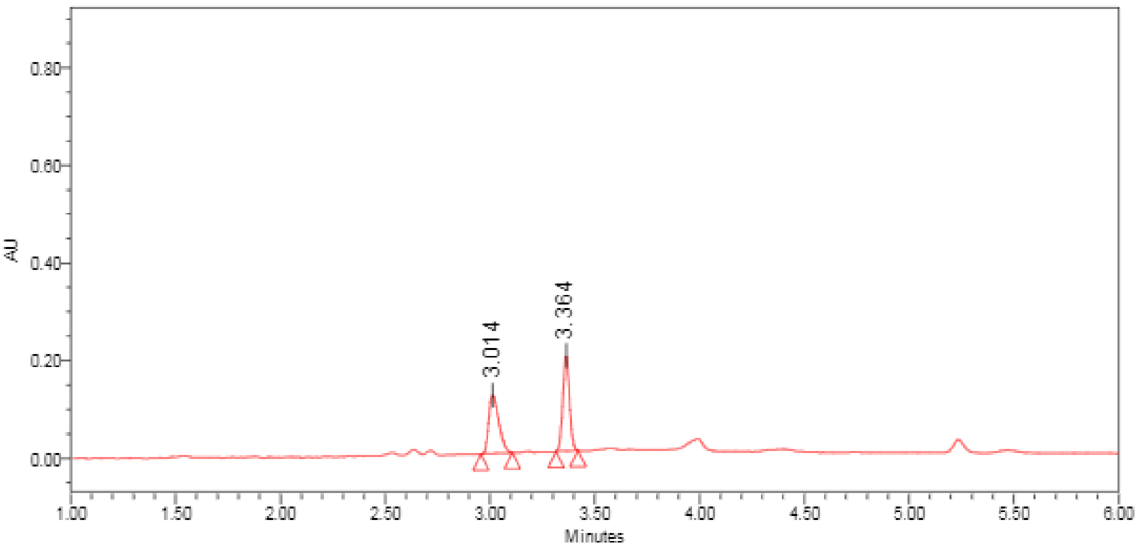

Peak Results

	RT
1	3.014
2	3.364

Enantiomerically enriched sample

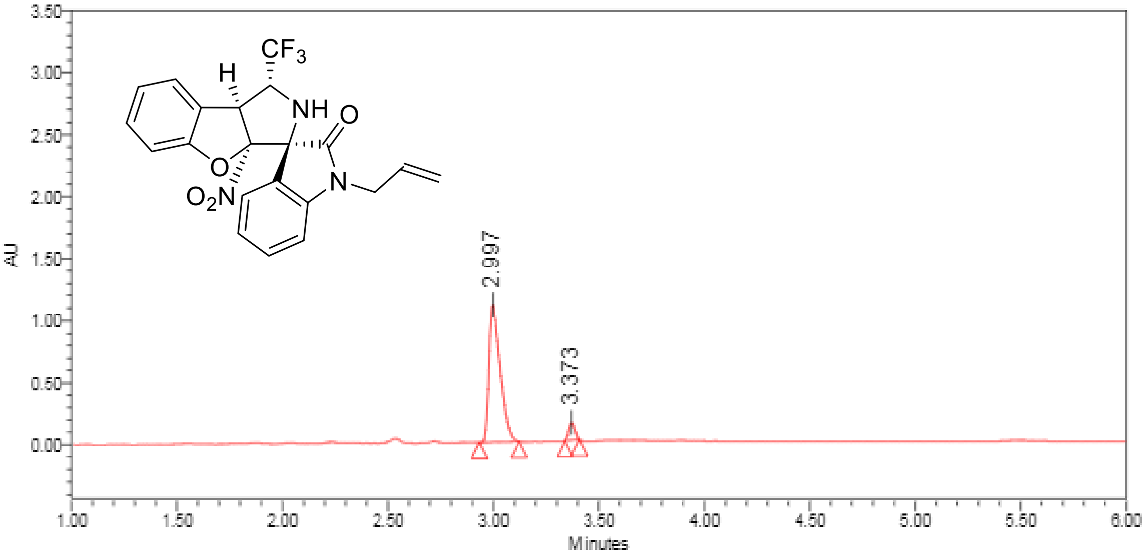

Peak Results

	RT	% Area
1	2.997	93.94
2	3.373	6.06

(1*R*,3*S*,3a*S*,8b*S*)-1'-Benzyl-3a-nitro-1-(trifluoromethyl)-1,2,3a,8b-tetrahydrospiro[benzofuro[2,3-c]pyrrole-3,3'-indolin]-2'-one (3c)

Racemic sample

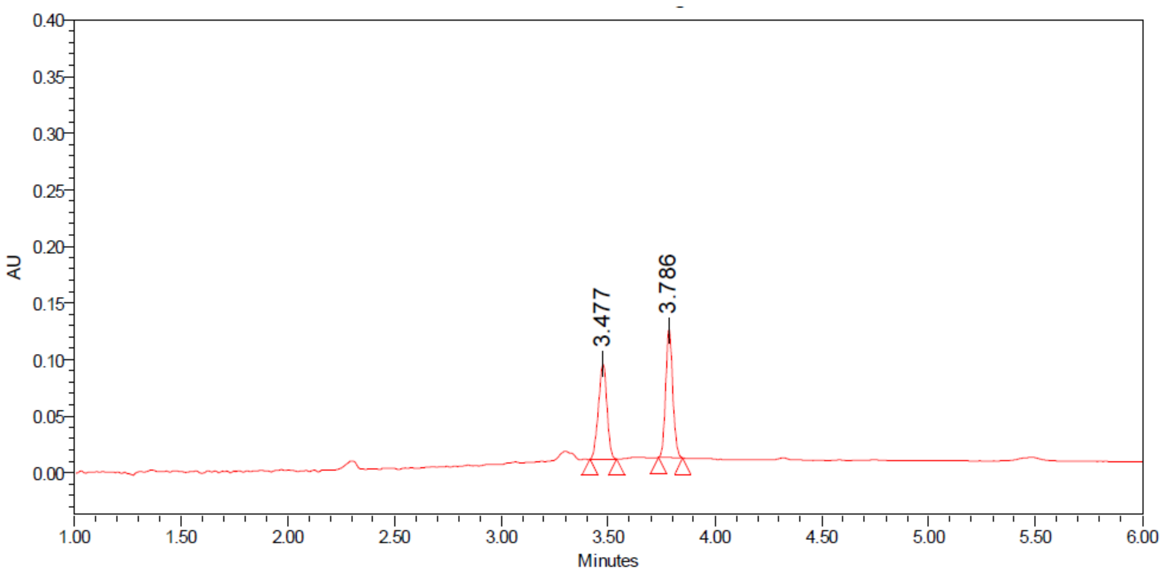

Peak Results

	RT
1	3.477
2	3.786

Enantiomerically enriched sample

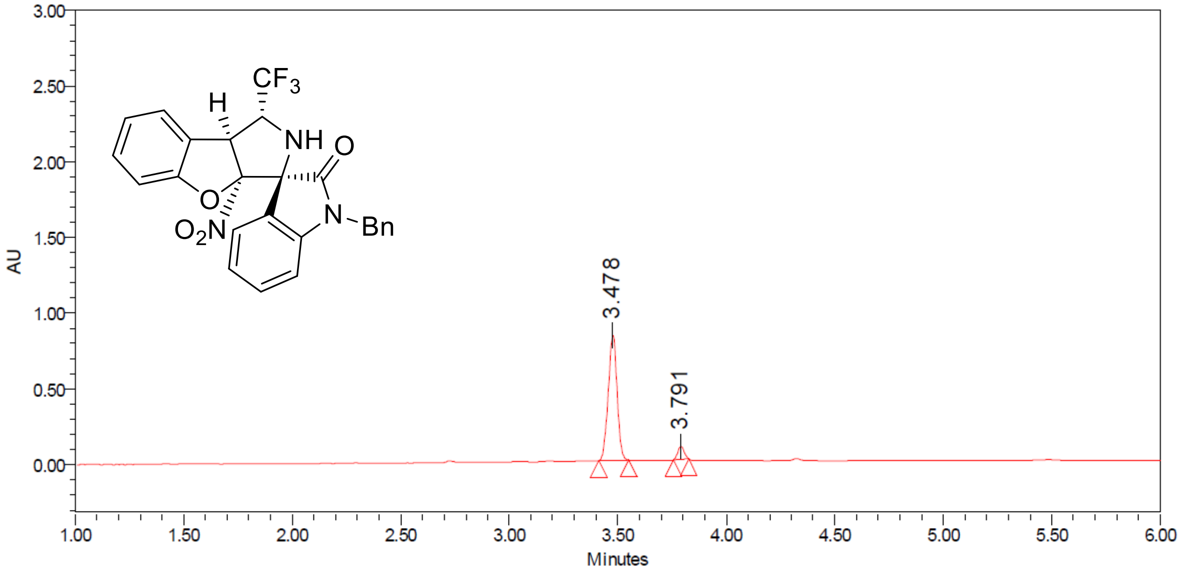

Peak Results

	RT	% Area
1	3.478	93.01
2	3.791	6.99

(1*R*,3*S*,3a*S*,8b*S*)-3a-Nitro-1-(trifluoromethyl)-1,2,3a,8b-tetrahydrospiro[benzofuro[2,3-c]pyrrole-3,3'-indolin]-2'-one (3d)

Racemic sample

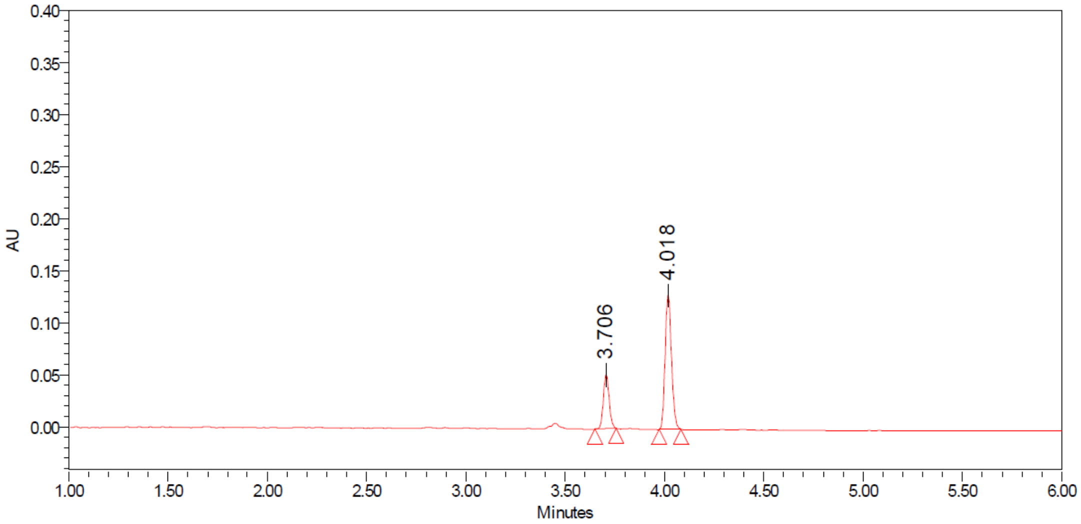

Peak Results

	RT
1	3.706
2	4.018

Enantiomerically enriched sample

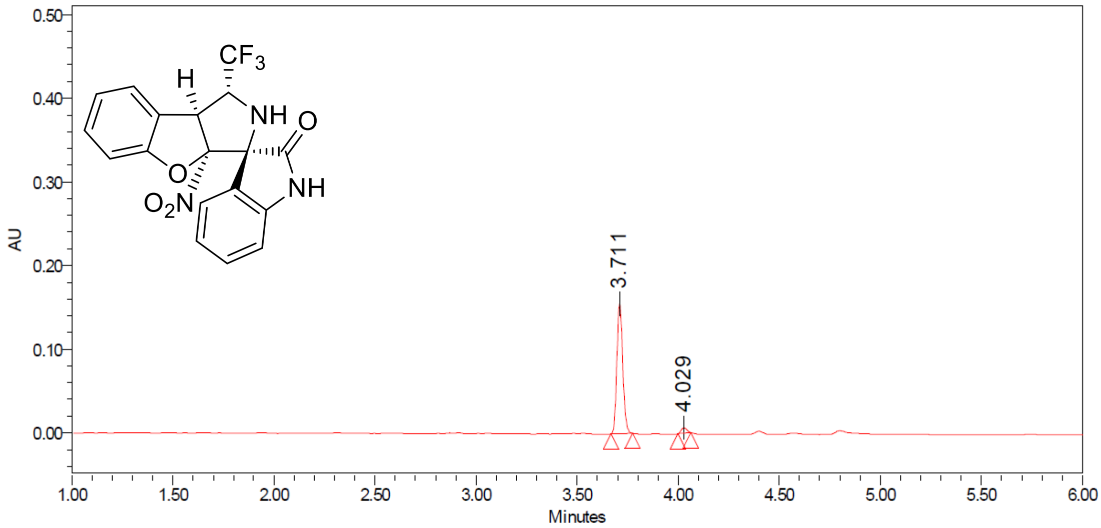

Peak Results

	RT	% Area
1	3.711	96.20
2	4.029	3.80

(1*R*,3*S*,3a*S*,8b*S*)-5'-Methoxy-3a-nitro-1-(trifluoromethyl)-1,2,3a,8b-tetrahydrospiro[benzofuro[2,3-c]pyrrole-3,3'-indolin]-2'-one (3e)

Racemic sample

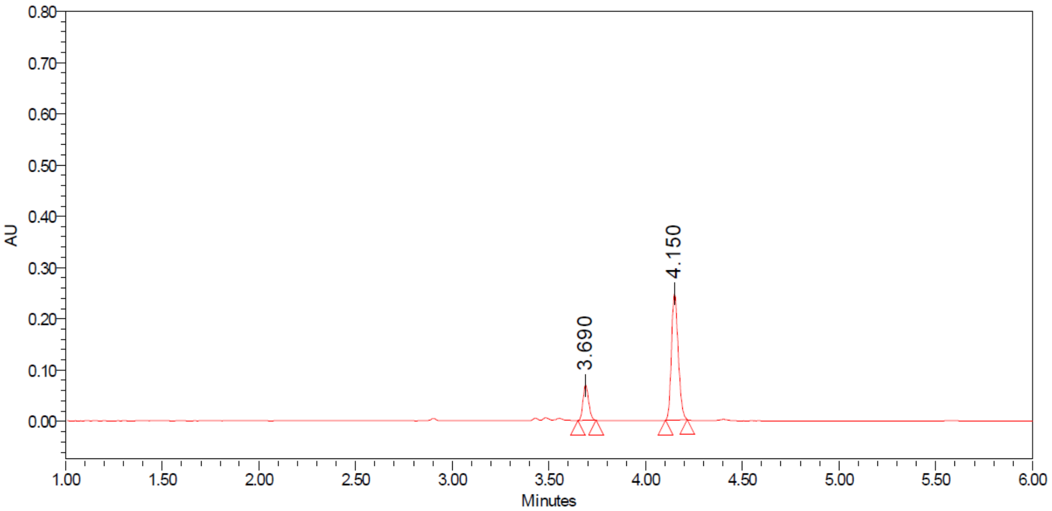

Peak Results

	RT
1	3.690
2	4.150

Enantiomerically enriched sample

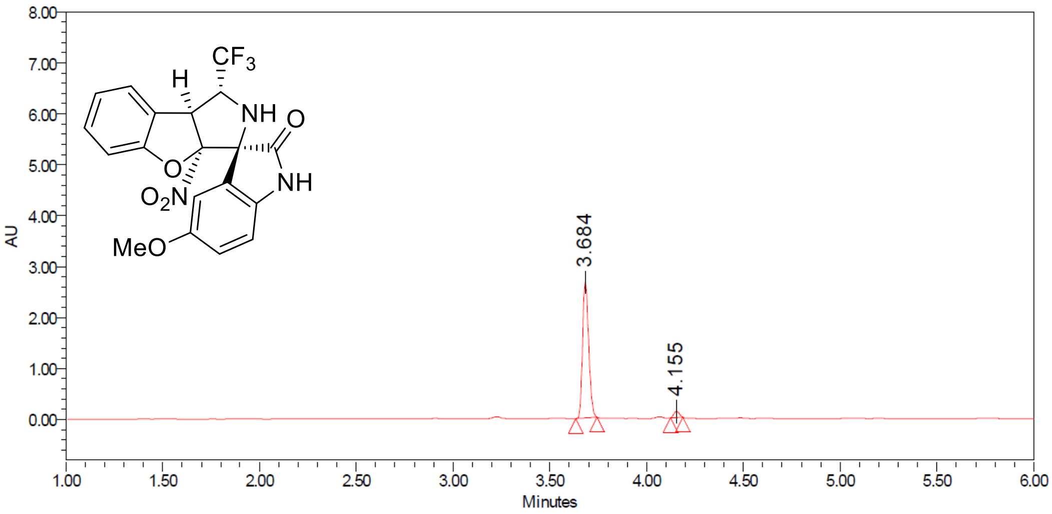

Peak Results

	RT	% Area
1	3.684	96.04
2	4.155	3.96

(1*R*,3*S*,3a*S*,8b*S*)-5'-Methyl-3a-nitro-1-(trifluoromethyl)-1,2,3a,8b-tetrahydrospiro[benzofuro[2,3-c]pyrrole-3,3'-indolin]-2'-one (3f)

Racemic sample

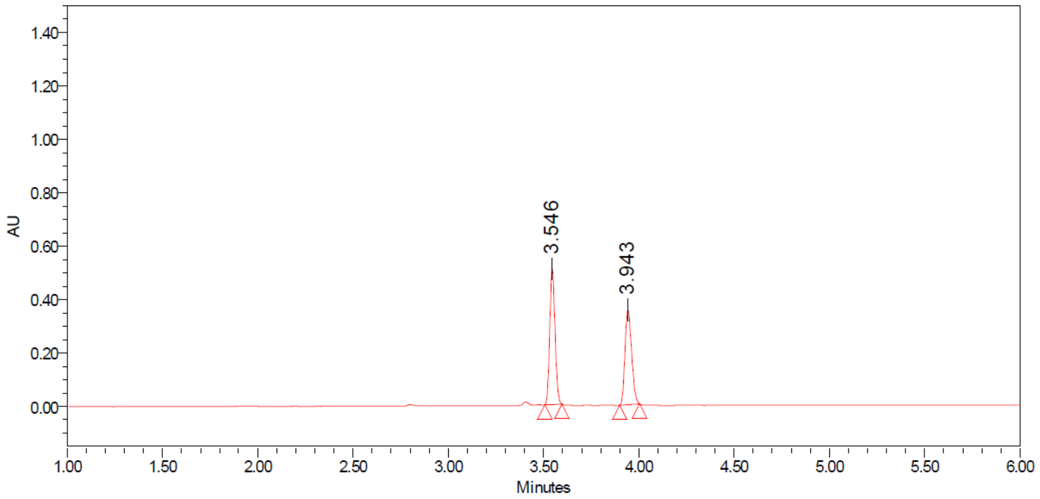

Peak Results

	RT
1	3.546
2	3.943

Enantiomerically enriched sample

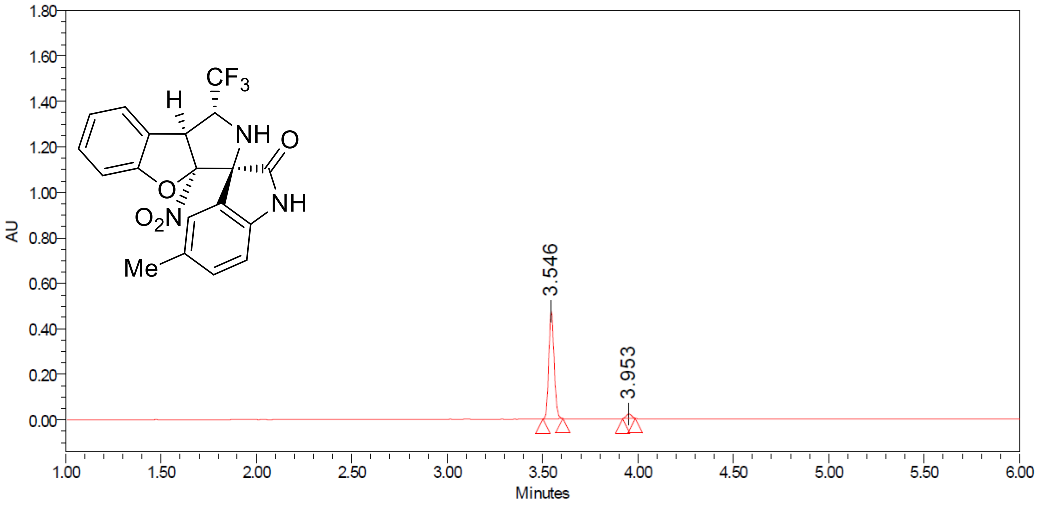

Peak Results

	RT	% Area
1	3.546	95.71
2	3.953	4.29

(1*R*,3*S*,3a*S*,8b*S*)-5'-Bromo-3a-nitro-1-(trifluoromethyl)-1,2,3a,8b-tetrahydrospiro[benzofuro[2,3-c]pyrrole-3,3'-indolin]-2'-one (3g)

Racemic sample

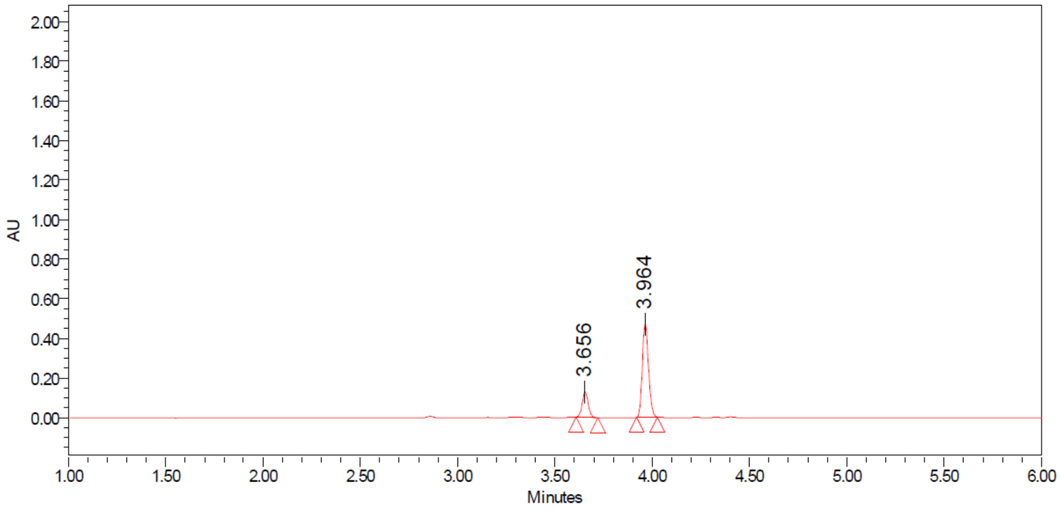

Peak Results

	RT
1	3.656
2	3.964

Enantiomerically enriched sample

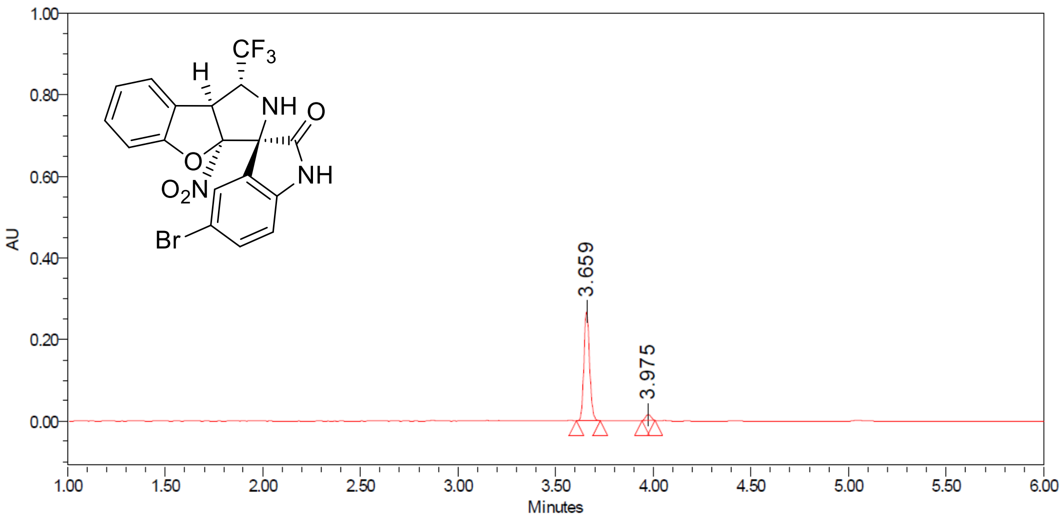

Peak Results

	RT	% Area
1	3.659	94.89
2	3.975	5.11

(1*R*,3*S*,3a*S*,8b*S*)-5'-Chloro-3a-nitro-1-(trifluoromethyl)-1,2,3a,8b-tetrahydrospiro[benzofuro[2,3-c]pyrrole-3,3'-indolin]-2'-one (3h)

Racemic sample

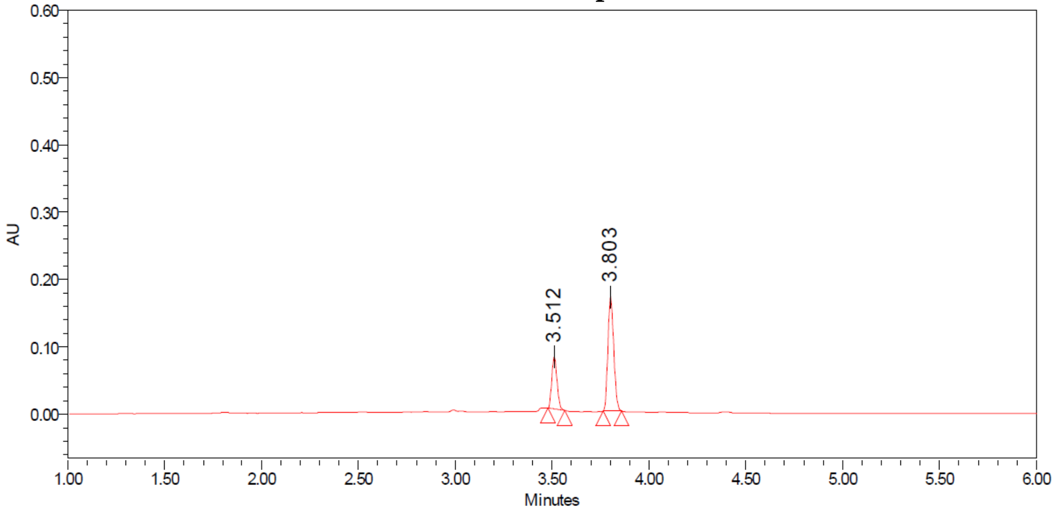

Peak Results

	RT
1	3.512
2	3.803

Enantiomerically enriched sample

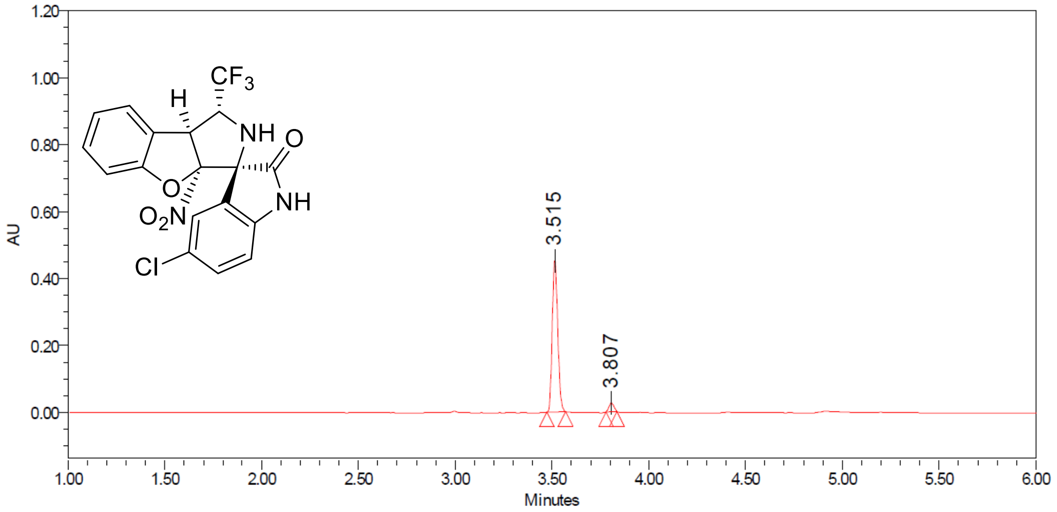

Peak Results

	RT	% Area
1	3.515	95.14
2	3.807	4.86

(1*R*,3*S*,3a*S*,8b*S*)-7'-Chloro-3a-nitro-1-(trifluoromethyl)-1,2,3a,8b-tetrahydrospiro[benzofuro[2,3-c]pyrrole-3,3'-indolin]-2'-one (3i)

Racemic sample

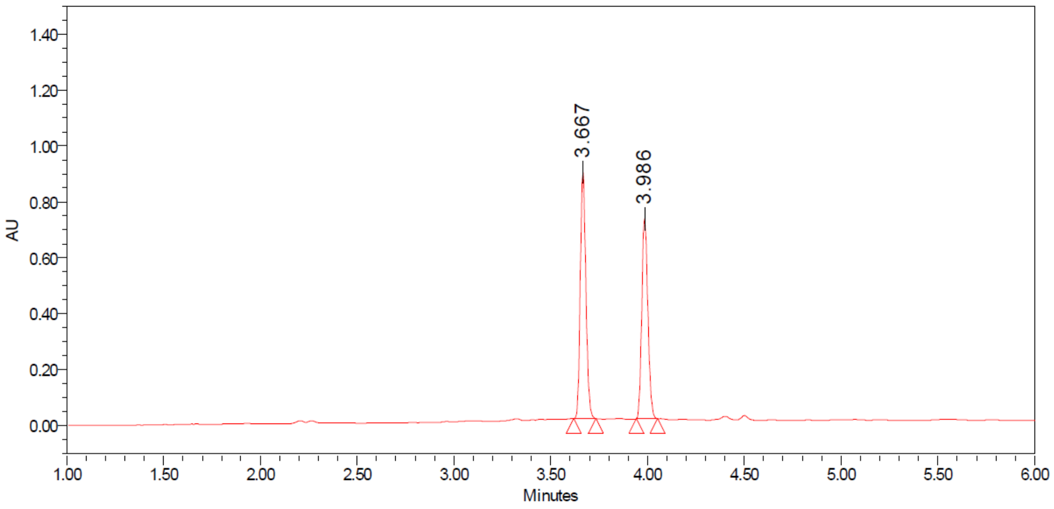

Peak Results

	RT
1	3.667
2	3.986

Enantiomerically enriched sample

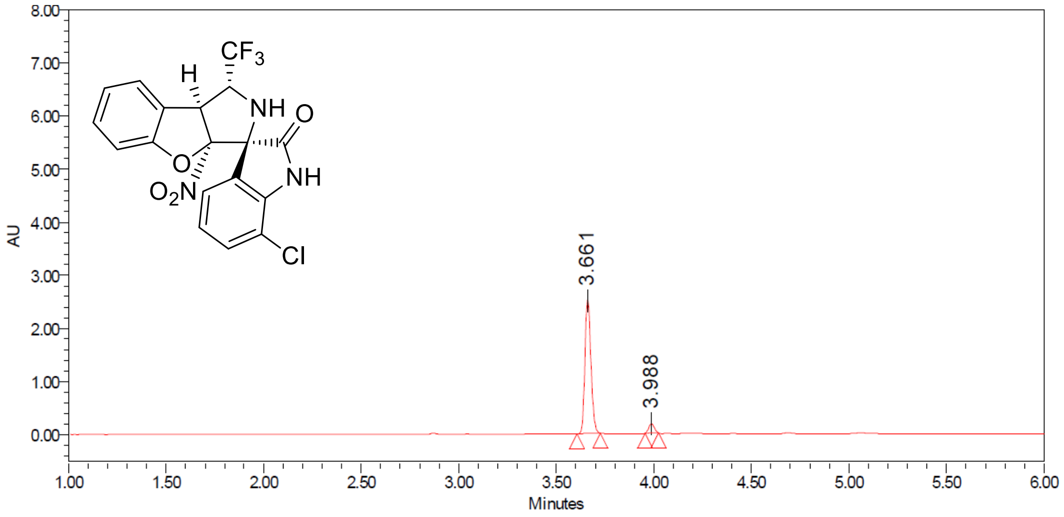

Peak Results

	RT	% Area
1	3.661	94.03
2	3.988	5.97

(1*R*,3*S*,3a*S*,8b*S*)-3a,5'-Dinitro-1-(trifluoromethyl)-1,2,3a,8b-tetrahydrospiro[benzofuro[2,3-c]pyrrole-3,3'-indolin]-2'-one (3j)

Racemic sample

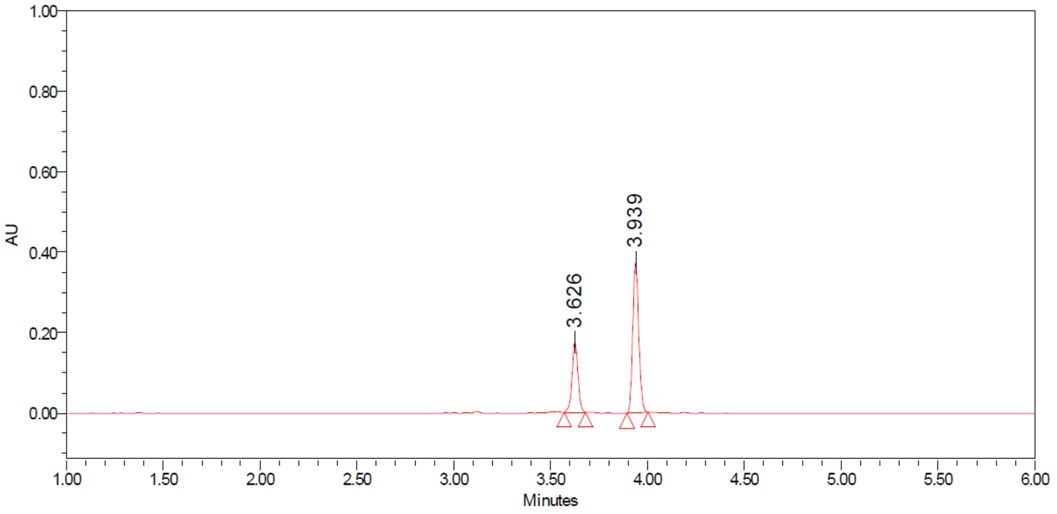

Peak Results

	RT
1	3.626
2	3.939

Enantiomerically enriched sample

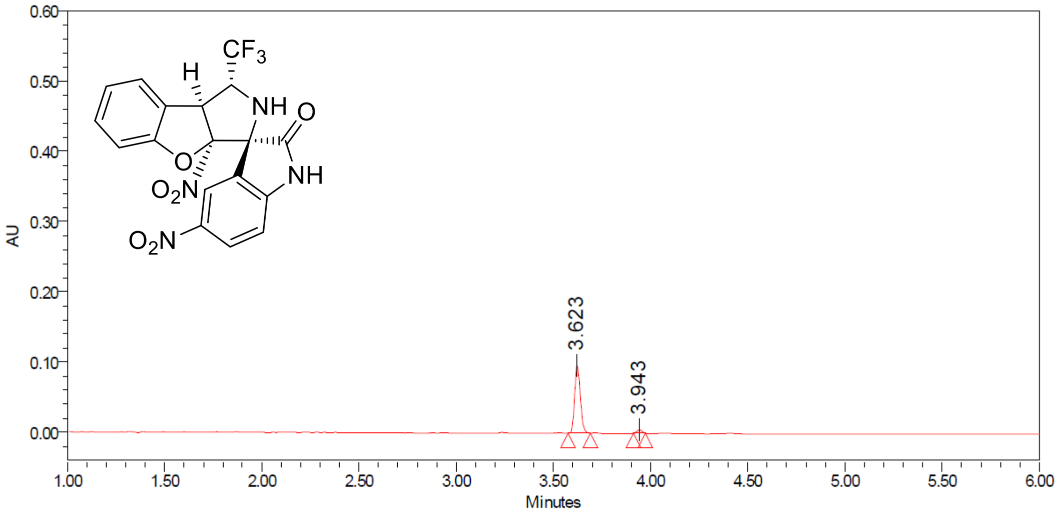

Peak Results

	RT	% Area
1	3.623	95.54
2	3.943	4.46

(1*R*,3*S*,3a*S*,8b*S*)-5',7'-Dibromo-3a-nitro-1-(trifluoromethyl)-1,2,3a,8b-tetrahydrospiro[benzofuro[2,3-c]pyrrole-3,3'-indolin]-2'-one (3k)

Racemic sample

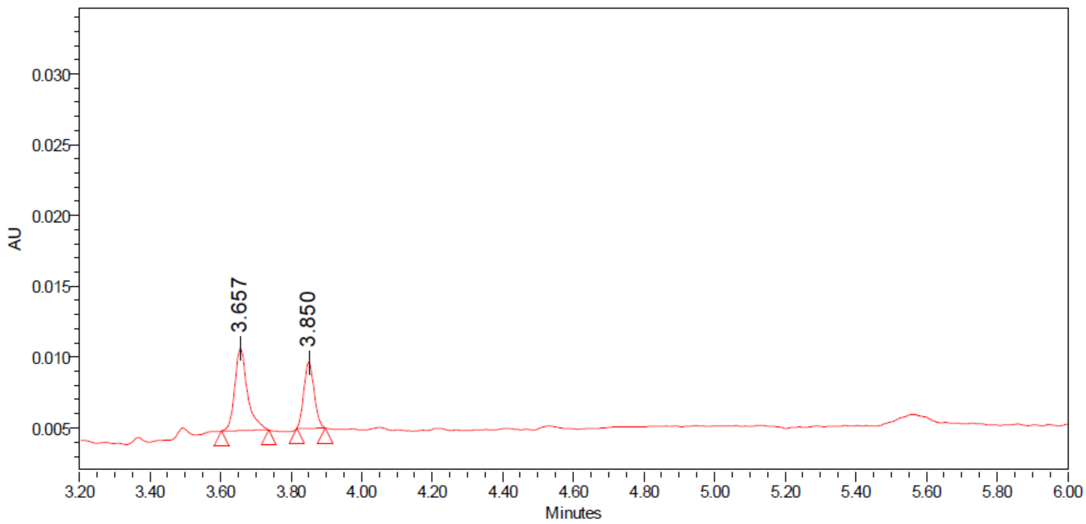

Peak Results

	RT
1	3.657
2	3.850

Enantiomerically enriched sample

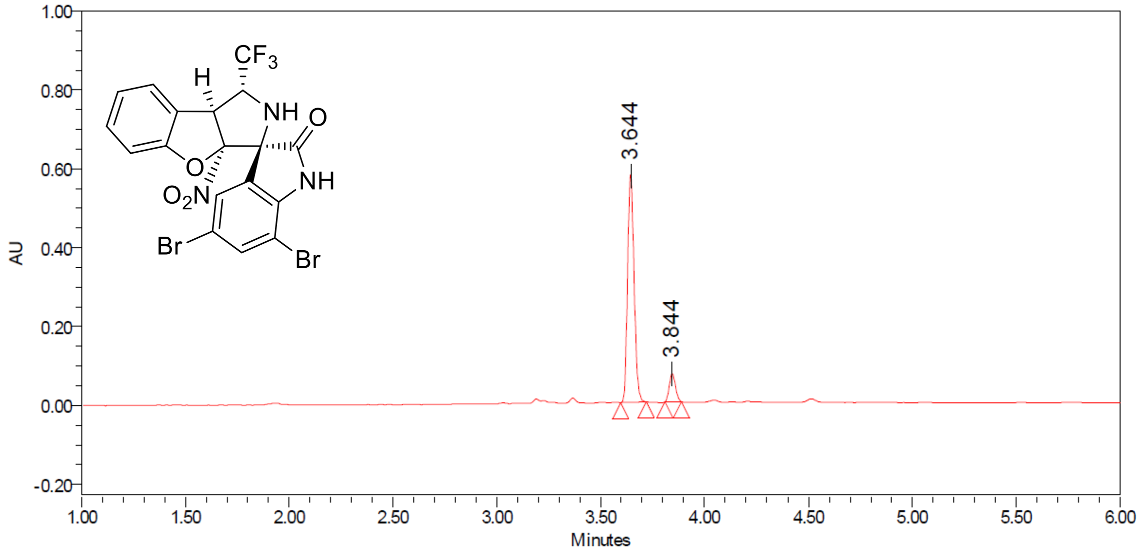

Peak Results

	RT	% Area
1	3.644	89.28
2	3.844	10.72

(1*R*,3*S*,3a*S*,8b*S*)-7-Methoxy-1'-methyl-3a-nitro-1-(trifluoromethyl)-1,2,3a,8b-tetrahydrospiro[benzofuro[2,3-c]pyrrole-3,3'-indolin]-2'-one (3l)

Racemic sample

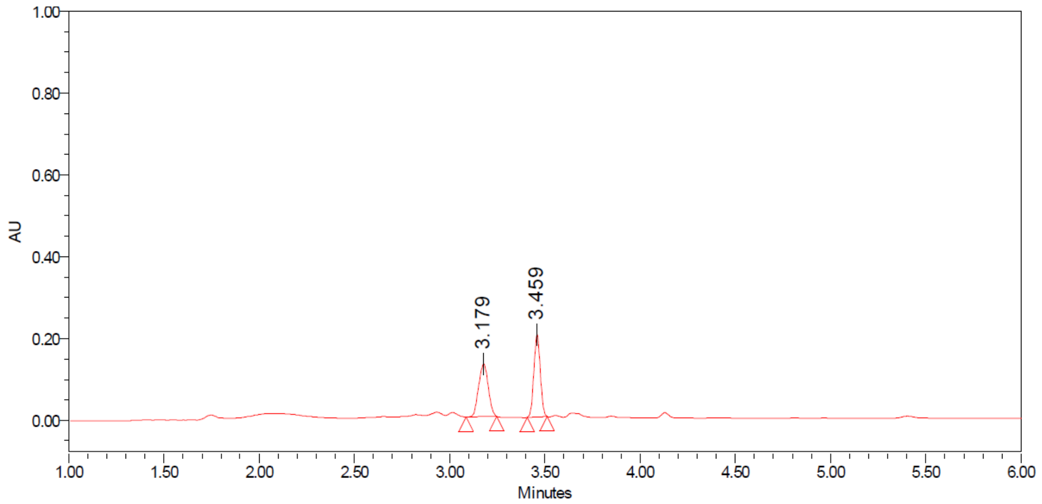

Peak Results

	RT
1	3.179
2	3.459

Enantiomerically enriched sample

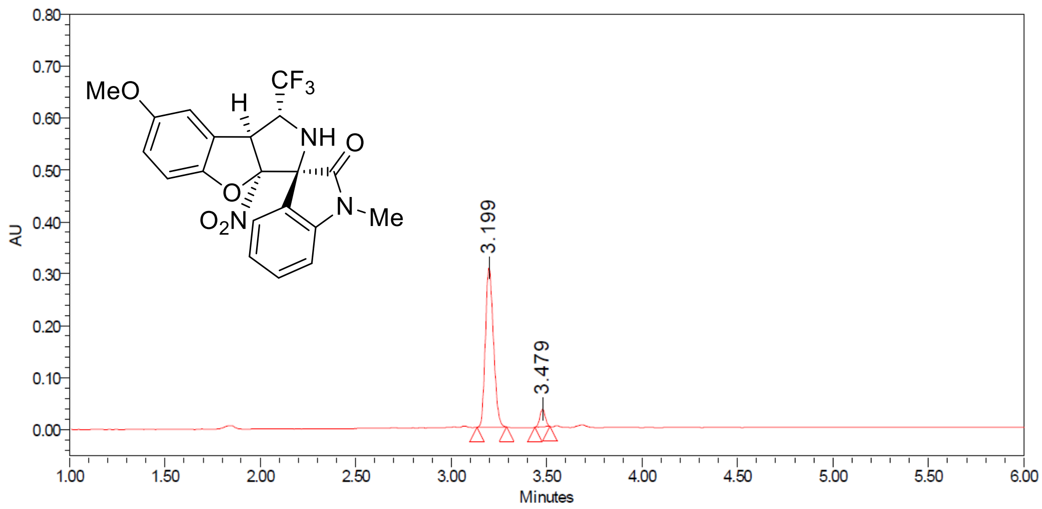

Peak Results

	RT	% Area
1	3.199	93.02
2	3.479	6.98

(1*R*,3*S*,3a*S*,8b*S*)-1',7-Dimethyl-3a-nitro-1-(trifluoromethyl)-1,2,3a,8b-tetrahydrospiro[benzofuro[2,3-c]pyrrole-3,3'-indolin]-2'-one (3m)

Racemic sample

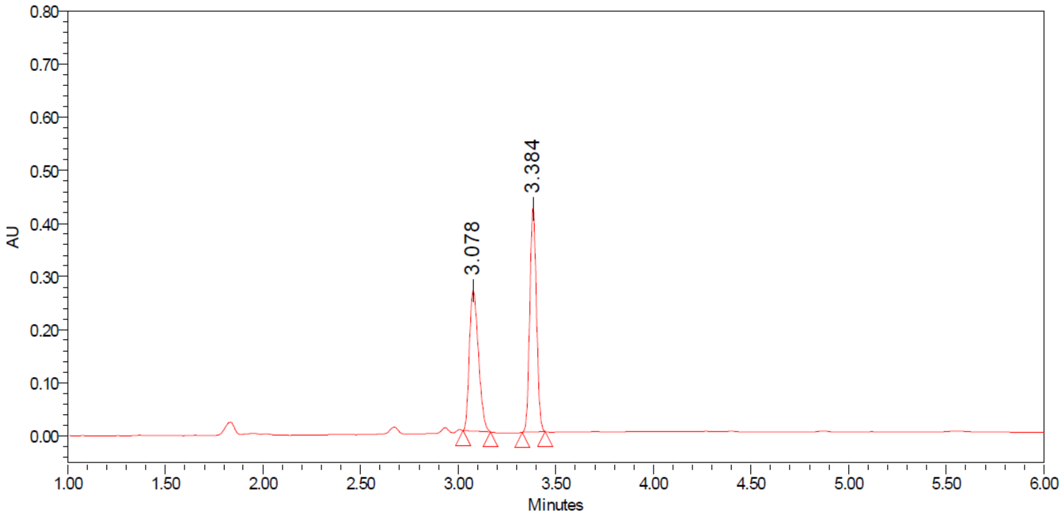

Peak Results

	RT
1	3.078
2	3.384

Enantiomerically enriched sample

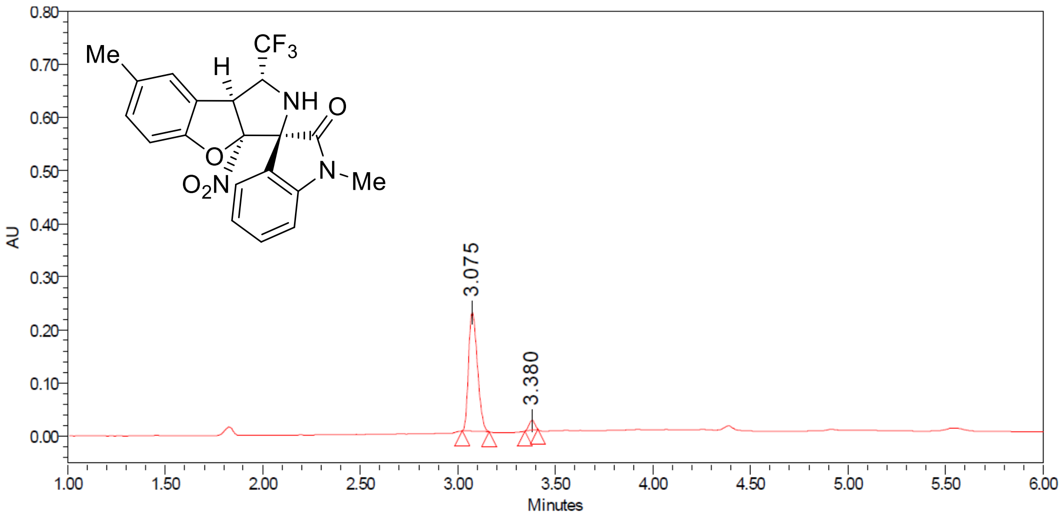

Peak Results

	RT	% Area
1	3.075	95.00
2	3.380	5.00

(1*R*,3*S*,3a*S*,8b*S*)-7-(*tert*-Butyl)-1'-methyl-3a-nitro-1-(trifluoromethyl)-1,2,3a,8b-tetrahydrospiro[benzofuro[2,3-c]pyrrole-3,3'-indolin]-2'-one (3n)

Racemic sample

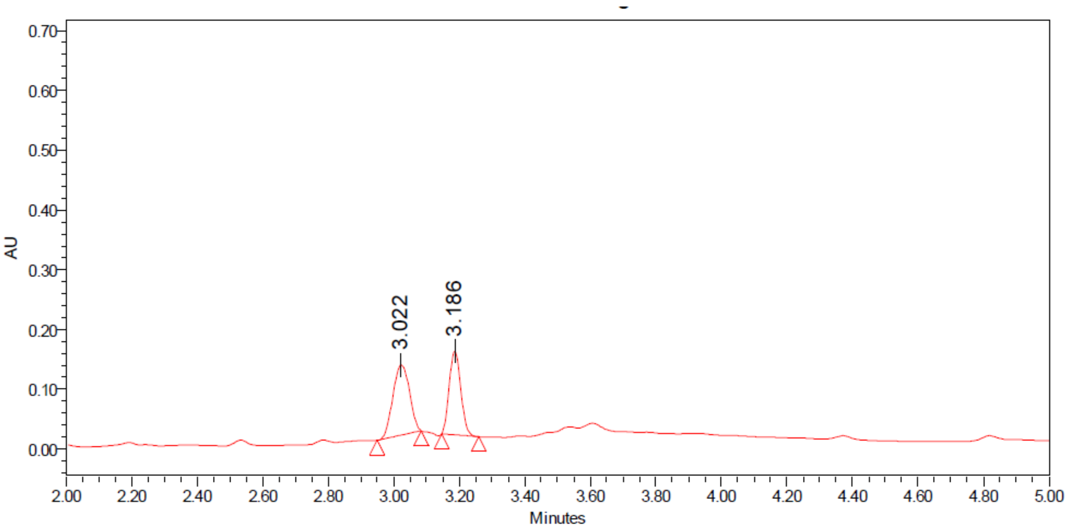

Peak Results

	RT
1	3.022
2	3.186

Enantiomerically enriched sample

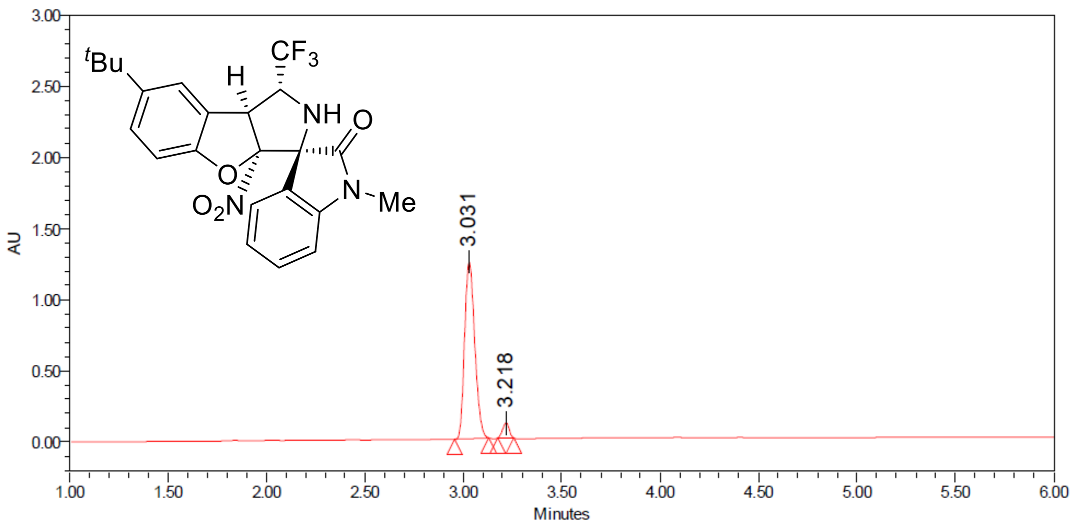

Peak Results

	RT	% Area
1	3.031	94.88
2	3.218	5.12

(1*R*,3*S*,3a*S*,8b*S*)-7-Chloro-1'-methyl-3a-nitro-1-(trifluoromethyl)-1,2,3a,8b-tetrahydrospiro[benzofuro[2,3-c]pyrrole-3,3'-indolin]-2'-one (3o)

Racemic sample

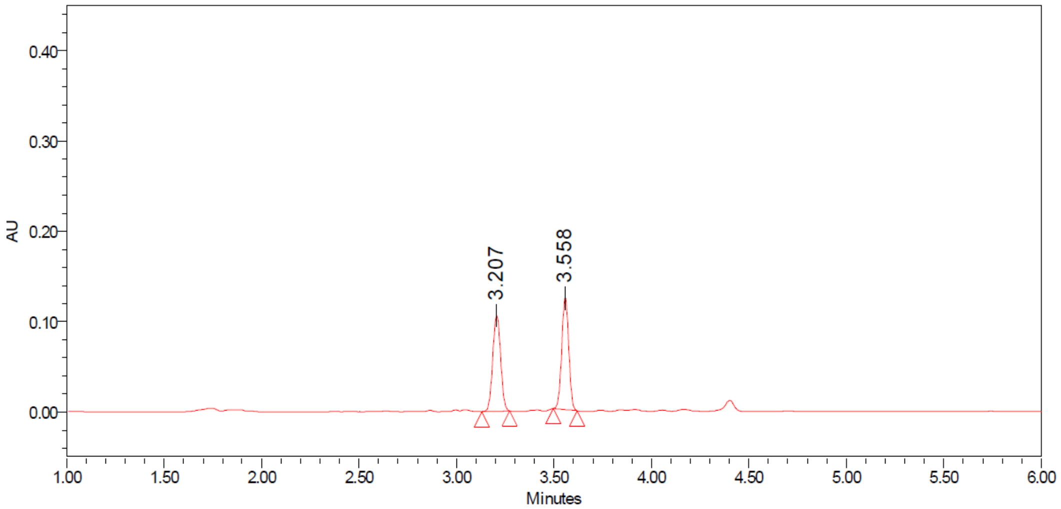

Peak Results

	RT
1	3.207
2	3.558

Enantiomerically enriched sample

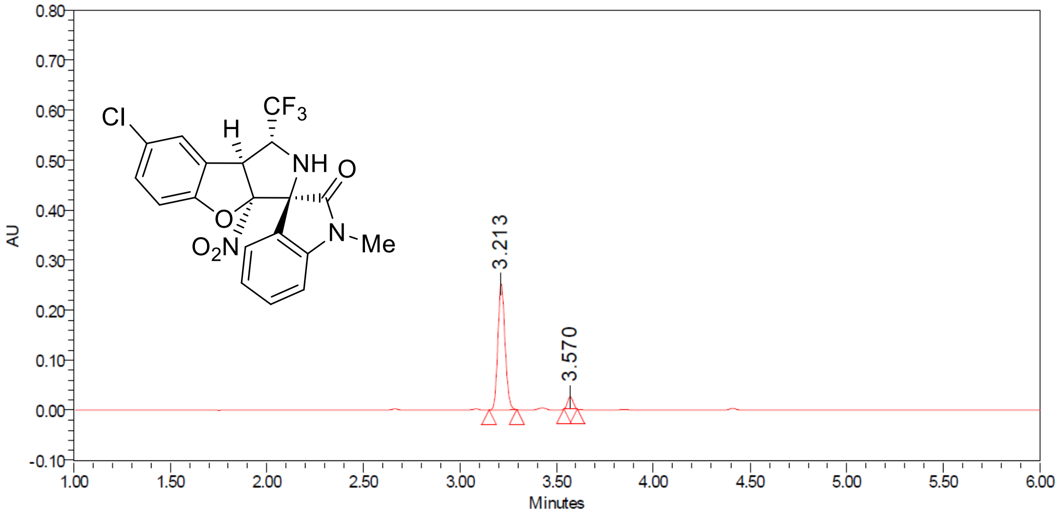

Peak Results

	RT	% Area
1	3.213	92.94
2	3.570	7.06

(1*R*,3*S*,3a*S*,8b*S*)-1'-Methyl-3a,7-dinitro-1-(trifluoromethyl)-1,2,3a,8b-tetrahydrospiro[benzofuro[2,3-c]pyrrole-3,3'-indolin]-2'-one (3p)

Racemic sample

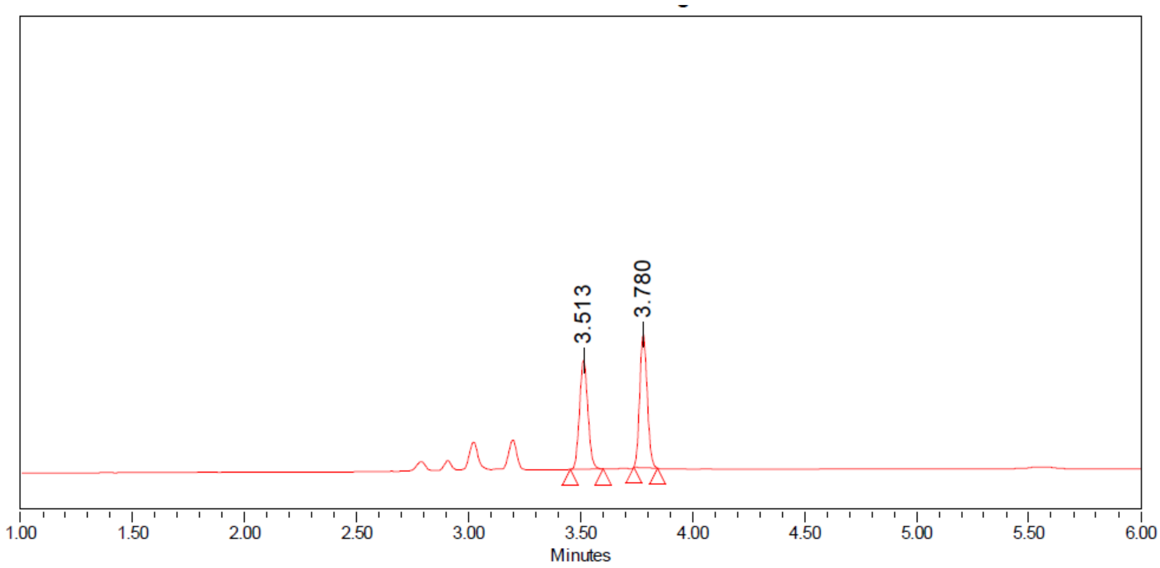

Peak Results

	RT
1	3.513
2	3.780

Enantiomerically enriched sample

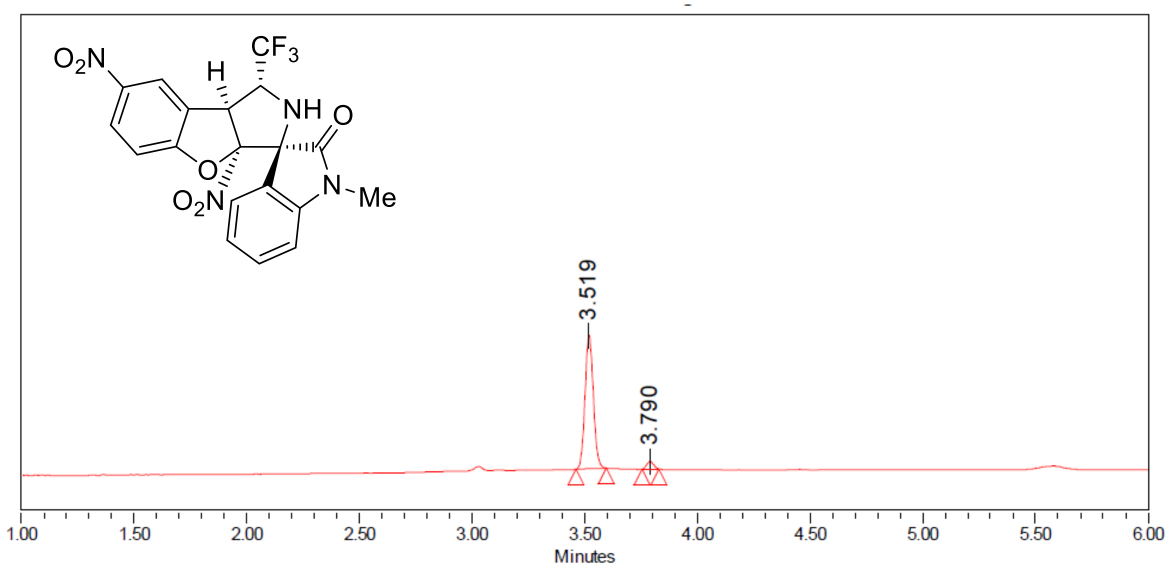

Peak Results

	RT	% Area
1	3.519	95.60
2	3.790	4.40

(1*R*,3*S*,3a*S*,8b*S*)-7-Bromo-1'-methyl-3a-nitro-1-(trifluoromethyl)-1,2,3a,8b-tetrahydrospiro[benzofuro[2,3-c]pyrrole-3,3'-indolin]-2'-one (3q)

Racemic sample

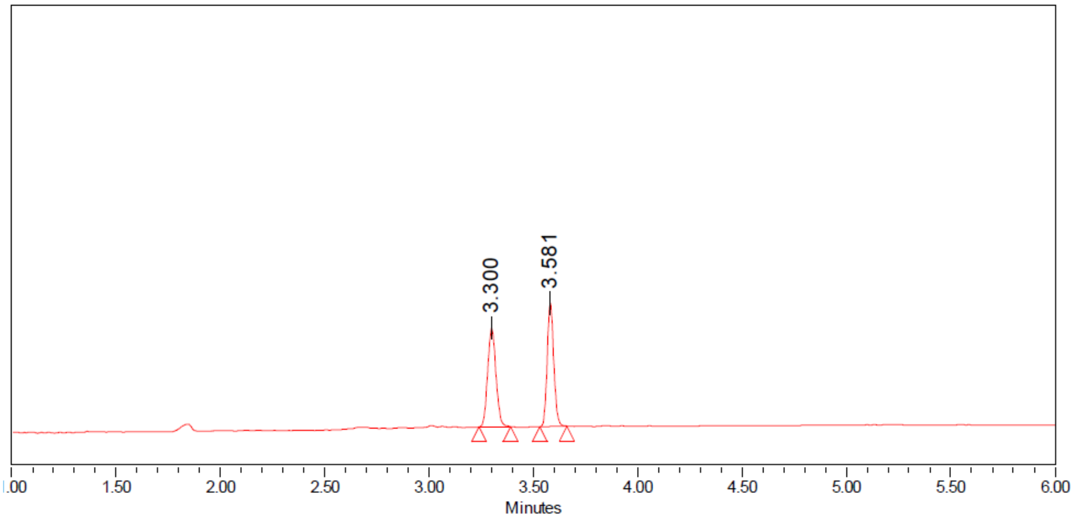

Peak Results

	RT
1	3.300
2	3.581

Enantiomerically enriched sample

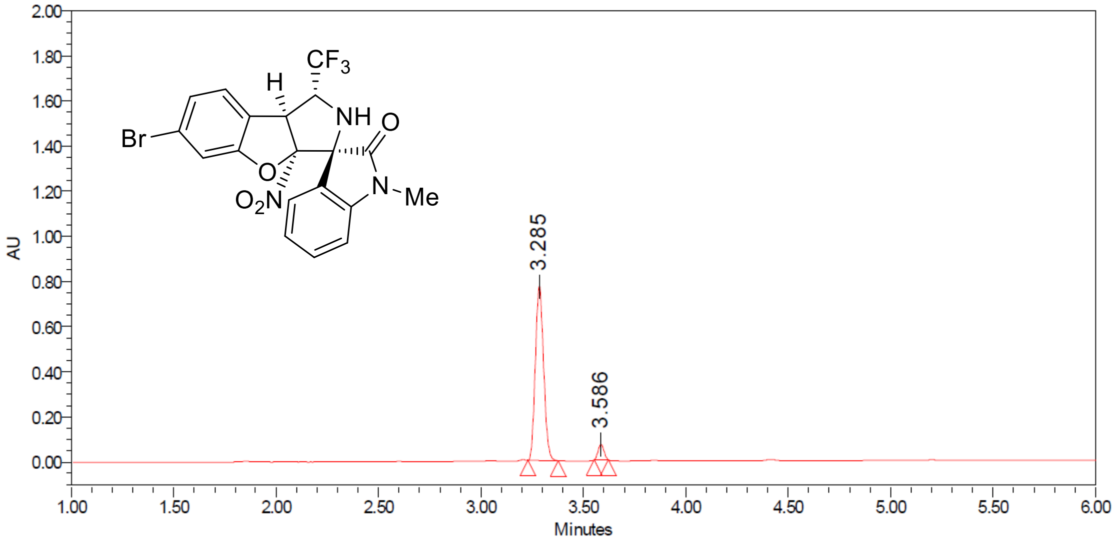

Peak Results

	RT	% Area
1	3.285	94.03
2	3.586	5.97

(1*R*,3*R*,3a*S*,8b*S*)-1'-Methyl-3a-nitro-1-(trifluoromethyl)-1,2,3a,8b-tetrahydrospiro[benzo[4,5]thieno[2,3-c]pyrrole-3,3'-indolin]-2'-one (3r)

Racemic sampe

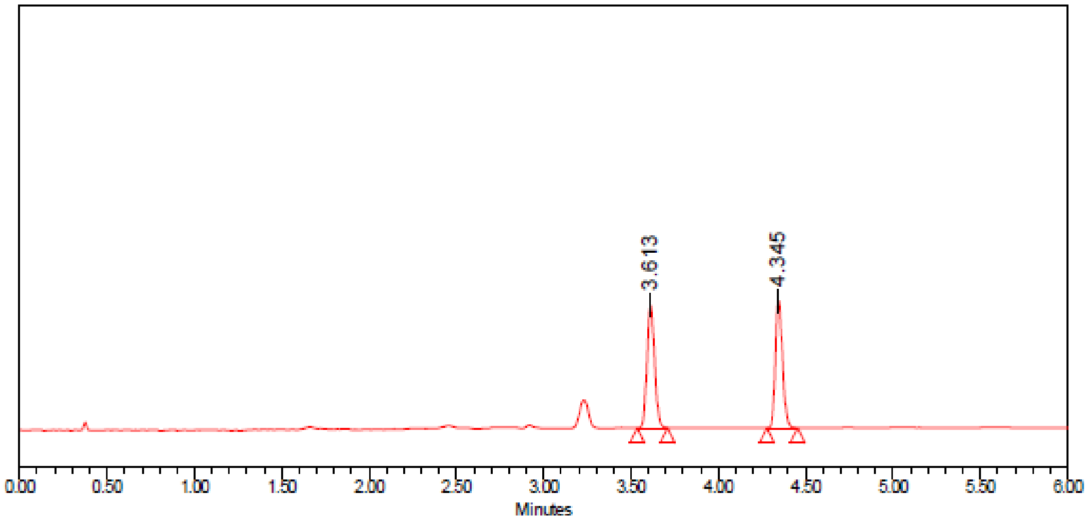

Peak Results

	RT
1	3.613
2	4.345

Enantiomerically enriched sample

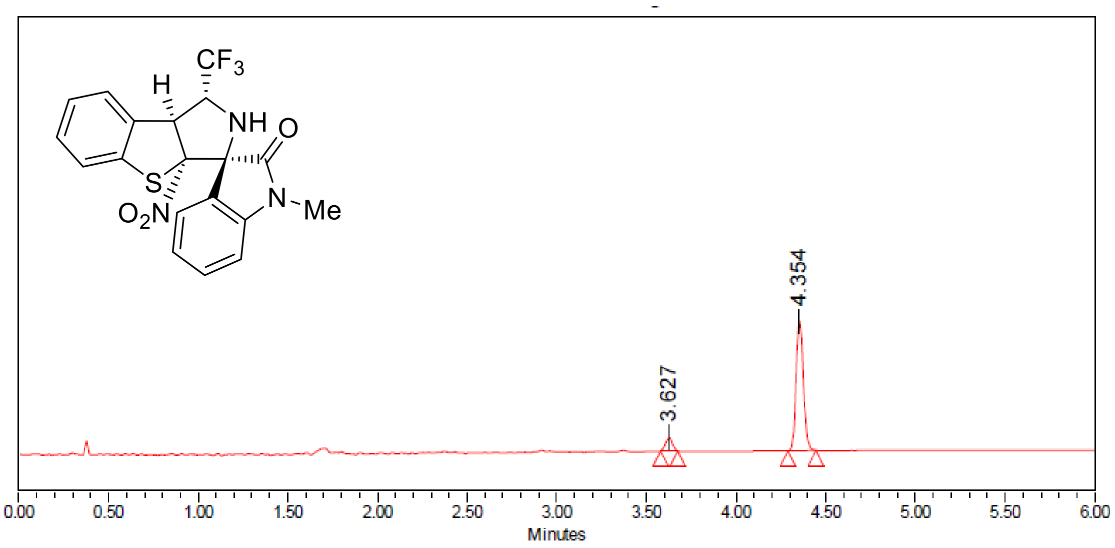

Peak Results

	RT	% Area
1	3.627	8.57
2	4.354	91.43
